# Virulence characterization and comparative genomics of *Listeria monocytogenes* sequence type 155 strains

**DOI:** 10.1186/s12864-020-07263-w

**Published:** 2020-11-30

**Authors:** Eva Wagner, Andreas Zaiser, Rebekka Leitner, Narciso M. Quijada, Nadja Pracser, Ariane Pietzka, Werner Ruppitsch, Stephan Schmitz-Esser, Martin Wagner, Kathrin Rychli

**Affiliations:** 1 Institute of Food Safety, Food Technology and Veterinary Public Health, University of Veterinary Medicine Vienna, Vienna, Austria; 2 Nofima - Norwegian Institute of Food, Fisheries and Aquaculture Research, Ås, Norway; RL: Evologic Technologies GmbH, Vienna, Austria; 3 Division of Microbial Ecology, Department of Microbiology and Ecosystem Science, University of Vienna, Vienna, Austria; 4 FFoQSI GmbH – Austrian Competence Centre for Feed and Food Quality, Safety and Innovation, Tulln, Austria; 5 Austrian National Reference Center for Listeria, Austrian Agency for Health and Food Safety, Vienna, Austria; 6 Department of Animal Science, Iowa State University, Ames, IA USA

**Keywords:** *Listeria monocytogenes*, ST155, Comparative genomics, Virulence potential, PrfA

## Abstract

**Background:**

*Listeria* (*L*.) *monocytogenes* strains show a high diversity regarding stress tolerance and virulence potential. Genome studies have mainly focused on specific sequence types (STs) predominantly associated with either food or human listeriosis. This study focused on the prevalent ST155, showing equal distribution among clinical and food isolates. We evaluated the virulence potential of 20 ST155 strains and performed comparative genomic analysis of 130 ST155 strains isolated from food, food processing environments and human listeriosis cases in different countries and years.

**Results:**

The in vitro virulence assays using human intestinal epithelial Caco2 and hepatocytic HEPG2 cells showed an impaired virulence phenotype for six of the 20 selected ST155 strains. Genome analysis revealed no distinct clustering of strains from the same source category (food, food processing environment, and clinical isolates). All strains harbored an intact *inlA* and *inlB* locus, except four strains, which had an internal deletion in the *inlA* gene. All strains harbored LIPI-1, but *prfA* was present in a longer variant in six strains, all showing impaired virulence. The longer PrfA variant resulted in lower expression of *inlA*, *inlB,* and *prfA*, and no expression of *hly* and *actA*.

Regarding stress-related gene content, SSI-1 was present, whereas *qacH* was absent in all strains. 34.6% of the strains harbored a plasmid. All but one ST155 plasmids showed high conservation and harbored *cadA2*, *bcrABC*, and a triphenylmethane reductase.

**Conclusions:**

This study contributes to an enhanced understanding of *L. monocytogenes* ST155 strains, being equally distributed among isolates from humans, food, and food processing environments. The conservation of the present genetic traits and the absence of unique inherent genetic features makes these types of STs especially interesting since they are apparently equally adapted to the conditions in food processing environments, as well as in food as to the human host environment. However, a ST155-specific mutation resulting in a longer PrfA variant impaired the virulence potential of several ST155 strains.

**Supplementary Information:**

The online version contains supplementary material available at 10.1186/s12864-020-07263-w.

## Background

*Listeria* (*L*.) *monocytogenes* is a foodborne bacterial human pathogen of significant concern as it can cause listeriosis, a rare disease associated with high rates of morbidity and mortality in vulnerable populations such as immunocompromised and/or elderly people, pregnant women and infants [1, 2]. *L. monocytogenes* is able to withstand a broad range of environmental stress conditions, including wide pH and temperature ranges, high salt concentrations, and low water activity. Moreover, it has also adapted to sanitizers and disinfection agents used in food processing environments, e.g. quaternary ammonium compounds (QACs), hydrogen peroxide, peracetic acid, and sodium hypochlorite [3–5].

There is a high degree of strain divergence regarding environmental adaptation, stress response, and virulence potential. Particular prevalence patterns are observed for clonal complexes (CC), which are consortia of multilocus sequence types that share a recent common ancestor. Strains of CC1, CC2, CC4, and CC6 are highly abundant among human clinical listeriosis cases but underrepresented among food isolates [6]. Specific genetic elements have been identified that contribute to the virulence potential of *L. monocytogenes*. An intact genomic locus harboring the internalins *inlA* and *inlB* mediates receptor-dependent entry into intestinal epithelial cells and various cell types like hepatocytes, epithelial and endothelial cells. Additionally, the *Listeria* Pathogenicity Islands (LIPI) 1, LIPI-3, LIPI-4, and Stress Survival Islet 1 (SSI-1) contribute to increased competitiveness in the gastrointestinal tract and promote systemic infection [7–10].

On the contrary, strains of CC9 and CC121 are highly abundant among isolates from food and the food processing environment but are underrepresented in clinical isolates [6, 11, 12]. Certain conserved genetic traits have been distinctly linked to phenotypes associated with stress tolerance in *L. monocytogenes* strains predominantly isolated from food processing environments. These include *bcrABC* and *qacH,* both confering increased tolerance towards benzalkonium chloride, *cadAC*, which contributes to increased tolerance towards cadmium, and SSI-2, which promotes survival under alkaline and oxidative stress conditions [13–16]. In addition, truncation variants of *inlA* have been described in these CCs that impair the successful access to the primary entry site, the intestinal epithelium, and thereby decrease the infection risk [17, 18].

Furthermore, some CCs are approximately equally distributed among isolates from food, food processing environment, and human cases. They include, among others, CC3, CC5, CC8–16, CC37, and CC155 [6, 11]. *L. monocytogenes* strains belonging to these CCs possibly transition from a saprophytic lifestyle to a host-associated lifestyle [19]. Genetic characteristics that could be potentially associated with these types of CCs have not been investigated extensively until now. This study focuses on *L. monocytogenes* ST155 strains, assigned to CC155, showing a globally increasing frequency since 2000 [11]. Strains of CC155 are among the ten most abundant CCs and have been frequently isolated from fish and fishery products [6, 20].

We detected that several ST155 strains were unable to proliferate in human intestinal epithelial Caco2 and macrophage-like THP1 cells in a previous study [21]. The aim of the present study was to assess the genetic background of this defective virulence phenotype and to subsequently elucidate the genetic diversity of *L. monocytogenes* ST155 strains. Therefore, we determined the in vitro virulence potential of 20 available *L. monocytogenes* ST155 strains using human intestinal epithelial Caco2 cells and human HEPG2 hepatocytes. To assess the genomic diversity, a total of 130 *L. monocytogenes* ST155 genomes were compared using phylogenetic and pangenome analyses based on whole-genome sequencing data. The differential gene content between the strains, as well as the characteristics of 95 virulence genes and 69 stress-associated genes, were investigated. Additionally, the expression of *prfA* and the PrfA-dependent genes *actA*, *hly*, *inlA,* and *inlB* was quantified in selected strains.

## Results

### Virulence characteristics of *Listeria monocytogenes* ST155 strains

To characterize the in vitro virulence potential, the invasion efficiency and intracellular growth of 20 *L. monocytogenes* ST155 strains, including nine clinical and eleven food isolates (Table 1), were determined in human intestinal epithelial Caco2 cells and human HEPG2 hepatocytes.

**Table 1:** Information on the *Listeria monocytogenes* ST155 strains sequenced in this study

Nr.	strain ID	source category	country	year	number of contigs > 500 bp	assembly size (bp)	GC content (%)	L50	N50 (K)	number of coding sequences	number of RNAs	Source
1	**CDL65**	food	unknown	unknown	110	3,052,261	38.4	3	543	3093	62	Vetmed
2	**CDL69**	food	unknown	unknown	69	3,057,911	37.9	8	125	3112	62	Vetmed
3	**L23–13**	clinical	Austria	2013	32	3,006,418	37.8	4	199	3044	59	Vetmed
4	**L6–13**	clinical	Austria	2013	25	3,005,715	37.8	3	429	3042	62	Vetmed
5	**MRL-14-00459**	clinical	Austria	2014	15	2,919,307	37.9	2	614	2916	66	AGES
6	**MRL-14-00747**	clinical	Austria	2014	100	2,987,154	37.9	17	580	3019	67	AGES
7	**MRL-15-00934**	clinical	Austria	2015	56	3,014,892	37.9	10	110	3036	66	AGES
8	**MRL-16-00194**	food	Austria	2016	227	2,898,292	37.9	38	24	2982	28	AGES
9	**MRL-16-00520**	food	Austria	2016	26	2,894,093	38.0	3	489	2871	46	AGES
10	**MRL-17-00171**	clinical	Austria	2017	17	2,934,109	37.9	3	544	2929	65	AGES
11	**MRL-17-00327**	food	Austria	2017	20	2,965,791	37.9	3	477	2986	65	AGES
12	**MRL-17-00443**	clinical	Austria	2017	81	2,999,831	37.8	14	70	3062	66	AGES
13	**MRL-17-01328**	clinical	Austria	2017	49	2,897,233	37.9	10	104	2888	64	AGES
14	**P06_14**	food	Brazil	2013	38	2,970,699	38.0	3	577	2983	62	Vetmed
15	**P12_10**	food	Turkey	unknown	51	2,944,502	37.9	4	185	2955	53	Vetmed
16	**Ro05**	food	Republic of Moldova	2012	152	3,063,567	38.1	19	56	3156	58	Vetmed
17	**Ro07**	food	Republic of Moldova	2012	203	3,064,181	38.1	25	34	3174	46	Vetmed
18	**Ro09**	food	Republic of Moldova	2012	94	2,997,747	38.1	5	174	3050	65	Vetmed
19	**Ro15**	food	Republic of Moldova	2013	168	3,069,348	38.0	24	40	3171	53	Vetmed
20	**SLCC0538**	clincial	Canada	1957	24	2,891,044	37.9	3	513	2876	66	Vetmed

Vetmed: Unit for Food Microbiology, University of Veterinary Medicine Vienna, AGES: Austrian National Reference Center for *Listeria*, Austrian Agency for Health and Food Safety

**Fig. 1:**
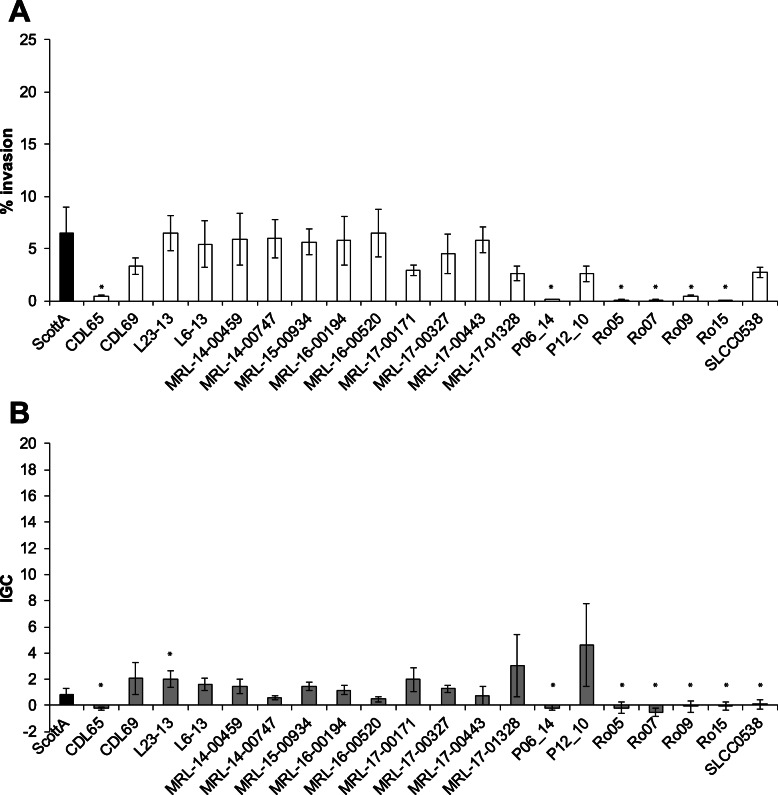
Virulence potential of 20 *L. monocytogenes* ST155 strains in human intestinal epithelial Caco2 cells. Invasion efficiency (%, **a**) and intracellular growth coefficient (IGC, **b**) are shown as mean value ± SD of three biological replicates determined in duplicates. * statistically significant differences compared to the ScottA variant (*p* < 0.05)

The isolates revealed different invasion efficiencies in Caco2 cells (Fig. 1a). Strains CDL65, P06_14, Ro05, Ro07, Ro09, and Ro15 showed significantly lower invasion efficiencies (0.08–0.51%) compared to the ScottA variant (ST2), whereas the invasion efficiency of all other strains was comparable to that of the ScottA variant (2.61–6.55%).

The intracellular growth coefficient (IGC) of seven strains (CDL65, P06_14, Ro05, Ro07, Ro09, Ro15, and SLCC0538) was significantly lower and mostly negative (− 0.21–0.06) compared to the ScottA variant (Fig. 1b), indicating that these strains are not able to proliferate or even survive inside Caco2 cells. The IGCs of all other strains (0.48–4.65) were comparable to that of the ScottA variant.

**Fig. 2:**
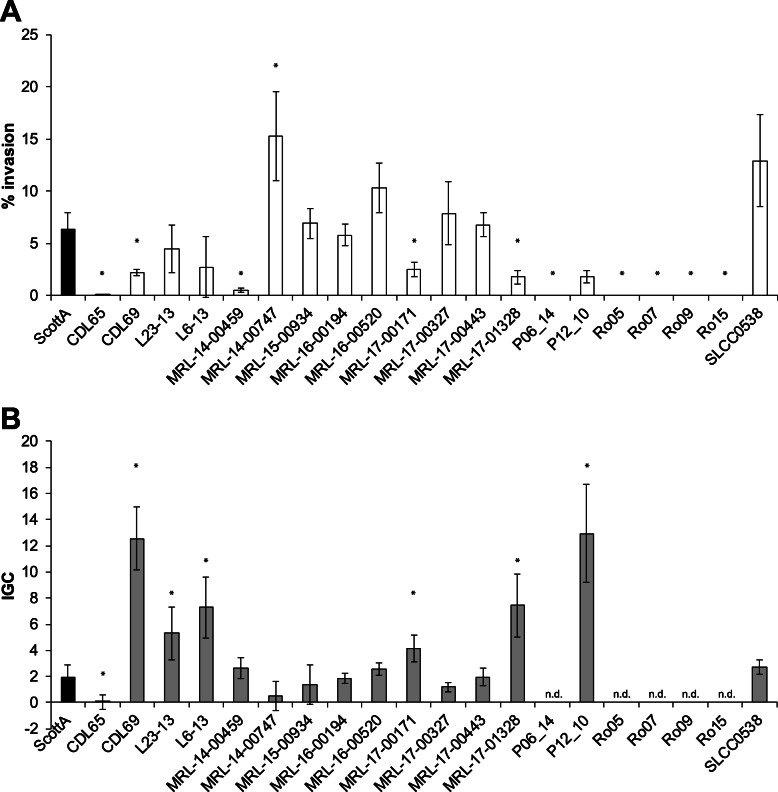
Virulence potential of 20 *L. monocytogenes* ST155 strains in human hepatocytic HEPG2 cells. Invasion efficiency (%, **a**) and intracellular growth coefficient (IGC, **b**) are shown as mean value ± SD of three biological replicates determined in duplicates. * statistically significant differences compared to the ScottA variant (*p* < 0.05)

The invasion efficiencies in HEPG2 cells showed a high strain variability ranging from 0 to 15.23% (Fig. 2a). We observed six strains (CDL65, P06_14, Ro05, Ro07, Ro09, and Ro15) unable to invade into HEPG2 cells. Furthermore, the invasion efficiencies of the strains CDL69, MRL-14-00459, MRL-17-00171, and MRL-17-01328 were significantly lower, whereas the invasion efficiency of strain MRL-14-00474 was significantly higher compared to the ScottA variant.

A high strain variability was also observed with respect to the intracellular growth of the strains (0.47–12.94, Fig. 2b). The strains CDL69, L23–13, L6–13, MRL-17-00171, MRL-17-01328, and P12_10 showed significantly higher IGCs, whereas strain CDL65 showed a significantly lower IGC compared to the ScottA variant. The IGCs of strains P06_14, Ro05, Ro07, Ro09, and Ro15 were not determinable since invasion is a prerequisite for intracellular growth.

### Genomic features of *Listeria monocytogenes* ST155 strains

We sequenced the genomes of the 20 *L. monocytogenes* ST155 strains used for the virulence assays (Table 1) and retrieved the genomes of 110 ST155 strains from NCBI GenBank (Table S1). The collection included strains from 13 different countries and different years of isolation (1957–2019) originating from human clinical listeriosis patients (*n* = 37), food (*n* = 40), and food processing environments (*n* = 38). The source of 15 strains was unknown. Moreover, the year of isolation of 15 strains and the country of isolation of 14 strains was not specified.

The *L. monocytogenes* ST155 genomes ranged from 2.84 to 3.18 Mbp. The phylogenetic relationship between the *L. monocytogenes* ST155 strains was assessed by pangenomic analysis based on the presence and absence of genes between the strains. Overall, 6422 genes were identified, comprising 2562 core genes (present in ≥99% of the strains) and 3860 accessory genes.

**Fig. 3:**
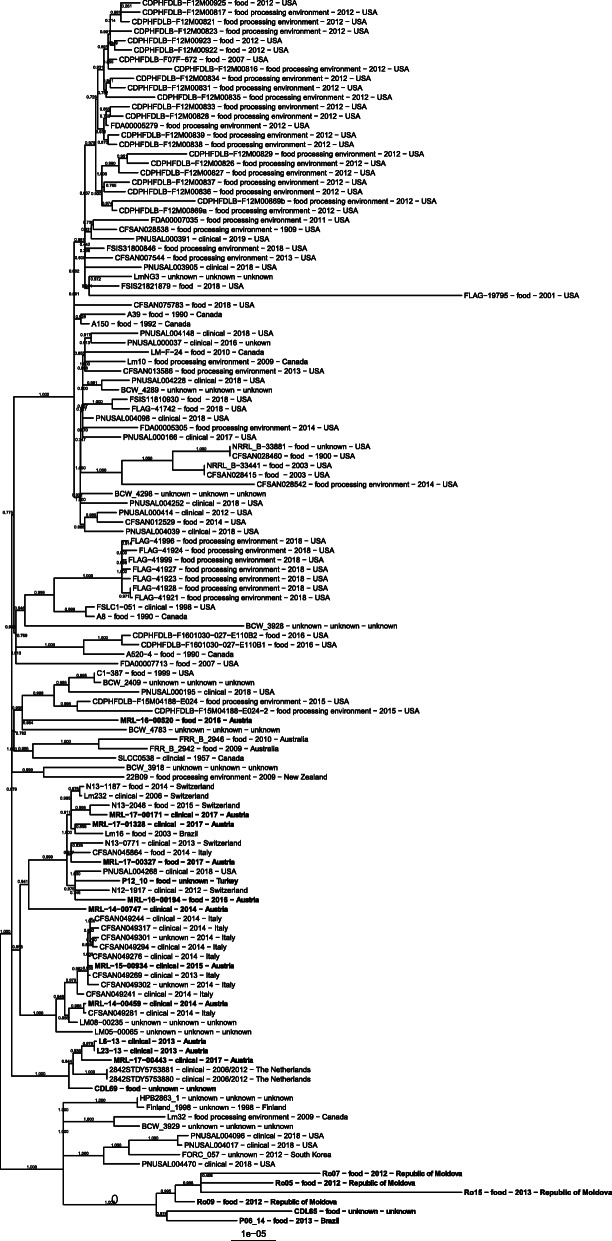
Phylogenetic analysis of 130 *L. monocytogenes* ST155 strains based on core gene alignment. The phylogenetic tree was rooted to mid-point. The 20 isolates that were used for the virulence characterization are highlighted in bold

The similarities based on the core genes’ sequence alignment are visualized as a phylogram in Fig. 3. Strikingly, the six strains showing impaired in vitro virulence (CDL65, P06_14, Ro05, Ro07, Ro09, and Ro15) clustered. The SNP distances between the core genes of these six strains ranged from 130 to 368 (Table S2). In addition, the CDPHFDLB and FLAG (Florida) isolates from the United States notably clustered according to the source of isolation and isolation year. There was, however, no overall evident clustering of food and human clinical strains, respectively.

### Genome-wide analysis of virulence genes

**Fig. 4:**
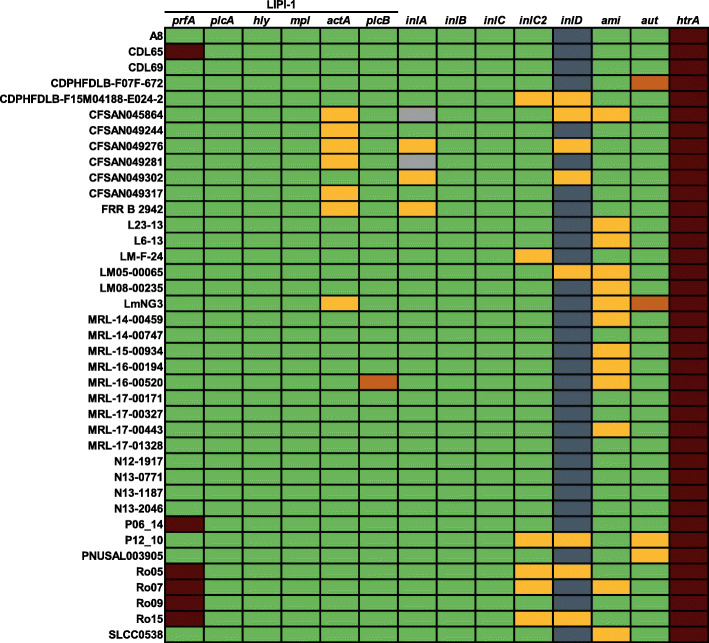
Distribution and variability of virulence factors of selected *L. monocytogenes* ST155 strains. Green: full length, red: longer variant, dark grey: shorter variant, light grey: internal deletion, orange: truncation, yellow: incomplete sequence

We conducted a genome-wide analysis of all 130 *L. monocytogenes* ST155 strains for 95 genes associated with virulence (Fig. 4, Fig S1 and Table S3).

**Fig. 5:**
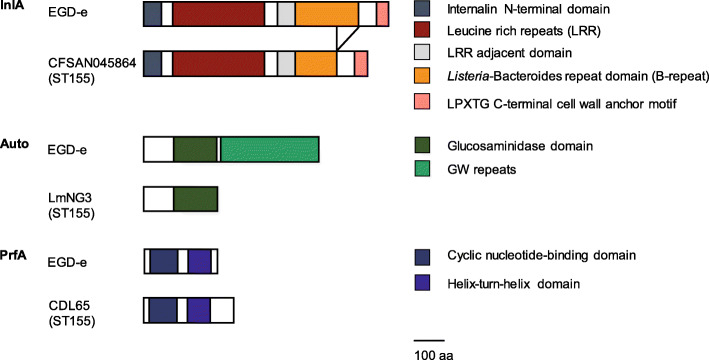
Variants of InlA, Auto and PrfA in selected *L. monocytogenes* ST155 strains compared to their EGD-e homologs

Internalin A and internalin B are the most prominent virulence factors of *L. monocytogenes* as they mediate the receptor-dependent entry into host cells. The nucleotide identity for *inlA* ranged from 98.19 to 99.21% compared to the sequence of EGD-e. Four strains harbored an internal deletion in the *inlA* gene (Fig. 5 and Fig S1-2). Since the genomes of these strains were retrieved from the NCBI database, these strains were not tested for the in vitro virulence. A full-length *inlB* was present in all strains with a nucleotide identity of 99.63% compared to EGD-e. *inlC,* which encodes for the secreted internalin C that promotes cell-cell spread in polarized epithelial cells, was present in full-length in all strains with a nucleotide identity between 99.55 and 99.66% compared to the EGD-e homolog [22]. While *inlH* was absent, all ST155 strains harbored a full-length *inlC2* (nucleotide identity between 92.96 and 93.02% with the sequence of F2365) and a shorter version of *inlD* lacking a glutamate at amino acid position 30 with a nucleotide identity between 89.75 and 90.69% shared with *inlD* of *L. monocytogenes* F2365 [23]. The genes *inlP1* and *inlP3* were absent in all ST155 strains.

Moreover, a conserved longer variant of the *htrA* gene encoding a serine protease was found in all 130 strains (Fig. S1). This variant, also found in the *L. monocytogenes* strain 10403S, shared a nucleotide identity between 98.87 and 98.94% with *htrA* of strain EGD-e. This longer HtrA variant had an additional threonine at amino acid position 99 compared to the HtrA of *L. monocytogenes* EGD-e, F2365 and ScottA (Fig. S3). We identified a premature stop codon in the *aut* gene after 726 nucleotides (242 amino acids) in 31 ST155 strains (Fig. 5 and Fig, S4). The *aut* gene (nucleotide identity between 99.77 and 99.83% with EGD-e) encodes the surface-associated autolysin Auto.

Additionally, all 130 *L. monocytogenes* ST155 strains harbored LIPI-1 but not LIPI-3 or LIPI-4. LIPI-1 consists of the genes *actA* (actin-assembly inducing protein, nucleotide identity between 98.25 and 99.17% with EGD-e), *hly* (listeriolysin O, nucleotide identity between 98.99 and 99.06% with EGD-e), *mpl* (metalloprotease, nucleotide identity between 99.74 and 99.80% with EGD-e), *plcA* (phosphatidylinositol-specific phospholipase C, nucleotide identity between 98.85 and 98.95% with EGD-e), *plcB* (phospholipase C, nucleotide identity of 99.77% with EGD-e) and *prfA* (positive regulatory factor A, nucleotide identity between 99.86 and 100% with EGD-e) [6, 7, 24]. One strain (MRL-16-00520) harbored a truncated variant of PlcB due to a premature stop codon after 181 amino acids (543 nucleotides).

Intriguingly, the six strains (CDL65, P06_14, Ro05, Ro07, Ro09, and Ro15) that showed a deficient in vitro virulence potential harbored a longer *prfA* variant due to a deletion of five nucleotides (AAATT) at position 708–712, which includes the first base of the stop codon (TAA). This leads to a new stop codon at position 881, resulting in 293 instead of 237 amino acids (Fig. 5 and Fig S5). De novo in silico 3D protein structure prediction revealed that the longer PrfA variant harbors an additional α-helix and coiled region at the C-terminus (Fig. S6).

All other analyzed genes were present in conserved variants in all 130 ST155 strains.

### The longer PrfA variant leads to decreased expression of PrfA-dependent genes

**Fig. 6:**
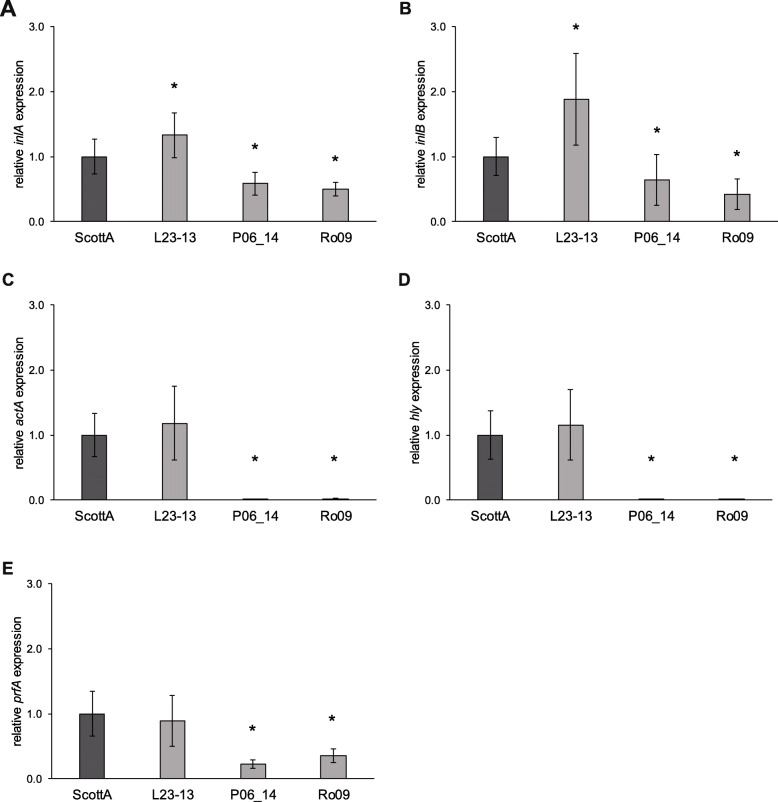
mRNA expression of *inlA* (**a**), *inlB* (**b**), *actA* (**c**), *hly* (**d**) and *prfA* (**e**) of *L. monocytogenes* ScottA variant, L23–13, P06_14 and Ro09. Values were normalized to *16S rRNA* expression levels and are represented as x-fold of the ScottA variant. Data represent mean values ± SD of three biological replicates, performed in triplicates and measured in duplicates. * statistically significant differences compared to the control strain ScottA variant (*p* < 0.05)

To characterize the impact of the longer PrfA variant on gene expression in *L. monocytogenes*, the expression of the *prfA* and the PrfA-dependent genes *actA*, *hly*, *inlA,* and *inlB* was quantified using qRT-PCR. Two representative strains, P06_14, and Ro09, harboring the longer PrfA variant, as well as the ScottA variant and the clinical strain L23–13, both harboring the wildtype PrfA and showing equal invasion efficiencies and IGCs in Caco2 and HEPG2 cells, were selected for the analysis (Fig. 6). *L. monocytogenes* QOC1 wildtype and a corresponding *prfA* deletion mutant were additionally used as controls.

The expression of *inlA* and *inlB* was significantly reduced to approximately half the expression level in strains P06_14 and Ro09 compared to the ScottA variant. In parallel, the expression of *inlA* and *inlB* was significantly lower (relative expression of 0.62 and 0.41) in the QOC1 *prfA* deletion mutant strain compared to the wildtype strain (data not shown). Strain L23–13, in contrast, showed a significantly higher expression of both genes compared to the ScottA variant (Fig. 6a and b).

The expression of *actA* and *hly* was significantly reduced to a minimum in P06_14 (relative expression of 0.011 and 0.004, respectively) and Ro09 (relative expression of 0.023 and 0.010, respectively) compared to the ScottA variant. There were no significant differences in the expression levels of *actA* and *hly* between L23–13 and the ScottA variant (Fig. 6c and d). The relative expression of *actA* and *hly* in the QOC1 *prfA* deletion mutant was 0.068 and 0.025 compared to QOC1 wildtype, respectively, and was thereby significantly minimized (data not shown). Moreover, *prfA* expression was significantly decreased in P06_14 (relative expression of 0.225) and Ro09 (relative expression of 0.353) compared to the ScottA variant, whereas no difference in *prfA* gene expression was observed between strain L23–13 and the ScottA variant (Fig. 6e). As expected, *prfA* was not expressed in the QOC1 *prfA* deletion mutant (data not shown).

### Genome-wide analysis of stress-associated genes

**Fig. 7:**
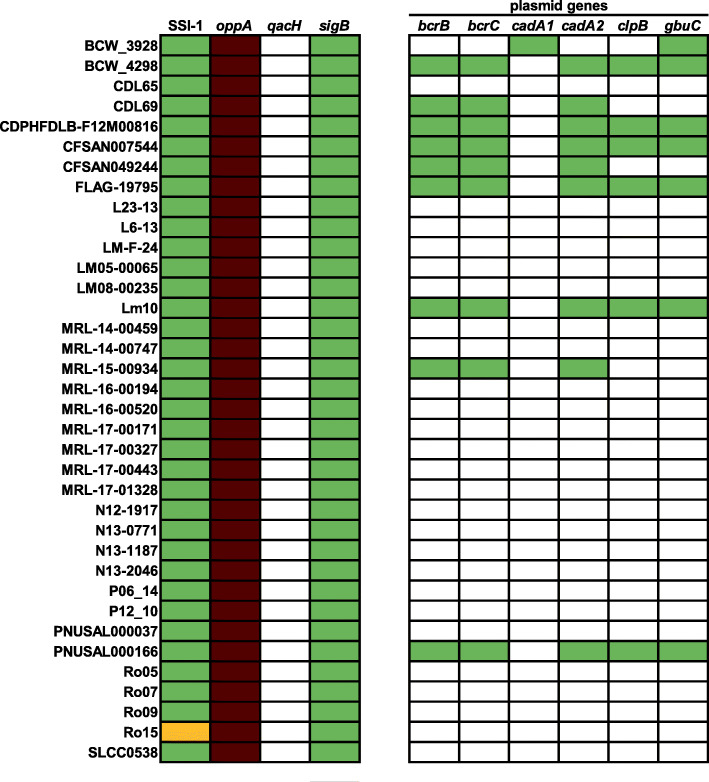
Distribution and variability of stress-associated factors among selected *L. monocytogenes* ST155 strains. Green: full length, white: absent, red: longer variant, yellow: incomplete sequence

To evaluate the stress survival potential of ST155 strains, all 130 genomes were screened for 69 genes associated with stress tolerance of *L. monocytogenes* (Fig. 7, Fig. S7 and Table S4). All strains harbored SSI-1 and concomitantly lacked SSI-2. The representative gene for SSI-1, *gadA*, encodes a glutamate decarboxylase and shares between 99.86 and 99.93% nucleotide identity with the sequence in EGD-e. *oppA* was present in a longer conserved variant in all strains encoding for 559 instead of 554 amino acids and shares 99.28% nucleotide identity with the sequence in EGD-e. The transposon *Tn6188* harboring *qacH* was absent in all strains.

Except for the plasmid-encoded genes *bcrABC* and *cadA* (see below), all of the remaining genes were found in full-length variants in all of the ST155 strains.

### Plasmids in ST155 strains

**Fig. 8:**
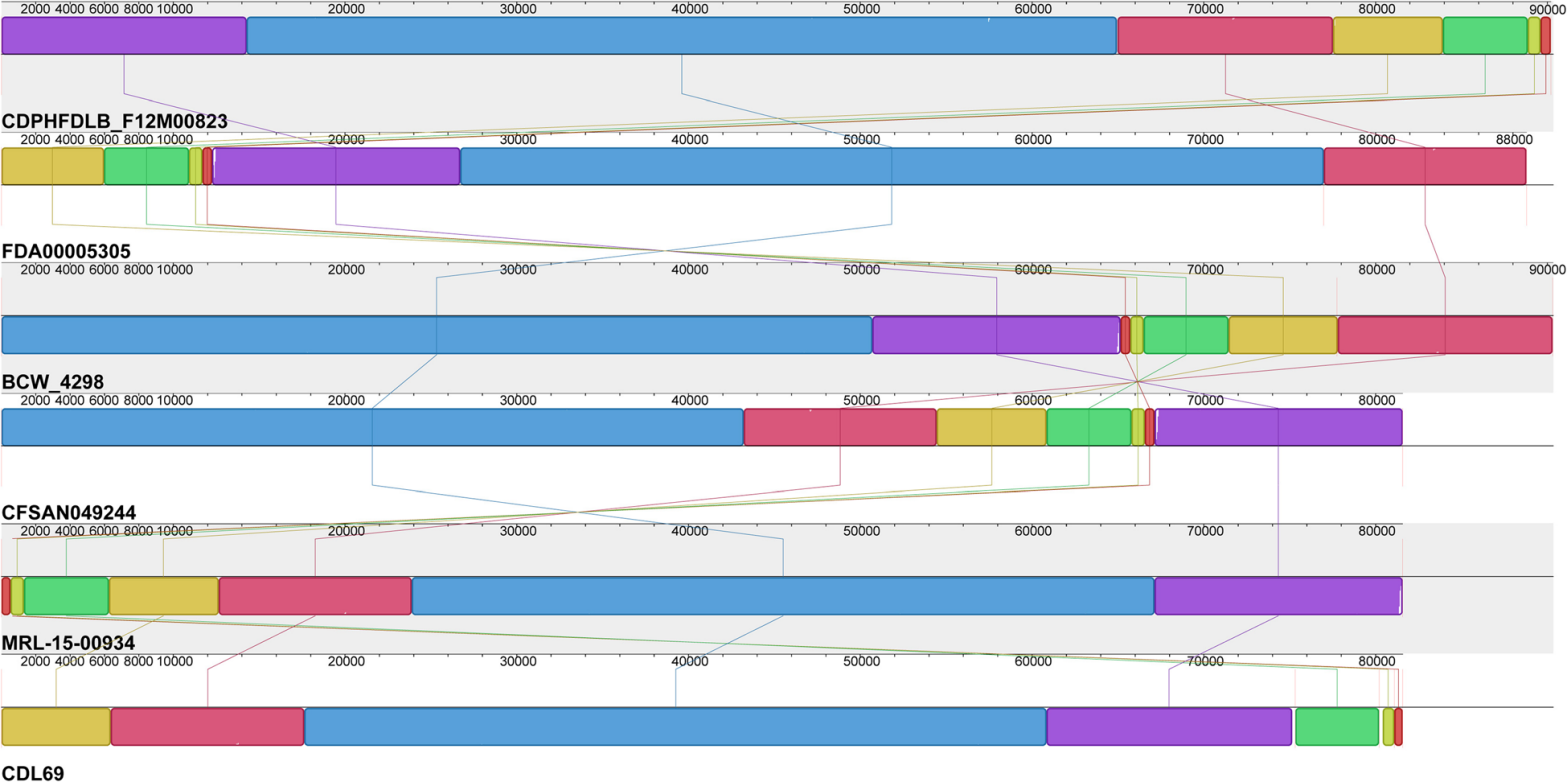
Alignment of selected *L. monocytogenes* ST155 plasmids. The plasmids were aligned with MAUVE [27]. Homologous regions have the same color and the height of the blocks correlates with the conservation level of the regions for each plasmid

Plasmids have been shown to provide advantages to *L. monocytogenes* under food and food production relevant stress conditions [25, 26]. Among the 130 strains in this study, 45 strains (34.6%) harbored a putative plasmid with sizes ranging from 57 to 92 kbp; the average size of ST155 plasmids in this study was 87.7 kbp. Except for the 57 kbp plasmid in strain BCW_3928, all other ST155 plasmids had sizes ranging from 80 to 92 kbp. Interestingly, all plasmids found were group 2 plasmids based on their plasmid replication protein RepA sequences; only the plasmid in strain BCW_3928 was a group 1 plasmid. Because of the high levels of differences between the BCW_3298 plasmids and all other ST155 plasmids, the BCW_3928 plasmid was excluded from the observations reported below. The ST155 plasmids were highly similar and showed only minor differences in their genetic content (Figs. 7 and 8, Table S5). The overall average nucleotide identity (ANI) of the ST155 plasmids was higher than 99.9%. An in-depth analysis of the plasmid contigs revealed the presence of several plasmid genes known to be involved in stress response (Table S5). All ST155 plasmids harbored *cadA2*, *bcrABC*, and *tmr*, encoding a triphenylmethane reductase. The only differences in genetic content between the ST155 plasmids were found in the presence or absence of a putative NADH peroxidase, *gbuC*, and a putative ClpB-like protein.

## Discussion

To determine intrinsic genetic attributes of *L. monocytogenes* ST155 strains, we characterized their virulence potential and subjected 130 ST155 genomes to comparative analysis, including the determination of phylogenetic relationships and the presence and characteristics of virulence and stress-associated factors.

For the determination of the in vitro virulence potential in human intestinal epithelial Caco2 cells and human HEPG2 hepatocytes, we selected eleven available food and nine human clinical ST155 isolates. Six strains (CDL65, P06_14, Ro05, Ro07, Ro09, and Ro15) displayed impaired virulence. These six strains showed very low invasion efficiencies and no intracellular proliferation in Caco2 cells and no invasion in HEPG2 cells, except for strain CDL65, with an invasion efficiency reduced to a negligible minimum. All of these strains were food isolates, four of them originated from the Republic of Moldova and one from Brazil. The origin of one strain was unknown. These six strains showed close genetic relationship and clustered in the phylogenetic analysis. The in vitro virulence results confirm the results from one of our previous findings stating that ST155 strains might have reduced invasion efficiency and impaired intracellular proliferation, not only in human Caco2 cells but also in human THP1 macrophages [21]. Several ST155 strains have also been reported to be non-hemolytic [28].

No association between the virulence potential in both cell types and the source of isolation (food and clinical) was found for the other ST155 strains tested for virulence in this study. In Caco2 cells, no differences in invasion and intracellular proliferation between the other strains were observed except for strain SLCC0538, which was able to invade the host cells but unable to proliferate intracellularly. In contrast, the virulence potential among the remaining *L. monocytogenes* ST155 strains isolated from food and human listeriosis cases was rather heterogeneous. Likewise, some strains like the clinical isolate MRL-14-00474 showed a high invasion efficiency but a low intracellular proliferation potential, while other strains such as the food isolate P12_10 showed a low invasion efficiency but a high intracellular proliferation potential. These findings indicate that the virulence potential of ST155 strains isolated from food and human listeriosis cases is consistent in Caco2 cells but strain-dependent in HEPG2 cells with some avirulent food isolates.

The strains included in this study represent a balanced source distribution of *L. monocytogenes* ST155 isolates from food, food processing environments, and human clinical isolates. These findings strongly support the hypothesis of ST155 being a sequence type that might be equally adapted to saprophytic and host-associated environments and able to unobstructedly transition between these two lifestyles. However, the source of strains is not equally distributed in the *L. monocytogenes* genome database. On the contrary, other studies report sequence types and clonal complexes strongly correlated with a majority of either food and environmental or clinical isolates sharing unique genetic attributes. CC9 and CC121, for instance, primarily comprise food and environmental isolates, while the incidence of clinical isolates is low. In addition, these CCs have genetic adaptations that promote their emergence and persistence in food processing environments but impairs their virulence potential [14–16, 18]. CC1, CC2, CC4, and CC6, on the other hand, include mostly clinical isolates, whereas the frequency of food and environmental isolates is low. These CCs harbor specific genes that contribute to an elevated virulence potential but lack genes that promote survival under different environmental stress conditions [6, 19].

Comparative studies have not yet been performed for sequence types equally distributed among different sources. The phylogenetic analysis highlighted an overall high degree of similarity based on the alignment of core genes of all evaluated *L. monocytogenes* ST155 strains regardless of the source of isolation. Some clusters are plausibly explainable due to shared isolation sites and years of isolation, like the FLAG (Florida) isolates from the USA. The six non-virulent isolates clustered.

Our genome-wide survey revealed a consistent and conserved incidence of the 95 analyzed virulence genes across all *L. monocytogenes* ST155 strains, independent of the isolation source. *inlA*, *inlB,* and *inlC* shared a high degree of conservation among the ST155 strains and with strain EGD-e. Despite this, four strains were found to harbor the same internal deletion in InlA, lacking 70 amino acids in the B-repeats domain. Two of these strains were clinical isolates, one was a food isolate, and the fourth strain source was unknown. To our knowledge, this internal InlA deletion has not been described previously. The low occurrence of this internal deletion indicates a sporadic event resulting in this mutation. Since the virulence potential of these four strains was not assessed in this study, it is tempting to speculate that the internal InlA deletion would affect the invasion efficiency in Caco2 cells. There is, however, evidence that the leucine-rich repeats and the inter-repeat region of InlA are sufficient to promote bacterial entry into cells expressing E-cadherin and that the B-repeats domain has a minor influence on virulence [29]. None of the ST155 strains harbored *inlH* but *inlC2* and *inlD,* which were highly conserved among ST155 strains. InlD was encoded in a conserved shorter variant in all ST155 strains lacking a glutamate at amino acid position 30 compared to strain F2365. These findings indicate that several virulence factors like *inlD* are conserved within one ST, whereas others like *inlA* show diversity not only between the STs, but also within one ST.

The serine protease HtrA, which is responsible for enhanced intracellular survival in macrophages, was also found in a conserved variant in all ST155 strains [30]. This variant, which encodes an additional threonine at amino acid position 99, is also present in strain 10403S. It shares a high nucleotide identity with strain EGD-e, which lacks this threonine residue. Since the additional amino acid does not affect the protease domains, we assume that it does not impact its function. It is more likely that the protease exists in two or more variants that are stochastically distributed across the *L. monocytogenes* population.

The virulence factor *aut* is important for entry into non-phagocytic cells and was present in all ST155 strains and shares a high nucleotide identity with strain EGD-e. However, *aut* was truncated due to a premature stop codon after 242 amino acids in 31 strains. 23 of those strains were isolated from food processing environments, six from food, one from a human clinical listeriosis case, and the source of one strain is unknown. As none of these strains was included in our in vitro virulence survey, we cannot exclude an effect of this truncation on the virulence potential. Worthy of note, *aut* is subjected to substantial differences in nucleotide and amino acid sequence and length in different *L. monocytogenes* strains. While EGD-e and 10403S have an *aut* gene that is 1719 bp, as described by Cabanes et al. [31], whereas the ScottA and F2365 *aut* is 2325 bp.

All ST155 strains encode LIPI-1, a pathogenicity island that consists of the genes *actA*, *hly*, *mpl*, *plcA*, *plcB,* and *prfA* which were highly conserved among ST155 and were highly similar to strain EGD-e. One food isolate (MRL-16-00520) had a truncation in *plcB* due to a premature stop codon after 181 amino acids. Although PlcB promotes vacuolar escape together with LLO and thereby contributes to intracellular replication, we did not detect any difference in the intracellular replication. Both PlcB variants encode for the zinc-dependent phospholipase C domain, but the one of MRL-16-00520 is shorter than the one of ScottA and EGD-e. Even if this variation affected the intracellular replication, the effect might either be masked by other virulence factors or be too small to be detectable in our in vitro assessment.

The six food isolates (CDL65, P06_14, Ro05, Ro07, Ro09, and Ro15), which showed impaired virulence, encoded a longer variant (882 bp) of the positive regulatory factor A, *prfA*, due to a deletion of five nucleotides at the C-terminus including the first base of the stop codon. BLAST analysis of the observed *prfA* variant against the *L. monocytogenes* whole genome sequence GenBank entries revealed that this mutation is restricted to strains of ST155 (all included in this study). As PrfA acts as the transcriptional regulator of LIPI-1 as well as of *inlA* and *inlB*, the evident absence of virulence in these six strains already suggested the non-functionality of this factor. Gene expression analysis showed that the expression of *inlA*, *inlB*, *actA*, *hly,* and *prfA* was significantly decreased in the ST155 strains harboring the longer PrfA variant compared to wildtype strains. The expression of the LIPI-1 genes *actA* and *hly* was almost entirely depleted. In contrast, the expression of *inlA* and *inlB* was only reduced. The obtained results are concomitant with the fact that *actA* and *hly* are under exclusive, proximal transcriptional control of PrfA and the absence of intracellular replication in Caco2 and HEPG2 cells [32]. *inlA* and *inlB,* on the contrary, are not exclusively transcriptionally regulated by PrfA but co-regulated by SigB [33]. The reduction of *prfA* expression was in accordance with the fact that *prfA* expression is regulated via a positive feedback loop besides SigA and SigB [34–37]. The same longer PrfA variant has recently also been observed in ST155 strains based on the hemolytic activity of LLO [28]. In accordance with our data, in these strains, all isolated from food, no *hly* expression was detectable resulting in a non-hemolytic phenotype. Unfortunately, genome data of these strains were not available in a public database.

In silico protein structure prediction revealed similar structural motifs of the longer and the wildtype PrfA variant, consistent to the structural motifs described by Scortti et al. [38]. However, the C-terminus of the longer PrfA variant was extended with an additional α-helix and a coiled region. It is highly likely that this structural alteration impacts the functionality of PrfA. From the current point of our investigation, we can only speculate that this structural alteration could either prevent the formation of the PrfA homodimer, which is required to activate transcription by binding to a 14 bp palindromic promoter sequence (PrfA box), or allow the formation of the homodimer but substantially decrease the promoter affinity [39]. From an evolutionary perspective, the phenomenon of losing virulence characteristics can be explained by a fitness trade-off where the success of *L. monocytogenes* colonizing a new niche like food or food processing environments is driven by the cost of losing its ability to successfully invade host cells in favor of reasonable energy consumption. Since this PrfA variant occurs at a very low frequency and is not distributed across all food-associated ST155 strains, we suggest that this genetic variation was acquired occasionally. Besides the longer *prfA* variant, we could not detect any association between the virulence potential of the 20 selected ST155 strains and the alteration or mutation of virulence factors.

The genome-wide analysis of 69 stress-associated genes revealed a consistent and conserved distribution across the 130 *L. monocytogenes* ST155 genomes. All of the strains harbored SSI-1, which confers tolerance towards acidic, osmotic, gastric, and bile stress and enhancing both the survival in food as well as the pathogenicity in the human host [8]. SSI-1 provides a broader spectrum of adaptation compared to SSI-2, which confers increased survival during alkaline and oxidative stress conditions relevant in food processing environments [15]. Compared to EGD-e, we detected a longer variant of the ABC transporter substrate-binding protein OppA, which is involved in growth under low temperatures and intracellular survival in macrophages. This longer variant is also present in *L. monocytogenes* strain LO28 [40]. The effect of the different variants on the function of OppA is unknown.

Here, we provide evidence for the presence of highly conserved plasmids in ST155 *L. monocytogenes* strains sharing more than 99% ANI - except for the divergent plasmid from strain BCW_3928. This high level of conservation is in line with previous findings for plasmids in *L. monocytogenes* ST204, ST121, and other ST155 strains [41, 42]. The presence of a group 1 plasmid in strain BCW_3928 in this study was somewhat surprising, as another recent study revealed that all ST155 plasmids in *L. monocytogenes* strains from France were also 90 kbp group 2 plasmids [42]. Our study and the studies mentioned above suggest that highly conserved plasmids can be found in different *L. monocytogenes* strains from various sources worldwide. The plasmid-containing strains in this study were isolated between 1957 and 2018 from Austria, Canada, Italy, and the USA. We show the presence of plasmids in 34.6% of all ST155 strains included in this study. This overall abundance of plasmids is slightly lower than other studies that reported plasmids in 43% of ST155 strains and in 48 to 55% of the strains of various STs [42–44]. Our knowledge about the function of plasmids in *L. monocytogenes* is still highly limited. However, some recent studies have either demonstrated a contribution of plasmids to *L. monocytogenes* stress response [25, 26] or revealed upregulation of plasmid genes during stress conditions [45, 46]. These data suggest that plasmids may be beneficial for the stress response or survival of *L. monocytogenes* ST155 strains in food or food processing environments. We hypothesize that strains harboring a plasmid have a higher tolerance to QACs and cadmium due to the presence of *brcABC* and *cadA*, which requires experimental validation in future studies.

## Conclusions

*L. monocytogenes* ST155 strains have been isolated from food, food processing environments and human listeriosis cases. The present study contributes to an enhanced understanding of the genomic features and virulence characteristics of ST155 strains. Six of 20 tested strains showed an impaired virulence potential in Caco2 and HEPG2 cells, which could be traced back to a longer variant of PrfA and the concomitantly lower or absent expression of the virulence factor genes *inlA*, *inlB*, *hly, actA,* and *prfA* itself. All 130 ST155 genomes were very similar in genes associated with virulence and stress resistance. The plasmids harbored by some of the strains also showed a high degree of conservation. Overall, our findings regarding the phylogenetic relationship and the conserved characteristics of virulence and stress-associated factors as well as plasmids in ST155 strains independent of the source of isolation genetically support the ability of this sequence type to equally adapt to conditions in foods and food processing environments as well as to the human host.

## Methods

### *Listeria monocytogenes* strains

Twenty ST155 *L. monocytogenes* strains, including eleven food isolates and nine human clinical isolates, were used for in vitro virulence assays (Table 1). The strains from the Unit of Food Microbiology, University of Veterinary Medicine Vienna were isolated in frame of two previous projects [21, 47]. Additionally, we screened 477 strains of the internal strain collection by PCR targeting the CC155-specific gene *LMLG_RS02400* according to Chenal-Francisque et al. [48] and subsequently determined the MLST. Nine strains were provided by the Austrian National Reference Center for *Listeria*, Austrian Ageny for Health and Food Safety (AGES). Additionally, a ScottA variant (ST2, CC2) harboring 193 SNP-differences to ScottA (ST290, CC2) from the NCBI genome database was included as a reference strain.

### In vitro virulence assays

For the in vitro virulence assays, human intestinal epithelial Caco2 (ATCC® HTB­37™) and hepatocytic HEPG2 cells (ATCC® HB­8065™) were cultivated in Eagle’s minimum essential medium (MEM; Thermo Scientific) containing 2 mM L-glutamine, 10% fetal bovine serum (FBS), 100 units/ml penicillin, 100 mg/ml streptomycin sulphate and 0.25 mg/ml amphotericin B as well as 1% non-essential amino acids at 37 °C in a humidified atmosphere (95% relative humidity) containing 5% CO_2_. Prior to infection, confluent cell monolayers (175 cm^2^) were washed twice with PBS, harvested by trypsinization (Trypsin-EDTA 0.25%) and seeded in 24-well plates in MEM containing 2 mM L-glutamine, 10% FBS, 100 units/ml penicillin, 100 mg/ml streptomycin sulphate and 0.25 mg/ml amphotericin B as well as 1% non-essential amino acids. The medium was changed 16 h before infection to MEM containing 2 mM L-glutamine and 10% fetal bovine serum without antibiotics. Single colonies of *L. monocytogenes* were inoculated in brain heart infusion supplemented with yeast (BHI-Y). Bacterial strains were cultivated for 16 h at 37 °C shaking at 150 rpm. The bacterial cultures were adjusted to an OD_600_ 0.2 in 10 ml BHI-Y and incubated for 2 h at 37 °C without shaking to reach exponential growth phase as confirmed by OD_600_ measurements.

Cell monolayers were infected with *L. monocytogenes* at a multiplicity of infection of 25 for 1 h at 37 °C. Colony-forming units (CFU) of the inocula were determined by plating serial dilutions on tryptic soy agar supplemented with yeast (TSA-Y). The infected cell monolayers were subsequently washed with PBS and incubated in MEM containing 10% FBS and 100 μg/ml gentamicin for 45 min (invasion) and 4 h (intracellular proliferation), respectively. After the incubation periods, the cells were lysed using 1 ml 0.1% Triton X-100 (Merck). CFU were determined by plating serial dilutions on TSA-Y. The invasion efficiencies were calculated as CFU recovered after 45 min of incubation in gentamicin-containing medium divided by the mean CFU of the inoculum. The intracellular growth coefficient was determined as follows: IGC = (intracellular bacteria _4 h_ – intracellular bacteria _45 min_)/intracellular bacteria _45 min_. Each experiment was performed in triplicates and repeated at least three times.

### Whole genome sequencing

DNA was isolated from bacterial cultures using the MagAttract HMW DNA Kit (Qiagen, Hilden, Germany) using the protocol for Gram-positive bacteria according to the instructions of the manufacturer. The amount of input DNA was quantified on a Lunatic instrument (Unchained Labs, Pleasanton, CA, USA). Sequencing libraries were prepared using Nextera XT chemistry (Illumina Inc., San Diego, CA, USA) for a 300-bp paired-end sequencing run on an Illumina MiSeq sequencer. Samples were sequenced to aim for minimum coverage of 80-fold using Illumina’s recommended standard protocols. The resulting FASTQ files were first quality trimmed and then de novo assembled using SPAdes version 3.9.0. Contigs were filtered for a minimum coverage of 5× and a minimum length of 200 bp using SeqSphere+ software v6.0.0 (Ridom, Münster, Germany).

### Retrieval of *L. monocytogenes* ST155 strains from genome databases, MLST determination, and sequence analysis

The CC155-specific gene *LMLG_RS02400* was used to retrieve strains of this clonal complex [48] from GenBank by BlastN [49] using the NCBI whole-genome shotgun contigs database. Determination of the ST of the strains was performed using the MLST tool available on the Center for Genomic Epidemiology website (https://cge.cbs.dtu.dk/services/MLST) using the *L. monocytogenes* scheme [50]. In order to quickly investigate the genomic features of the obtained *L. monocytogenes* ST155 dataset, the genomes were submitted to TORMES v1.1 [51], a pipeline for whole bacterial genome sequencing data investigation [51]. Genome size, GC content and N_50_ values of each genome were calculated by using QUAST [52], contained in the TORMES pipeline (Table S1). The chromosomes and plasmids of selected strains were further annotated and analyzed using the Patric and RAST webservers [53, 54]. Sequence similarity searches were performed with NCBI Blast included in Patric and RAST. The alignment of plasmids was performed with MAUVE [27].

### Analysis of frequency and characteristics of virulence and stress-associated genes

FASTA files were retrieved from the NCBI gene database [55] available at https://www.ncbi.nlm.nih.gov/gene/ (cited: 2020-04-23) and processed by the makeblastdb-executable (BLAST+ Version 2.9.0) [49] to generate local BLAST databases of the identified virulence genes (Table S3) and the stress-associated genes (Table S4). Using a custom created python-script, the whole genome sequences of the ST155 strains were compared against the newly created databases of virulence and stress-associated genes using the BlastN-program. As analysis parameters, a maximum number of 1000 target sequences, a maximum of one hit per target sequence, and an E-value of 0.01 were used.

### Phylogenetic analysis

Roary v3.13.0 [55] was used to perform a pangenome analysis based on presence/absence of genes between the 130 *L. monocytogenes* genomes’ dataset and an alignment of genes identified as core genes (genes present in more than 99% of the genomes from the dataset). The resulting alignment was used to perform a phylogenetic tree by using FastTree v2.1.10 [56]. These two steps were automatically performed by the TORMES pipeline. The resulting newick files were then imported to R environment (v.3.6.1, https://www.r-project.org) for tree visualizations using *ggtree* v2.0.4 [57] and *treeio* v1.10.0 [58] packages. Mid-point rooting of the phylogenetic tree was performed by using *phangorn* v2.5.5 [59].

Roary was also used to identify the core genes between genomes of CDL65, P06_14, Ro05, Ro07, Ro09 and Ro15 (all harboring the long PrfA variant). The resulting core genes nucleotide alignment file was used to calculate core genes SNP distances by using snp-dists v0.7.0 (T. Seemann et al., https://github.com/tseemann/snp-dists).

### Isolation of mRNA and transcription into cDNA

Single colonies of *L. monocytogenes* were inoculated in BHI-Y in triplicates and cultivated for 16 h at 37 °C shaking at 150 rpm. Each bacterial culture was then adjusted to an OD_600_ 0.2 in 10 ml BHI-Y. Cultures were incubated for 2 h at 37 °C without shaking and were then centrifuged at 3220 g for 10 min at room temperature. The pellets were resuspended in 1 ml TRIzol Reagent (Thermo Scientific). Cells were disrupted using bead-beating in Lysing Matrix A tubes (MP Biomedicals) with a FastPrep FP120 instrument (MP Biomedicals) and the following parameters: three times 45 s at speed 4.5 at 4 °C. RNA was isolated by chloroform phase separation and isopropanol RNA precipitation. The RNA pellet was washed with 75% ethanol, dried, and dissolved in 50 μl RNase free water. The RNA amount was measured using Qubit® 2.0 Fluorometer (Invitrogen). The remaining DNA was digested using the Turbo DNA-free Kit (Life Technologies, Agilent) according to the manufacturer instructions. A PCR targeting the *16S rRNA* gene was performed using DreamTaq DNA Polymerase Mix (Thermo Scientific) to confirm the absence of DNA. PCR cycling conditions were as follows: initial denaturation at 95 °C for 5 min; 30 cycles of denaturation at 95 °C for 15 s, annealing at 60 °C for 30 s, elongation at 72 °C for 30 s; final elongation at 72 °C for 2 min; hold at 4 °C. RNA amounts between 200 ng and 300 ng were used for cDNA synthesis using the RevertAid H Minus First Strand cDNA Synthesis Kit (Thermo Scientific) according to the manufacturer protocol.

### Quantitative RT-PCR

Primers targeting the *L. monocytogenes 16S rRNA*, *inlA*, *inlB*, *hly, actA,* and *prfA* genes were designed using Primer3 (v.0.4.0) (Table S6). PCR conditions were applied based on Platinum Taq DNA Polymerase PCR set-up instructions: 1 x PCR Buffer, 250 nM primer forward and reverse, 3.75 mM MgCl_2_, 1.5 mM dNTP-Mix, 2 U Platinum Taq DNA polymerase (Life Technologies) and 1 μM EvaGreen (Jena Bioscience) in a final volume of 20 μl. Five microliter of cDNA template were used. Cycling conditions for *16S rRNA* were as follows: initial denaturation at 94 °C for 2 min, 45 cycles of denaturation at 94 °C for 30 s and annealing at 60 °C for 1 min, 3 cycles of denaturation at 95 °C for 1 min and annealing at 60 °C for 3 min and final elongation at 95 °C for 30 s. Cycling conditions for *inlA*, *inlB*, *hly*, *actA,* and *prfA* were as follows: initial denaturation at 94 °C for 2 min, 45 cycles of denaturation at 94 °C for 30 s and annealing at 60 °C for 1 min, 3 cycles of denaturation at 95 °C for 1 min and annealing at 60 °C for 30 s and final elongation at 95 °C for 30 s. All qRT-PCR reactions were performed using a Mx3000PTM cycler (Stratagene®). Subsequently, a dissociation curve was established (55–95 °C, 0.1 °C/second). A dilution series of genomic QOC1 wildtype DNA (1–10^− 6^ ng/μl) was used to determine the primer efficiency (1.79–1.99 for *16S rRNA*, 1.90–1.99 for *inlA*, 2.01–2.37 for *inlB*, 1.94–2.08 for *actA*, 1.90–1.96 for *hly*, and 1.90–2.22 for *prfA*). Data were analyzed using Mx3000PTM MxPro software (Stratagene®). Each sample was measured in duplicates, and relative quantification was performed using the comparative Ct method. Values, given as x-fold of L23–13, were normalized to *16S rRNA* as an internal reference (60). Mean values ± standard deviation (SD) of three biological replicates performed in triplicates and measured in duplicates were calculated.

### Determination of structural protein domains and protein structure prediction

The structural protein domains of InlA, Auto and PrfA variants in selected *L. monocytogenes* ST155 strains and their EGD-e homologs were determined using the MotifFinder tool available on the GenomeNet website of the Kyoto University Bioinformatics Center (https://www.genome.jp/tools/motif/).

The protein structure of the wildtype PrfA variant in EGD-e and the longer PrfA variant in the six *L. monocytogenes* ST155 strains was predicted using the I-TASSER server for protein structure and function predictions [61].

### Statistical analysis

Statistical analysis was performed using SPSS.20 software (SPSS Inc., Chicago USA). The mean values and standard deviations (SD) were calculated. Brown Forsythe and Welch tests were applied to confirm variance homogeneity. Games-Howell posthoc test (variance heterogeneity) was used to determine significant differences between the invasion efficiencies, IGCs and gene expression levels of the selected ST155 strains. *p*-values < 0.05 were considered to be statistically significant.

## Supplementary Information

**Additional file 1. Table S1.**

**Additional file 2. Table S2.**

**Additional file 3. Table S3.**

**Additional file 4. Figure S1-7.**

**Additional file 5. Table S4.**

**Additional file 6. Table S5.**

**Additional file 7. Table S6.**

**Additional file 8. Table S7.**

## Data Availability

This Whole Genome Shotgun project including sequencing data and assembled genomes has been deposited at DDBJ/ENA/GenBank under the bioproject accession number PRJNA647251 and the accession JACGSS000000000 to JACGTB000000000 and JACLAK000000000.

## Abbreviations

CC: Clonal complex
IGC: Intracellular growth coefficient
LIPI: *Listeria* Pathogenicity Island
*L. monocytogenes*: *Listeria monocytogenes*
QAC: Quaternary ammonium compounds
qRT-PCR: quantitative real time polymerase chain reaction
SD: Standard deviation
SNP: Single nucleotide polymorphism
SSI: Stress survival islet
ST: Sequence type
