# Supplementary material for: Virulence characterization and comparative genomics of Listeria monocytogenes sequence type 155 strains
Source: BMC Genomics. 2020 Nov 30;21:847. doi: 10.1186/s12864-020-07263-w (PMC7708227; doi:10.1186/s12864-020-07263-w)
Supplement: Supplementary file 2 — Additional file 2. Table S2. [file 12864_2020_7263_MOESM2_ESM.pdf]

Table S2: Core gene SNP distances between strains harboring the longer PrfA variant

	CDL65	P06_14	Ro05	Ro07	Ro09	Ro15
CDL65	0	173	193	229	177	368
P06_14	173	0	176	212	130	350
Ro05	193	176	0	162	144	303
Ro07	229	212	162	0	180	290
Ro09	177	130	144	180	0	313
Ro15	368	350	303	290	313	0
