# Supplementary material for: Virulence characterization and comparative genomics of Listeria monocytogenes sequence type 155 strains
Source: BMC Genomics. 2020 Nov 30;21:847. doi: 10.1186/s12864-020-07263-w (PMC7708227; doi:10.1186/s12864-020-07263-w)

Figure S1. Distribution and variability of virulence factors in 130 *L. monocytogenes* ST155 strains.

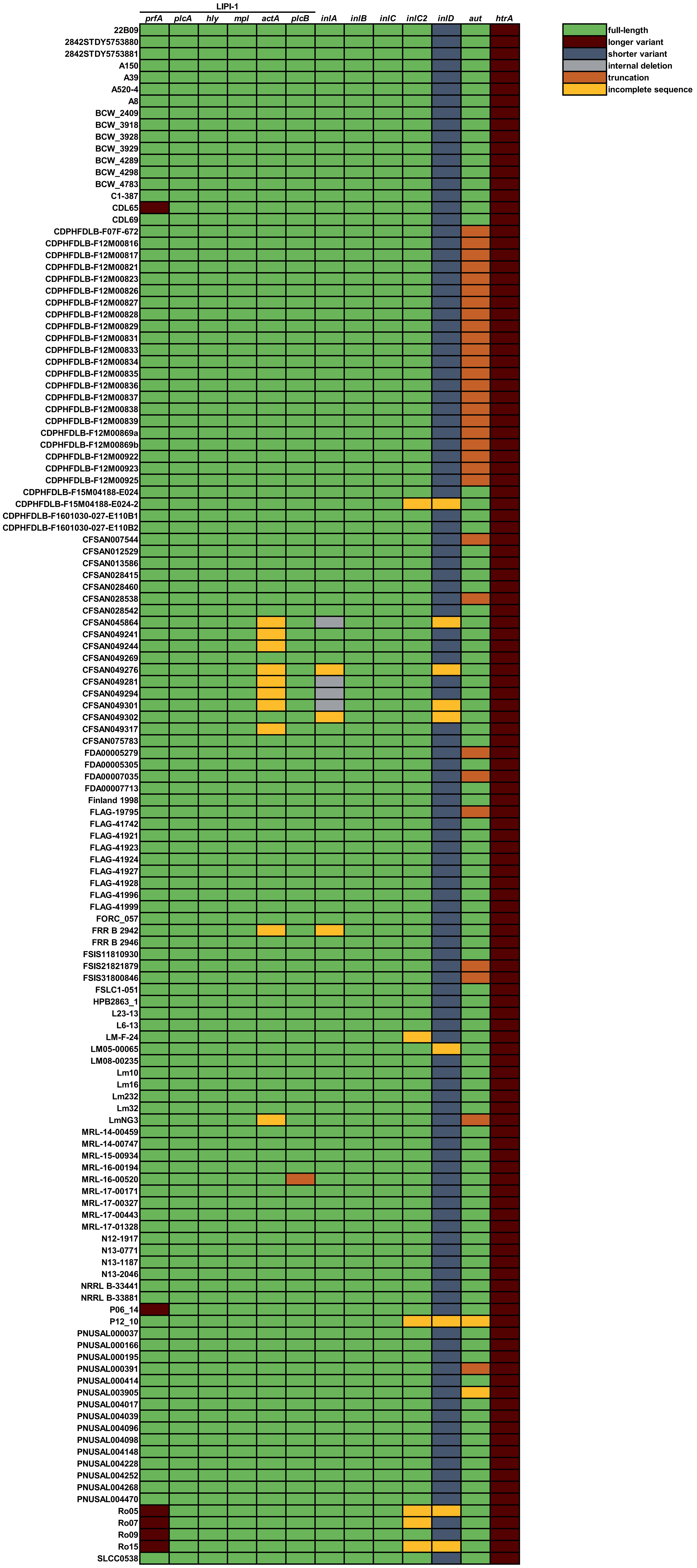

Figure S2A. Alignment of *inlA* nucleotide sequences

```
ScottA_ST2      1  GTGAGAAGAAAACGATATGTATGGTTGAAAAGTATACTAGTAGCAATATTAGTATTTGGCAGTGGAGTAT
EGD-e_ST35      1  GTGAGAAAAAAACGATATGTATGGTTGAAAAGTATACTAGTAGCAATATTAGTATTTGGCAGCGGAGTAT
10403S_ST85     1  GTGAGAAAAAAACGATATGTATGGTTGAAAAGTATACTAGTAGCAATATTAGTATTTGGCAGTGGAGTAT
F2365_ST1       1  GTGAGAAGAAAACGATATGTATGGTTGAAAAGTATACTAGTAGCAATATTAGTATTTGGCAGTGGAGTAT
L6-13           1  GTGAGAAAAAAACGATATGTATGGTTGAAAAGTATACTAGTAGCAATATTAGTATTTGGCAGTGGAGTAT
MRL-14-00459    1  GTGAGAAAAAAACGATATGTATGGTTGAAAAGTATACTAGTAGCAATATTAGTATTTGGCAGTGGAGTAT
CFSAN045864     1  GTGAGAAAAAAACGATATGTATGGTTGAAAAGTATACTAGTAGCAATATTAGTATTTGGCAGTGGAGTAT
CFSAN049281     1  GTGAGAAAAAAACGATATGTATGGTTGAAAAGTATACTAGTAGCAATATTAGTATTTGGCAGTGGAGTAT
CFSAN049294     1  GTGAGAAAAAAACGATATGTATGGTTGAAAAGTATACTAGTAGCAATATTAGTATTTGGCAGTGGAGTAT
CFSAN049301     1  GTGAGAAAAAAACGATATGTATGGTTGAAAAGTATACTAGTAGCAATATTAGTATTTGGCAGTGGAGTAT

ScottA_ST2     71  GGATTAACACGAGTAACGGGACAAATGCTCAGGCAGCTACAATTACACAAGATACTCCTATTAATCAGAT
EGD-e_ST35     71  GGATTAACACGAGTAACGGGACAAATGCTCAGGCAGCTACAATTACACAAGATACTCCTATTAATCAGAT
10403S_ST85    71  GGATTAACACGAGCAACGGGACAAATGCTCAGGCAGCTACAATTACACAAGATACTCCTATTAATCAGAT
F2365_ST1      71  GGATTAACACGAGTAACGGGACAAATGCTCAGGCAGCTACAATTACACAAGATACTCCTATTAATCAGAT
L6-13          71  GGATTAACACGAGCAACGGGACAAATGCTCAGGCAGCTACAATTACACAAGATACTCCTATTAATCAGAT
MRL-14-00459   71  GGATTAACACGAGCAACGGGACAAATGCTCAGGCAGCTACAATTACACAAGATACTCCTATTAATCAGAT
CFSAN045864    71  GGATTAACACGAGCAACGGGACAAATGCTCAGGCAGCTACAATTACACAAGATACTCCTATTAATCAGAT
CFSAN049281    71  GGATTAACACGAGCAACGGGACAAATGCTCAGGCAGCTACAATTACACAAGATACTCCTATTAATCAGAT
CFSAN049294    71  GGATTAACACGAGCAACGGGACAAATGCTCAGGCAGCTACAATTACACAAGATACTCCTATTAATCAGAT
CFSAN049301    71  GGATTAACACGAGCAACGGGACAAATGCTCAGGCAGCTACAATTACACAAGATACTCCTATTAATCAGAT

ScottA_ST2    141  TTTTACAGATACAGCTCTAGCGGAAAAAATGAAGACGGTCTTAGGAAAAACGAATGTAACAGACACGGTC
EGD-e_ST35    141  TTTTACAGATACAGCTCTAGCGGAAAAAATGAAGACGGTCTTAGGAAAAACGAATGTAACAGACACGGTC
10403S_ST85   141  TTTTACAGACGCAGCTCTAGCGGAAAAAATGAAGACGGTCTTAGGAAAAACGAATGTAACAGACACGGTC
F2365_ST1     141  TTTTACAGATACAGCTCTAGCGGAAAAAATGAAGACGGTCTTAGGAAAAACGAATGTAACAGACACGGTC
L6-13         141  TTTTACAGACGCAGCTCTAGCGGAAAAAATGAAGACGGTCTTAGGAAAAACGAATGTAACAGACACGGTC
MRL-14-00459  141  TTTTACAGACGCAGCTCTAGCGGAAAAAATGAAGACGGTCTTAGGAAAAACGAATGTAACAGACACGGTC
CFSAN045864   141  TTTTACAGACGCAGCTCTAGCGGAAAAAATGAAGACGGTCTTAGGAAAAACGAATGTAACAGACACGGTC
CFSAN049281   141  TTTTACAGACGCAGCTCTAGCGGAAAAAATGAAGACGGTCTTAGGAAAAACGAATGTAACAGACACGGTC
CFSAN049294   141  TTTTACAGACGCAGCTCTAGCGGAAAAAATGAAGACGGTCTTAGGAAAAACGAATGTAACAGACACGGTC
CFSAN049301   141  TTTTACAGACGCAGCTCTAGCGGAAAAAATGAAGACGGTCTTAGGAAAAACGAATGTAACAGACACGGTC

ScottA_ST2    211  TCGCAAACAGATCTAGACCAAGTTACGACGCTTCAGGCGGATAGATTAGGGATAAAATCTATCGATGGAT
EGD-e_ST35    211  TCACAAACAGATCTAGACCAAGTTACAACGCTTCAGGCGGATAGATTAGGGATAAAATCTATCGATGGAG
10403S_ST85   211  TCGCAAACAGATCTAGACCAAGTTACAACGCTTCAGGCGGATAGATTAGGGATAAAATCTATCGATGGAT
F2365_ST1     211  TCGCAAACAGATCTAGACCAAGTTACGACGCTTCAGGCGGATAGATTAGGGATAAAATCTATCGATGGAT
L6-13         211  TCACAAACAGATCTAGACCAAGTTACGACGCTTCAGGCGGATAGGTTAGGGATAAAATCTATCGATGGAG
MRL-14-00459  211  TCACAAACAGATCTAGACCAAGTTACGACGCTTCAGGCGGATAGGTTAGGGATAAAATCTATCGATGGAG
CFSAN045864   211  TCACAAACAGATCTAGACCAAGTTACGACGCTTCAGGCGGATAGGTTAGGGATAAAATCTATCGATGGAG
CFSAN049281   211  TCACAAACAGATCTAGACCAAGTTACGACGCTTCAGGCGGATAGGTTAGGGATAAAATCTATCGATGGAG
CFSAN049294   211  TCACAAACAGATCTAGACCAAGTTACGACGCTTCAGGCGGATAGGTTAGGGATAAAATCTATCGATGGAG
CFSAN049301   211  TCACAAACAGATCTAGACCAAGTTACGACGCTTCAGGCGGATAGGTTAGGGATAAAATCTATCGATGGAG

ScottA_ST2    281  TGGAATACTTGAACAATTTAACACAAATAAATTTCAGCAATAATCAACTTACGGACATAACTCCACTTAA
EGD-e_ST35    281  TGGAATACTTGAACAATTTAACACAAATAAATTTCAGCAATAATCAACTTACGGACATAACGCCACTTAA
10403S_ST85   281  TGGAATACTTGAACAATTTAACACAAATAAATTTCAGCAATAATCAACTTACGGATATAACGCCACTTAA
F2365_ST1     281  TGGAATACTTGAACAATTTAACACAAATAAATTTCAGCAATAATCAACTTACGGACATAACTCCACTTAA
L6-13         281  TGGAATACTTGAACAATTTAACACAAATAAATTTCAGCAATAATCAACTTACGGACATAACGCCACTTAA
MRL-14-00459  281  TGGAATACTTGAACAATTTAACACAAATAAATTTCAGCAATAATCAACTTACGGACATAACGCCACTTAA
CFSAN045864   281  TGGAATACTTGAACAATTTAACACAAATAAATTTCAGCAATAATCAACTTACGGACATAACGCCACTTAA
CFSAN049281   281  TGGAATACTTGAACAATTTAACACAAATAAATTTCAGCAATAATCAACTTACGGACATAACGCCACTTAA
CFSAN049294   281  TGGAATACTTGAACAATTTAACACAAATAAATTTCAGCAATAATCAACTTACGGACATAACGCCACTTAA
CFSAN049301   281  TGGAATACTTGAACAATTTAACACAAATAAATTTCAGCAATAATCAACTTACGGACATAACGCCACTTAA

ScottA_ST2    351  AGATTTAACTAAGTTAGTTGATATTTTGATGAATAATAATCAAATAGCAGATATAACTCCGCTAGCTAAT
EGD-e_ST35    351  AAATTTAACTAAGTTAGTTGATATTTTGATGAATAATAATCAAATAGCAGATATAACTCCGCTAGCTAAT
10403S_ST85   351  AGATTTAACTAAGTTAGTTGATATTTTGATGAATAATAATCAAATAGCAGATATAACTCCGCTAGCTAAT
F2365_ST1     351  AGATTTAACTAAGTTAGTTGATATTTTGATGAATAATAATCAAATAGCAGATATAACTCCGCTAGCTAAT
L6-13         351  AAATTTAACTAAGTTAGTTGATATTTTGATGAATAATAATCAAATAGCAGATATAACTCCGCTAGCTAAT
MRL-14-00459  351  AAATTTAACTAAGTTAGTTGATATTTTGATGAATAATAATCAAATAGCAGATATAACTCCGCTAGCTAAT
CFSAN045864   351  AAATTTAACTAAGTTAGTTGATATTTTGATGAATAATAATCAAATAGCAGATATAACTCCGCTAGCTAAT
CFSAN049281   351  AAATTTAACTAAGTTAGTTGATATTTTGATGAATAATAATCAAATAGCAGATATAACTCCGCTAGCTAAT
CFSAN049294   351  AAATTTAACTAAGTTAGTTGATATTTTGATGAATAATAATCAAATAGCAGATATAACTCCGCTAGCTAAT
CFSAN049301   351  AAATTTAACTAAGTTAGTTGATATTTTGATGAATAATAATCAAATAGCAGATATAACTCCGCTAGCTAAT

ScottA_ST2    421  TTGTCGAATCTAACTGGTTTGACTTTGTTCAACAATCAGATAACGGATATAGACCCGCTTAAAAATCTAA
EGD-e_ST35    421  TTGACGAATCTAACTGGTTTGACTTTGTTCAACAATCAGATAACGGATATAGACCCGCTTAAAAATCTAA
10403S_ST85   421  TTGACGAATCTAACTGGTTTGACTTTGTTCAACAATCAGATAACAGATATAGACCCGCTTAAAAATCTAA
F2365_ST1     421  TTGTCGAATCTAACTGGTTTGACTTTGTTCAACAATCAGATAACGGATATAGACCCGCTTAAAAATCTAA
L6-13         421  TTGACGAATCTAACTGGTTTGACTTTGTTCAACAATCAGATAACGGATCTAGACCCGCTTAAAAATCTAA
MRL-14-00459  421  TTGACGAATCTAACTGGTTTGACTTTGTTCAACAATCAGATAACGGATCTAGACCCGCTTAAAAATCTAA
CFSAN045864   421  TTGACGAATCTAACTGGTTTGACTTTGTTCAACAATCAGATAACGGATCTAGACCCGCTTAAAAATCTAA
CFSAN049281   421  TTGACGAATCTAACTGGTTTGACTTTGTTCAACAATCAGATAACGGATCTAGACCCGCTTAAAAATCTAA
```

```
CFSAN049294   421 TTGACGAATCTAACTGGTTTGACTTTGTTCAACAATCAGATAACGGATCTAGACCCGCTTAAAAATCTAA
CFSAN049301   421 TTGACGAATCTAACTGGTTTGACTTTGTTCAACAATCAGATAACGGATCTAGACCCGCTTAAAAATCTAA

ScottA_ST2    491 CAAATTTAAATCGGCTAGAACTATCTAGTAACACGATTAGTGATATTAGTGCGCTTTCAGGTTTAACTAG
EGD-e_ST35    491 CAAATTTAAATCGGCTAGAACTATCCAGTAACACGATTAGTGATATTAGTGCGCTTTCAGGTTTAACTAG
10403S_ST85   491 CAAATTTAAATCGGCTAGAACTATCTAGTAACACGATTAGTGATATTAGTGCGCTTTCAGGTTTAACTAA
F2365_ST1     491 CAAATTTAAATCGGCTAGAACTATCTAGTAACACGATTAGTGATATTAGTGCGCTTTCAGGTTTAACTAG
L6-13         491 CAAATTTAAATCGGCTAGAACTATCTAGTAACACGATTAGTGATATTAGTGCGCTTTCAGGTTTAACTAG
MRL-14-00459  491 CAAATTTAAATCGGCTAGAACTATCTAGTAACACGATTAGTGATATTAGTGCGCTTTCAGGTTTAACTAG
CFSAN045864   491 CAAATTTAAATCGGCTAGAACTATCTAGTAACACGATTAGTGATATTAGTGCGCTTTCAGGTTTAACTAG
CFSAN049281   491 CAAATTTAAATCGGCTAGAACTATCTAGTAACACGATTAGTGATATTAGTGCGCTTTCAGGTTTAACTAG
CFSAN049294   491 CAAATTTAAATCGGCTAGAACTATCTAGTAACACGATTAGTGATATTAGTGCGCTTTCAGGTTTAACTAG
CFSAN049301   491 CAAATTTAAATCGGCTAGAACTATCTAGTAACACGATTAGTGATATTAGTGCGCTTTCAGGTTTAACTAG

ScottA_ST2    561 TCTACAGCAATTATCTTTTGGTAATCAAGTGACAGATTTAAAACCATTAGCTAATTTAACAACACTAGAA
EGD-e_ST35    561 TCTACAGCAATTATCTTTTGGTAATCAAGTGACAGATTTAAAACCATTAGCTAATTTAACAACACTAGAA
10403S_ST85   561 TCTACAGCAATTATCTTTTGGTAATCAAGTGACAGATTTAAAACCATTAGCTAATTTAACAACACTAGAA
F2365_ST1     561 TCTACAGCAATTATCTTTTGGTAATCAAGTGACAGATTTAAAACCATTAGCTAATTTAACAACACTAGAA
L6-13         561 TCTACAGCAATTATCTTTTGGTAATCAAGTGACAGATTTAAAACCATTAGCTAATTTAACGACACTAGAA
MRL-14-00459  561 TCTACAGCAATTATCTTTTGGTAATCAAGTGACAGATTTAAAACCATTAGCTAATTTAACGACACTAGAA
CFSAN045864   561 TCTACAGCAATTATCTTTTGGTAATCAAGTGACAGATTTAAAACCATTAGCTAATTTAACGACACTAGAA
CFSAN049281   561 TCTACAGCAATTATCTTTTGGTAATCAAGTGACAGATTTAAAACCATTAGCTAATTTAACGACACTAGAA
CFSAN049294   561 TCTACAGCAATTATCTTTTGGTAATCAAGTGACAGATTTAAAACCATTAGCTAATTTAACGACACTAGAA
CFSAN049301   561 TCTACAGCAATTATCTTTTGGTAATCAAGTGACAGATTTAAAACCATTAGCTAATTTAACGACACTAGAA

ScottA_ST2    631 CGACTAGATATTTCAAGTAATAAGGTGTCGGATATTAGTGTTCTGGCTAAATTAACCAATTTAGAAAGTC
EGD-e_ST35    631 CGACTAGATATTTCAAGTAATAAGGTGTCGGATATTAGTGTTCTGGCTAAATTAACCAATTTAGAAAGTC
10403S_ST85   631 CGACTAGATATTTCAAGTAATAAGGTGTCAGATATTAGTGTTCTGGCTAAATTAACCAATTTAGAAAGTC
F2365_ST1     631 CGACTAGATATTTCAAGTAATAAGGTGTCGGATATTAGTGTTCTGGCTAAATTAACCAATTTAGAAAGTC
L6-13         631 CGACTAGATATTTCAAGTAATAAGGTGTCGGATATTAGTGTTCTGGCTAAATTAACCAATTTAGAAAGTC
MRL-14-00459  631 CGACTAGATATTTCAAGTAATAAGGTGTCGGATATTAGTGTTCTGGCTAAATTAACCAATTTAGAAAGTC
CFSAN045864   631 CGACTAGATATTTCAAGTAATAAGGTGTCGGATATTAGTGTTCTGGCTAAATTAACCAATTTAGAAAGTC
CFSAN049281   631 CGACTAGATATTTCAAGTAATAAGGTGTCGGATATTAGTGTTCTGGCTAAATTAACCAATTTAGAAAGTC
CFSAN049294   631 CGACTAGATATTTCAAGTAATAAGGTGTCGGATATTAGTGTTCTGGCTAAATTAACCAATTTAGAAAGTC
CFSAN049301   631 CGACTAGATATTTCAAGTAATAAGGTGTCGGATATTAGTGTTCTGGCTAAATTAACCAATTTAGAAAGTC

ScottA_ST2    701 TTATCGCTACTAACAACCAAATAAGTGATATAACTCCACTTGGGATTTTAACAAATTTGGACGAATTATC
EGD-e_ST35    701 TTATCGCTACTAACAACCAAATAAGTGATATAACTCCACTTGGGATTTTAACAAATTTGGACGAATTATC
10403S_ST85   701 TTATCGCTACTAACAACCAAATAAGTGATATAACTCCACTTGGGATTTTAACAAATTTGGACGAATTATC
F2365_ST1     701 TTATCGCTACTAACAACCAAATAAGTGATATAACTCCACTTGGGATTTTAACAAATTTGGACGAATTATC
L6-13         701 TTATCGCTACTAACAACCAAATAAGTGATATAACTCCACTTGGGATTTTAACAAATTTGGACGAATTATC
MRL-14-00459  701 TTATCGCTACTAACAACCAAATAAGTGATATAACTCCACTTGGGATTTTAACAAATTTGGACGAATTATC
CFSAN045864   701 TTATCGCTACTAACAACCAAATAAGTGATATAACTCCACTTGGGATTTTAACAAATTTGGACGAATTATC
CFSAN049281   701 TTATCGCTACTAACAACCAAATAAGTGATATAACTCCACTTGGGATTTTAACAAATTTGGACGAATTATC
CFSAN049294   701 TTATCGCTACTAACAACCAAATAAGTGATATAACTCCACTTGGGATTTTAACAAATTTGGACGAATTATC
CFSAN049301   701 TTATCGCTACTAACAACCAAATAAGTGATATAACTCCACTTGGGATTTTAACAAATTTGGACGAATTATC

ScottA_ST2    771 CTTAAATGGTAACCAGTTAAAAGATATAGGCACATTGGCGAGTTTAACAAACCTTACAGATTTAGATTTA
EGD-e_ST35    771 CTTAAATGGTAACCAGTTAAAAGATATAGGCACATTGGCGAGTTTAACAAACCTTACAGATTTAGATTTA
10403S_ST85   771 CTTAAATGGTAACCAGTTAAAAGATATAGGCACATTGGCGAGTTTAACAAACCTTACAGATTTAGATTTA
F2365_ST1     771 CTTAAATGGTAACCAGTTAAAAGATATAGGCACATTGGCGAGTTTAACAAACCTTACAGATTTAGATTTA
L6-13         771 CTTAAATGGTAACCAGTTAAAAGATATAGGCACATTGGCGAGTTTAACAAACCTTACAGATTTAGATTTA
MRL-14-00459  771 CTTAAATGGTAACCAGTTAAAAGATATAGGCACATTGGCGAGTTTAACAAACCTTACAGATTTAGATTTA
CFSAN045864   771 CTTAAATGGTAACCAGTTAAAAGATATAGGCACATTGGCGAGTTTAACAAACCTTACAGATTTAGATTTA
CFSAN049281   771 CTTAAATGGTAACCAGTTAAAAGATATAGGCACATTGGCGAGTTTAACAAACCTTACAGATTTAGATTTA
CFSAN049294   771 CTTAAATGGTAACCAGTTAAAAGATATAGGCACATTGGCGAGTTTAACAAACCTTACAGATTTAGATTTA
CFSAN049301   771 CTTAAATGGTAACCAGTTAAAAGATATAGGCACATTGGCGAGTTTAACAAACCTTACAGATTTAGATTTA

ScottA_ST2    841 GCAAATAACCAAATTAGTAATCTAGCACCACTGTCGGGTCTAACAAAACTAACTGAGTTAAAACTGGGAG
EGD-e_ST35    841 GCAAATAACCAAATTAGTAATCTAGCACCACTGTCGGGTCTAACAAAACTAACTGAGTTAAAACTTGGAG
10403S_ST85   841 GCAAATAACCAAATTAGTAATCTAGCACCACTGTCGGGTCTAACAAAACTAACTGAGTTAAAACTTGGAG
F2365_ST1     841 GCAAATAACCAAATTAGTAATCTAGCACCACTGTCGGGTCTAACAAAACTAACTGAGTTAAAACTGGGAG
L6-13         841 GCAAATAACCAAATTAGTAATCTAGCACCACTGTCGGGTCTAACAAAACTAACTGAGTTAAAACTTGGAG
MRL-14-00459  841 GCAAATAACCAAATTAGTAATCTAGCACCACTGTCGGGTCTAACAAAACTAACTGAGTTAAAACTTGGAG
CFSAN045864   841 GCAAATAACCAAATTAGTAATCTAGCACCACTGTCGGGTCTAACAAAACTAACTGAGTTAAAACTTGGAG
CFSAN049281   841 GCAAATAACCAAATTAGTAATCTAGCACCACTGTCGGGTCTAACAAAACTAACTGAGTTAAAACTTGGAG
CFSAN049294   841 GCAAATAACCAAATTAGTAATCTAGCACCACTGTCGGGTCTAACAAAACTAACTGAGTTAAAACTTGGAG
CFSAN049301   841 GCAAATAACCAAATTAGTAATCTAGCACCACTGTCGGGTCTAACAAAACTAACTGAGTTAAAACTTGGAG

ScottA_ST2    911 CTAACCAAATAAGTAACATCAGTCCCCTAGCAGGTCTAACCGCACTCACTAACTTAGAGCTAAATGAAAA
EGD-e_ST35    911 CTAACCAAATAAGTAACATCAGTCCCCTAGCAGGTTTAACCGCACTCACTAACTTAGAGCTTAATGAAAA
10403S_ST85   911 CTAACCAAATAAGTAACATCAGTCCCCTAGCAGGTTTAACCGCACTCACTAACTTAGAGCTTAATGAAAA
F2365_ST1     911 CTAACCAAATAAGTAACATCAGTCCCCTAGCAGGTCTAACCGCACTCACTAACTTAGAGCTAAATGAAAA
L6-13         911 CTAACCAAATAAGTAACATCAGTCCCCTAGCAGGTTTAACCGCACTCACTAACTTAGAGCTTAATGAAAA
MRL-14-00459  911 CTAACCAAATAAGTAACATCAGTCCCCTAGCAGGTTTAACCGCACTCACTAACTTAGAGCTTAATGAAAA
```

```
CFSAN045864    911  CTAACCAAATAAGTAACATCAGTCCCCTAGCAGGTTTAACCGCACTCACTAACTTAGAGCTTAATGAAAA
CFSAN049281    911  CTAACCAAATAAGTAACATCAGTCCCCTAGCAGGTTTAACCGCACTCACTAACTTAGAGCTTAATGAAAA
CFSAN049294    911  CTAACCAAATAAGTAACATCAGTCCCCTAGCAGGTTTAACCGCACTCACTAACTTAGAGCTTAATGAAAA
CFSAN049301    911  CTAACCAAATAAGTAACATCAGTCCCCTAGCAGGTTTAACCGCACTCACTAACTTAGAGCTTAATGAAAA

ScottA_ST2     981  TCAGTTAGAAGATATTAGCCCAATTTCTAACCTGAAAAATCTCACATATTTAACGTTGTACTTTAATAAT
EGD-e_ST35     981  TCAGCTGGAAGATATTAGCCCAATTTCTAACCTGAAAAATCTCACATATTTAACTTTGTACTTTAATAAT
10403S_ST85    981  TCAGCTGGAAGATATTAGCCCAATTTCTAACCTGAAAAATCTCACATATTTAACGTTGTACTTTAATAAT
F2365_ST1      981  TCAGTTAGAAGATATTAGCCCAATTTCTAACCTGAAAAATCTCACATATTTAACGTTGTACTTTAATAAT
L6-13          981  TCAGCTGGAAGATATTAGCCCAATTTCTAACCTGAAAAATCTCACATATTTAACTTTGTACTTTAATAAT
MRL-14-00459   981  TCAGCTGGAAGATATTAGCCCAATTTCTAACCTGAAAAATCTCACATATTTAACTTTGTACTTTAATAAT
CFSAN045864    981  TCAGCTGGAAGATATTAGCCCAATTTCTAACCTGAAAAATCTCACATATTTAACTTTGTACTTTAATAAT
CFSAN049281    981  TCAGCTGGAAGATATTAGCCCAATTTCTAACCTGAAAAATCTCACATATTTAACTTTGTACTTTAATAAT
CFSAN049294    981  TCAGCTGGAAGATATTAGCCCAATTTCTAACCTGAAAAATCTCACATATTTAACTTTGTACTTTAATAAT
CFSAN049301    981  TCAGCTGGAAGATATTAGCCCAATTTCTAACCTGAAAAATCTCACATATTTAACTTTGTACTTTAATAAT

ScottA_ST2     1051 ATAAGTGATATAAGCCCAGTTTCTAGTTTAACAAAGCTTCAAAGATTATTTTTCTATAATAACAAGGTAA
EGD-e_ST35     1051 ATAAGTGATATAAGCCCAGTTTCTAGTTTAACAAAGCTTCAAAGATTATTTTTCTATAATAACAAGGTAA
10403S_ST85    1051 ATAAGTGATATAAGCCCAGTTTCTAGTTTAACAAAGCTTCAAAGATTATTTTTCTATAATAACAAGGTAA
F2365_ST1      1051 ATAAGTGATATAAGCCCAGTTTCTAGTTTAACAAAGCTTCAAAGATTATTTTTCTATAATAACAAGGTAA
L6-13          1051 ATAAGTGATATAAGCCCAGTTTCTAGTTTAACAAAGCTTCAAAGATTATTTTTCTATAATAACAAGGTAA
MRL-14-00459   1051 ATAAGTGATATAAGCCCAGTTTCTAGTTTAACAAAGCTTCAAAGATTATTTTTCTATAATAACAAGGTAA
CFSAN045864    1051 ATAAGTGATATAAGCCCAGTTTCTAGTTTAACAAAGCTTCAAAGATTATTTTTCTATAATAACAAGGTAA
CFSAN049281    1051 ATAAGTGATATAAGCCCAGTTTCTAGTTTAACAAAGCTTCAAAGATTATTTTTCTATAATAACAAGGTAA
CFSAN049294    1051 ATAAGTGATATAAGCCCAGTTTCTAGTTTAACAAAGCTTCAAAGATTATTTTTCTATAATAACAAGGTAA
CFSAN049301    1051 ATAAGTGATATAAGCCCAGTTTCTAGTTTAACAAAGCTTCAAAGATTATTTTTCTATAATAACAAGGTAA

ScottA_ST2     1121 GTGACGTAAGCTCACTTGCGAATTTAACCAATATTAATTGGCTTTCGGCTGGGCATAACCAAATTAGCGA
EGD-e_ST35     1121 GTGACGTAAGCTCACTTGCGAACTTAACAAATATTAATTGGCTTTCAGCTGGGCATAACCAAATTAGCGA
10403S_ST85    1121 GTGACGTAAGCTCACTTGCGAACTTAACCAATATTAATTGGCTTTCAGCTGGGCATAACCAAATTAGCGA
F2365_ST1      1121 GTGACGTAAGCTCACTTGCGAATTTAACCAATATTAATTGGCTTTCGGCTGGGCATAACCAAATTAGCGA
L6-13          1121 GTGACGTAAGCTCACTTGCGAACTTAACAAATATTAATTGGCTTTCAGCTGGGCATAACCAAATTAGCGA
MRL-14-00459   1121 GTGACGTAAGCTCACTTGCGAACTTAACAAATATTAATTGGCTTTCAGCTGGGCATAACCAAATTAGCGA
CFSAN045864    1121 GTGACGTAAGCTCACTTGCGAACTTAACAAATATTAATTGGCTTTCAGCTGGGCATAACCAAATTAGCGA
CFSAN049281    1121 GTGACGTAAGCTCACTTGCGAACTTAACAAATATTAATTGGCTTTCAGCTGGGCATAACCAAATTAGCGA
CFSAN049294    1121 GTGACGTAAGCTCACTTGCGAACTTAACAAATATTAATTGGCTTTCAGCTGGGCATAACCAAATTAGCGA
CFSAN049301    1121 GTGACGTAAGCTCACTTGCGAACTTAACAAATATTAATTGGCTTTCAGCTGGGCATAACCAAATTAGCGA

ScottA_ST2     1191 TCTTACACCATTGGCTAATTTAACAAGAATTACCCAATTAGGATTGAATGACCAAGAATGGACAAATCCA
EGD-e_ST35     1191 TCTTACACCATTGGCTAATTTAACAAGAATCACCCAACTAGGGTTGAATGATCAAGCATGGACAAATGCA
10403S_ST85    1191 TCTTACACCATTGGCTAATTTAACAAGAATCACCCAACTAGGGTTGAATGATCAAGCATGGACAAATGCA
F2365_ST1      1191 TCTTACACCATTGGCTAATTTAACAAGAATTACCCAATTAGGATTGAATGACCAAGAATGGACAAATCCA
L6-13          1191 TCTTACACCATTGGCTAATTTAACAAGAATCACCCAACTAGGGTTGAATGATCAAGCATGGACAAATGCA
MRL-14-00459   1191 TCTTACACCATTGGCTAATTTAACAAGAATCACCCAACTAGGGTTGAATGATCAAGCATGGACAAATGCA
CFSAN045864    1191 TCTTACACCATTGGCTAATTTAACAAGAATCACCCAACTAGGGTTGAATGATCAAGCATGGACAAATGCA
CFSAN049281    1191 TCTTACACCATTGGCTAATTTAACAAGAATCACCCAACTAGGGTTGAATGATCAAGCATGGACAAATGCA
CFSAN049294    1191 TCTTACACCATTGGCTAATTTAACAAGAATCACCCAACTAGGGTTGAATGATCAAGCATGGACAAATGCA
CFSAN049301    1191 TCTTACACCATTGGCTAATTTAACAAGAATCACCCAACTAGGGTTGAATGATCAAGCATGGACAAATGCA

ScottA_ST2     1261 CCAGTGAACTACAAAGTAAATGTATCCATTCCAAACACGGTGAAAAATGTGACGGGCGCTTTGATTGCAC
EGD-e_ST35     1261 CCAGTAAACTACAAAGCAAATGTATCCATTCCAAACACGGTGAAAAATGTGACTGGCGCTTTAATTGCAC
10403S_ST85    1261 CCAGTAAACTACAAAGCAAATGTATCCATTCCAAACACGGTGAAAAATGTGACTGGCGCTTTGATTGCAC
F2365_ST1      1261 CCAGTGAACTACAAAGTAAATGTATCCATTCCAAACACGGTGAAAAATGTGACGGGCGCTTTGATTGCAC
L6-13          1261 CCAGTAAACTACAAAGCAAATGTATCCATTCCAAACACGGTGAAAAATGTGACTGGCGCTTTAATTGCAC
MRL-14-00459   1261 CCAGTAAACTACAAAGCAAATGTATCCATTCCAAACACGGTGAAAAATGTGACTGGCGCTTTAATTGCAC
CFSAN045864    1261 CCAGTAAACTACAAAGCAAATGTATCCATTCCAAACACGGTGAAAAATGTGACTGGCGCTTTAATTGCAC
CFSAN049281    1261 CCAGTAAACTACAAAGCAAATGTATCCATTCCAAACACGGTGAAAAATGTGACTGGCGCTTTAATTGCAC
CFSAN049294    1261 CCAGTAAACTACAAAGCAAATGTATCCATTCCAAACACGGTGAAAAATGTGACTGGCGCTTTAATTGCAC
CFSAN049301    1261 CCAGTAAACTACAAAGCAAATGTATCCATTCCAAACACGGTGAAAAATGTGACTGGCGCTTTAATTGCAC

ScottA_ST2     1331 CAGCTACTATTAGCGATGGTGGTAGTTATGCAGAACCTGATATAACATGGAATTTACCTAGTTATACCAA
EGD-e_ST35     1331 CAGCTACTATTAGCGATGGCGGTAGTTACACAGAGCCTGATATAACATGGAACTTACCTAGTTATACAAA
10403S_ST85    1331 CTGCTACTATTAGCGATGGCGGTAGTTACGCAGAACCGGATATAACATGGAACTTACCTAGTTATACAAA
F2365_ST1      1331 CAGCTACTATTAGCGATGGTGGTAGTTATGCAGAACCTGATATAACATGGAATTTACCTAGTTATACCAA
L6-13          1331 CAGCTACTATTAGCGATGGCGGTAGTTACACAGAGCCTGATATAACATGGAACTTACCTAGTTATACAAA
MRL-14-00459   1331 CAGCTACTATTAGCGATGGCGGTAGTTACACAGAGCCTGATATAACATGGAACTTACCTAGTTATACAAA
CFSAN045864    1331 CAGCTACTATTAGCGATGGCGGTAGTTACACAGAGCCTGATATAACATGGAACTTACCTAGTTATACAAA
CFSAN049281    1331 CAGCTACTATTAGCGATGGCGGTAGTTACACAGAGCCTGATATAACATGGAACTTACCTAGTTATACAAA
CFSAN049294    1331 CAGCTACTATTAGCGATGGCGGTAGTTACACAGAGCCTGATATAACATGGAACTTACCTAGTTATACAAA
CFSAN049301    1331 CAGCTACTATTAGCGATGGCGGTAGTTACACAGAGCCTGATATAACATGGAACTTACCTAGTTATACAAA

ScottA_ST2     1401 TGAAGTAAGTTATACCTTTAACCAATCTGTCACCATTGGAAAAGGAACGACAACATTTAGTGGAACTGTG
EGD-e_ST35     1401 TGAAGTAAGCTATACCTTTAGCCAACCTGTCACTATTGGAAAAGGAACGACAACATTTAGTGGAACCGTG
10403S_ST85    1401 TGAAGTAAGCTATACCTTTAGCCAACCTGTCACTATTGGAAAAGGAACGACAACATTTAGTGGAACCGTG
F2365_ST1      1401 TGAAGTAAGTTATACCTTTAACCAATCTGTCACCATTGGAAAAGGAACGACAACATTTAGTGGAACTGTG
```

```
L6-13          1401 TGAAGTAAGCTATACCTTTAGCCAACCTGTCACTATTGGAAAAGGAACGACAACATTTAGTGGAACCGTG
MRL-14-00459   1401 TGAAGTAAGCTATACCTTTAGCCAACCTGTCACTATTGGAAAAGGAACGACAACATTTAGTGGAACCGTG
CFSAN045864    1401 TGAAGTAAGCTATACCTTTAGCCAACCTGTCACTATTGGAAAAGGAACGACAACATTTAGTGGAACCGTG
CFSAN049281    1401 TGAAGTAAGCTATACCTTTAGCCAACCTGTCACTATTGGAAAAGGAACGACAACATTTAGTGGAACCGTG
CFSAN049294    1401 TGAAGTAAGCTATACCTTTAGCCAACCTGTCACTATTGGAAAAGGAACGACAACATTTAGTGGAACCGTG
CFSAN049301    1401 TGAAGTAAGCTATACCTTTAGCCAACCTGTCACTATTGGAAAAGGAACGACAACATTTAGTGGAACCGTG

ScottA_ST2     1471 ACGCAGCCACTTAAGGCAATTTTTAATGCTAAGTTTCATGTGGACGGCAAAGAAACAACCAAAGAAGTGG
EGD-e_ST35     1471 ACGCAGCCACTTAAGGCAATTTTTAATGTTAAGTTTCATGTGGACGGCAAAGAAACAACCAAAGAAGTGG
10403S_ST85    1471 ACGCAGCCACTTAAGGCAATTTTTAATGCTAAGTTTCATGTGGACGGCAAAGAAACAACCAAAGAAGTGG
F2365_ST1      1471 ACGCAGCCACTTAAGGCAATTTTTAATGCTAAGTTTCATGTGGACGGCAAAGAAACAACCAAAGAAGTGG
L6-13          1471 ACGCAGCCACTTAAGGCAATTTTTAATGTTAAGTTTCATGTGGACGGCAAAGAAACAACCAAAGAAGTGG
MRL-14-00459   1471 ACGCAGCCACTTAAGGCAATTTTTAATGTTAAGTTTCATGTGGACGGCAAAGAAACAACCAAAGAAGTGG
CFSAN045864    1471 ACGCAGCCACTTAAGGCAATTTTTAATGTTAAGTTTCATGTGGACGGCAAAGAAACAACCAAAGAAGTGG
CFSAN049281    1471 ACGCAGCCACTTAAGGCAATTTTTAATGTTAAGTTTCATGTGGACGGCAAAGAAACAACCAAAGAAGTGG
CFSAN049294    1471 ACGCAGCCACTTAAGGCAATTTTTAATGTTAAGTTTCATGTGGACGGCAAAGAAACAACCAAAGAAGTGG
CFSAN049301    1471 ACGCAGCCACTTAAGGCAATTTTTAATGTTAAGTTTCATGTGGACGGCAAAGAAACAACCAAAGAAGTGG

ScottA_ST2     1541 AAGCTGGGAATTTATTGACTGAACCAGCTAAGCCTGTAAAAGAAGGTTATACATTTGTTGGGTGGTTTGA
EGD-e_ST35     1541 AAGCTGGGAATTTATTGACTGAACCAGCTAAGCCCGTAAAAGAAGGTCACACATTTGTTGGTTGGTTTGA
10403S_ST85    1541 AAGCTGGGAATTTATTGACTGAACCAGCTAAGCCCGTAAAAGAAGGTCACACATTTGTTGGTTGGTTTGA
F2365_ST1      1541 AAGCTGGGAATTTATTGACTGAACCAGCTAAGCCTGTAAAAGAAGGTTATACATTTGTTGGGTGGTTTGA
L6-13          1541 AAGCTGGGAATTTATTGACTGAACCAGCTAAGCCCGTAAAAGAAGGTCACACATTTGTTGGTTGGTTTGA
MRL-14-00459   1541 AAGCTGGGAATTTATTGACTGAACCAGCTAAGCCCGTAAAAGAAGGTCACACATTTGTTGGTTGGTTTGA
CFSAN045864    1541 AAGCTGGGAATTTATTGACTGAACCAGCTAAGCCCGTAAAAGAAGGTCACACATTTGTTGGTTGGTTTGA
CFSAN049281    1541 AAGCTGGGAATTTATTGACTGAACCAGCTAAGCCCGTAAAAGAAGGTCACACATTTGTTGGTTGGTTTGA
CFSAN049294    1541 AAGCTGGGAATTTATTGACTGAACCAGCTAAGCCCGTAAAAGAAGGTCACACATTTGTTGGTTGGTTTGA
CFSAN049301    1541 AAGCTGGGAATTTATTGACTGAACCAGCTAAGCCCGTAAAAGAAGGTCACACATTTGTTGGTTGGTTTGA

ScottA_ST2     1611 TGCCCAAACCGGCGGAACTAAATGGAATTTCAGTACGGATAAAATGCCGACAAATGACATCGATTTATAT
EGD-e_ST35     1611 TGCCCAAACAGGCGGAACTAAATGGAATTTCAGTACGGATAAAATGCCGACAAATGACATCAATTTATAT
10403S_ST85    1611 TGCCCAAACAGGCGGAACTAAATGGAATTTCAGTACGGATAAAATGCCGACAAATGACATCAATTTATAT
F2365_ST1      1611 TGCCCAAACCGGCGGAACTAAATGGAATTTCAGTACGGATAAAATGCCGACAAATGACATCGATTTATAT
L6-13          1611 TGCCCAAACAGGCGGAACTAAATGGGATTTCAGTACGGATAAAATGCCGACAAACGACATCGATTTATAT
MRL-14-00459   1611 TGCCCAAACAGGCGGAACTAAATGGGATTTCAGTACGGATAAAATGCCGACAAACGACATCGATTTATAT
CFSAN045864    1611 TGCCCAAACAGGCGGAACTAAATGGGATTTCAGTACGGATAAAATGCCGACAAACGACATCGATTTATAT
CFSAN049281    1611 TGCCCAAACAGGCGGAACTAAATGGGATTTCAGTACGGATAAAATGCCGACAAACGACATCGATTTATAT
CFSAN049294    1611 TGCCCAAACAGGCGGAACTAAATGGGATTTCAGTACGGATAAAATGCCGACAAACGACATCGATTTATAT
CFSAN049301    1611 TGCCCAAACAGGCGGAACTAAATGGGATTTCAGTACGGATAAAATGCCGACAAACGACATCGATTTATAT

ScottA_ST2     1681 GCGCAATTTAGTATTAACAGCTACACAGCAACGTTTGATAATGACGGTGTAACAACATCTCAAACAGTAG
EGD-e_ST35     1681 GCACAATTTAGTATTAACAGCTACACAGCAACCTTTGATAATGACGGTGTAACAACATCTCAAACAGTAG
10403S_ST85    1681 GCACAATTTAGTATTAACAGCTACACAGCAACCTTTGAGAATGACGGTGTAACAACATCTCAAACAGTAG
F2365_ST1      1681 GCGCAATTTAGTATTAACAGCTACACAGCAACGTTTGATAATGACGGTGTAACAACATCTCAAACAGTAG
L6-13          1681 GCGCAATTTAGTATTAACAGCTACACAGCAACGTTTGATAATGACGGTGTAACAACATCTCAAACAGTAG
MRL-14-00459   1681 GCGCAATTTAGTATTAACAGCTACACAGCAACGTTTGATAATGACGGTGTAACAACATCTCAAACAGTAG
CFSAN045864    1681 GCGCAATTTAGTATTAACAGCTACACAGCAACGTTTGATAATGACGGTGTAACAACATCTCAAACAGTAG
CFSAN049281    1681 GCGCAATTTAGTATTAACAGCTACACAGCAACGTTTGATAATGACGGTGTAACAACATCTCAAACAGTAG
CFSAN049294    1681 GCGCAATTTAGTATTAACAGCTACACAGCAACGTTTGATAATGACGGTGTAACAACATCTCAAACAGTAG
CFSAN049301    1681 GCGCAATTTAGTATTAACAGCTACACAGCAACGTTTGATAATGACGGTGTAACAACATCTCAAACAGTAG

ScottA_ST2     1751 ATTATCAAGGCTTGCTACAAGAACCTACGGCACCAACAAAAGAAGGTTATACTTTCAAAGGCTGGTATGA
EGD-e_ST35     1751 ATTATCAAGGCTTGTTACAAGAACCTACGGCACCAACAAAAGAAGGTTATACTTTTAAAGGCTGGTATGA
10403S_ST85    1751 ATTATCAAGGCTTGTTACAAGAACCTACACCACCAACAAAAGAAGGTTATACTTTCAAAGGCTGGTATGA
F2365_ST1      1751 ATTATCAAGGCTTGCTACAAGAACCTACGGCACCAACAAAAGAAGGTTATACTTTCAAAGGCTGGTATGA
L6-13          1751 ATTATCAAGGCTTGCTACAAGAACCTACGGCACCAACAAAAGAAGGTTATACTTTCAAAGGCTGGTATGA
MRL-14-00459   1751 ATTATCAAGGCTTGCTACAAGAACCTACGGCACCAACAAAAGAAGGTTATACTTTCAAAGGCTGGTATGA
CFSAN045864    1751 ATTATCAAGGCTTGCTACAAGAACCTACGGCACCAACAAAAGAAGG------------------------
CFSAN049281    1751 ATTATCAAGGCTTGCTACAAGAACCTACGGCACCAACAAAAGAAGG------------------------
CFSAN049294    1751 ATTATCAAGGCTTGCTACAAGAACCTACGGCACCAACAAAAGAAGG------------------------
CFSAN049301    1751 ATTATCAAGGCTTGCTACAAGAACCTACGGCACCAACGAAAGCAGG------------------------

ScottA_ST2     1821 CGCAAAAACTGGTGGTGACAAGTGGGATTTTGCAACTAGTAAGATGCCTGCTAAAAACATCACCTTATAT
EGD-e_ST35     1821 CGCAAAAACTGGTGGTGACAAGTGGGATTTCGCAACTAGCAAAATGCCTGCTAAAAACATCACCTTATAT
10403S_ST85    1821 CGCAAAAACTGGTGGTGACAAGTGGGATTTCGCAACTAGCAAAATGCCTGCTAAAAACATCACCTTATAT
F2365_ST1      1821 CGCAAAAACTGGTGGTGACAAGTGGGATTTTGCAACTAGTAAGATGCCTGCTAAAAACATCACCTTATAT
L6-13          1821 CGCAAAAACTGGTGGTGACAAGTGGGATTTCGCAACTAGCAAAATGCCTGCTAAAAACATCACCTTATAT
MRL-14-00459   1821 CGCAAAAACTGGTGGTGACAAGTGGGATTTCGCAACTAGCAAAATGCCTGCTAAAAACATCACCTTATAT
CFSAN045864    1797 ----------------------------------------------------------------------
CFSAN049281    1797 ----------------------------------------------------------------------
CFSAN049294    1797 ----------------------------------------------------------------------
CFSAN049301    1797 ----------------------------------------------------------------------

ScottA_ST2     1891 GCTCAATATAGCGCCAATAGCTATACAGCAACCTTTGATGTTGATGGAAAAACAACGACTCAAGCAGTAG
EGD-e_ST35     1891 GCCCAATATAGCGCCAATAGCTATACAGCAACGTTTGATGTTGATGGAAAATCAACGACTCAAGCAGTAG
```

```
10403S_ST85  1891 GCCCAATATAGCGCCAATAGCTATACAGCAACGTTTGATGTTGATGGAAAATCAACGACTCAAGCAGTAG
F2365_ST1    1891 GCTCAATATAGCGCCAATAGCTATACAGCAACCTTTGATGTTGATGGAAAAACAACGACTCAAGCAGTAG
L6-13        1891 GCCCAATATAGCGCCAATAGCTATACAGCAACGTTTGATATTGATGGAAAAACAACGACTCAAACAGTAG
MRL-14-00459 1891 GCCCAATATAGCGCCAATAGCTATACAGCAACGTTTGATATTGATGGAAAAACAACGACTCAAACAGTAG
CFSAN045864  1797 ----------------------------------------------------------------------
CFSAN049281  1797 ----------------------------------------------------------------------
CFSAN049294  1797 ----------------------------------------------------------------------
CFSAN049301  1797 ----------------------------------------------------------------------

ScottA_ST2   1961 ACTATCAAGGACTTCTAAAAGAACCAAAAACGCCAACAAAAGCCGGATATACTTTCAAAGGTTGGTATGA
EGD-e_ST35   1961 ACTATCAAGGACTTCTAAAAGAACCAAAGGCACCAACGAAAGCCGGATATACTTTCAAAGGCTGGTATGA
10403S_ST85  1961 ACTATCAAGGACTTCTAAAAGAACCAAAGGCACCAACGAAAGCCGGATATACTTTCAAAGGCTGGTATGA
F2365_ST1    1961 ACTATCAAGGACTTCTAAAAGAACCAAAAACGCCAACAAAAGCCGGATATACTTTCAAAGGTTGGTATGA
L6-13        1961 ACTATCAAGGACTTCTAAAAGAACCAAAGGCACCAACGAAAGCCGGATATACTTTCAAAGGCTGGTATGA
MRL-14-00459 1961 ACTATCAAGGACTTCTAAAAGAACCAAAGGCACCAACGAAAGCCGGATATACTTTCAAAGGCTGGTATGA
CFSAN045864  1797 ----------------------------------------------TTATACTTTCAAAGGCTGGTATGA
CFSAN049281  1797 ----------------------------------------------TTATACTTTCAAAGGCTGGTATGA
CFSAN049294  1797 ----------------------------------------------TTATACTTTCAAAGGCTGGTATGA
CFSAN049301  1797 ----------------------------------------------ATATACTTTCAAAGGCTGGTATGA

ScottA_ST2   2031 CGAAAAAACAGATGGTAAAAAATGGGATTTTGCGACAGATAAAATGCCAGCAAATGATATTACGCTGTAC
EGD-e_ST35   2031 CGAAAAAACAGATGGGAAAAAATGGGATTTTGCGACGGATAAAATGCCAGCAAATGACATTACGCTGTAC
10403S_ST85  2031 CGAAAAAACAGATGGGAAAAAATGGGATTTTGCGACGGATAAAATGCCAGCAAATGACATTACGCTGTAC
F2365_ST1    2031 CGAAAAAACAGATGGTAAAAAATGGGATTTTGCGACAGATAAAATGCCAGCAAATGATATTACGCTGTAC
L6-13        2031 CGAAAAAACAGATGGGAAAAAATGGGATTTTGCGACGGATAAAATGCCAGCAAATGACATTACGCTGTAC
MRL-14-00459 2031 CGAAAAAACAGATGGGAAAAAATGGGATTTTGCGACGGATAAAATGCCAGCAAATGACATTACGCTGTAC
CFSAN045864  1821 CGAAAAAACAGATGGGAAAAAATGGGATTTTGCGACGGATAAAATGCCAGCAAATGACATTACGCTGTAC
CFSAN049281  1821 CGAAAAAACAGATGGGAAAAAATGGGATTTTGCGACGGATAAAATGCCAGCAAATGACATTACGCTGTAC
CFSAN049294  1821 CGAAAAAACAGATGGGAAAAAATGGGATTTTGCGACGGATAAAATGCCAGCAAATGACATTACGCTGTAC
CFSAN049301  1821 CGAAAAAACAGATGGGAAAAAATGGGATTTTGCGACGGATAAAATGCCAGCAAATGACATTACGCTGTAC

ScottA_ST2   2101 GCTCAATTCACGAAAAATCCTGTGGCACCACCAACAACTGGAGGGAACACTCCGCCGACTACAAATAACG
EGD-e_ST35   2101 GCTCAATTTACGAAAAATCCTGTGGCACCACCAACAACTGGAGGGAACACACCGCCTACAACAAATAACG
10403S_ST85  2101 GCTCAATTTACGAAAAATCCTGTGGCACCACCAACAACTGGAGGGAACACACCGCCTACAACAAATAACG
F2365_ST1    2101 GCTCAATTCACGAAAAATCCTGTGGCACCACCAACAACTGGAGGGAACACTCCGCCGACTACAAATAACG
L6-13        2101 GCTCAATTTACGAAAAATCCTGTGGCACCACCAACAACTGGAGGGAACACACCGCCTACAACAAATAACG
MRL-14-00459 2101 GCTCAATTTACGAAAAATCCTGTGGCACCACCAACAACTGGAGGGAACACACCGCCTACAACAAATAACG
CFSAN045864  1891 GCTCAATTTACGAAAAATCCTGTGGCACCACCAACAACTGGAGGGAACACACCGCCTACAACAAATAACG
CFSAN049281  1891 GCTCAATTTACGAAAAATCCTGTGGCACCACCAACAACTGGAGGGAACACACCGCCTACAACAAATAACG
CFSAN049294  1891 GCTCAATTTACGAAAAATCCTGTGGCACCACCAACAACTGGAGGGAACACACCGCCTACAACAAATAACG
CFSAN049301  1891 GCTCAATTTACGAAAAATCCTGTGGCACCACCAACAACTGGAGGGAACACACCGCCTACAACAAATAACG

ScottA_ST2   2171 GAGGGAACACTACACCACCTTCCGCAAATATACCTGGAAGCAACACATCTAACACATCAACTGGGAATTC
EGD-e_ST35   2171 GCGGGAATACTACACCACCTTCCGCAAATATACCTGGAAGCGACACATCTAACACATCAACTGGGAATTC
10403S_ST85  2171 GCGGGAATACTACACCACCTTCCGCAAATATACCTGGAAGCGACACATCTAACACATCAACTGGGAATTC
F2365_ST1    2171 GAGGGAACACTACACCACCTTCCGCAAATATACCTGGAAGCAACACATCTAACACATCAACTGGGAATTC
L6-13        2171 GCGGGAATACTACACCACCTTCCGCAAATATACCTGGAAGCGACACATCTAACACATCAACTGGGAATTC
MRL-14-00459 2171 GCGGGAATACTACACCACCTTCCGCAAATATACCTGGAAGCGACACATCTAACACATCAACTGGGAATTC
CFSAN045864  1961 GCGGGAATACTACACCACCTTCCGCAAATATACCTGGAAGCGACACATCTAACACATCAACTGGGAATTC
CFSAN049281  1961 GCGGGAATACTACACCACCTTCCGCAAATATACCTGGAAGCGACACATCTAACACATCAACTGGGAATTC
CFSAN049294  1961 GCGGGAATACTACACCACCTTCCGCAAATATACCTGGAAGCGACACATCTAACACATCAACTGGGAATTC
CFSAN049301  1961 GCGGGAATACTACACCACCTTCCGCAAATATACCTGGAAGCGACACATCTAACACATCAACTGGGAATTC

ScottA_ST2   2241 AGCTAGCACAACAAGTACAATGAACGCTTATGACCCTTATAATTCAAAAGAAGCTTCACTCCCTACAACT
EGD-e_ST35   2241 AGCCAGCACAACAAGTACAATGAACGCTTATGACCCTTATAATTCAAAAGAAGCTTCACTCCCTACAACT
10403S_ST85  2241 AGCCAGCACAACAAGTACAATGAACGCTTATGACCCTTATAATTCAAAAGAAGCTTCACTCCCTACAACT
F2365_ST1    2241 AGCTAGCACAACAAGTACAATGAACGCTTATGACCCTTATAATTCAAAAGAAGCTTCACTCCCTACAACT
L6-13        2241 AGCCAGCACAACAAGTACAATGAACGCTTATGACCCTTATAATTCAAAAGAAGCTTCACTCCCTACAACT
MRL-14-00459 2241 AGCCAGCACAACAAGTACAATGAACGCTTATGACCCTTATAATTCAAAAGAAGCTTCACTCCCTACAACT
CFSAN045864  2031 AGCCAGCACAACAAGTACAATGAACGCTTATGACCCTTATAATTCAAAAGAAGCTTCACTCCCTACAACT
CFSAN049281  2031 AGCCAGCACAACAAGTACAATGAACGCTTATGACCCTTATAATTCAAAAGAAGCTTCACTCCCTACAACT
CFSAN049294  2031 AGCCAGCACAACAAGTACAATGAACGCTTATGACCCTTATAATTCAAAAGAAGCTTCACTCCCTACAACT
CFSAN049301  2031 AGCCAGCACAACAAGTACAATGAACGCTTATGACCCTTATAATTCAAAAGAAGCTTCACTCCCTACAACT

ScottA_ST2   2311 GGTGATAGCGATAATGCGCTCTACCTGTTGATAGGATTGTTAGCAGTAGGAACTGCAGTGGCTCTTACTA
EGD-e_ST35   2311 GGCGATAGCGATAATGCGCTCTACCTTTTGTTAGGGTTATTAGCAGTAGGAACTGCAATGGCTCTTACTA
10403S_ST85  2311 GGCGATAGCGATAATGCGCTCTACCTTTTGTTAGGGTTATTAGCAGTAGGAACTGCAATGGCTCTTACTA
F2365_ST1    2311 GGTGATAGCGATAATGCGCTCTACCTGTTGATAGGATTGTTAGCAGTAGGAACTGCAGTGGCTCTTACTA
L6-13        2311 GGCGATAGCGATAATGCGCTCTACCTTTTGTTAGGGTTATTAGCAGTAGGAACTGCAATGGCTCTTACTA
MRL-14-00459 2311 GGCGATAGCGATAATGCGCTCTACCTTTTGTTAGGGTTATTAGCAGTAGGAACTGCAATGGCTCTTACTA
CFSAN045864  2101 GGCGATAGCGATAATGCGCTCTACCTTTTGTTAGGGTTATTAGCAGTAGGAACTGCAATGGCTCTTACTA
CFSAN049281  2101 GGCGATAGCGATAATGCGCTCTACCTTTTGTTAGGGTTATTAGCAGTAGGAACTGCAATGGCTCTTACTA
CFSAN049294  2101 GGCGATAGCGATAATGCGCTCTACCTTTTGTTAGGGTTATTAGCAGTAGGAACTGCAATGGCTCTTACTA
CFSAN049301  2101 GGCGATAGCGATAATGCGCTCTACCTTTTGTTAGGGTTATTAGCAGTAGGAACTGCAATGGCTCTTACTA
```

ScottA_ST2	2381	AAAAAGCACGTGCTAGTAAATAG
EGD-e_ST35	2381	AAAAAGCACGTGCTAGTAAATAG
10403S_ST85	2381	AAAAAGCACGTGCTAGTAAATAG
F2365_ST1	2381	AAAAAGCACGTGCTAGTAAATAG
L6-13	2381	AAAAAGCACGTGCTAGTAAATAG
MRL-14-00459	2381	AAAAAGCACGTGCTAGTAAATAG
CFSAN045864	2171	AAAAAGCACGTGCTAGTAAATAG
CFSAN049281	2171	AAAAAGCACGTGCTAGTAAATAG
CFSAN049294	2171	AAAAAGCACGTGCTAGTAAATAG
CFSAN049301	2171	AAAAAGCACGTGCTAGTAAATAG

Figure S2B. Alignment of InlA protein sequences

```
ScottA_ST2      1 VRRKRYVWLKSILVAILVFGSGVWINTSNGTNAQAATITQDTPINQIFTDTALAEKMKTVLGKTNVTDTV
EGD-e_ST35      1 VRKKRYVWLKSILVAILVFGSGVWINTSNGTNAQAATITQDTPINQIFTDTALAEKMKTVLGKTNVTDTV
10403S_ST85     1 VRKKRYVWLKSILVAILVFGSGVWINTSNGTNAQAATITQDTPINQIFTDAALAEKMKTVLGKTNVTDTV
F2365_ST1       1 VRRKRYVWLKSILVAILVFGSGVWINTSNGTNAQAATITQDTPINQIFTDTALAEKMKTVLGKTNVTDTV
L6-13           1 VRKKRYVWLKSILVAILVFGSGVWINTSNGTNAQAATITQDTPINQIFTDAALAEKMKTVLGKTNVTDTV
MRL-14-00459    1 VRKKRYVWLKSILVAILVFGSGVWINTSNGTNAQAATITQDTPINQIFTDAALAEKMKTVLGKTNVTDTV
CFSAN045864     1 VRKKRYVWLKSILVAILVFGSGVWINTSNGTNAQAATITQDTPINQIFTDAALAEKMKTVLGKTNVTDTV
CFSAN049281     1 VRKKRYVWLKSILVAILVFGSGVWINTSNGTNAQAATITQDTPINQIFTDAALAEKMKTVLGKTNVTDTV
CFSAN049294     1 VRKKRYVWLKSILVAILVFGSGVWINTSNGTNAQAATITQDTPINQIFTDAALAEKMKTVLGKTNVTDTV
CFSAN049301     1 VRKKRYVWLKSILVAILVFGSGVWINTSNGTNAQAATITQDTPINQIFTDAALAEKMKTVLGKTNVTDTV

ScottA_ST2     71 SQTDLDQVTTLQADRLGIKSIDGLEYLNNLTQINFSNNQLTDITPLKDLTKLVDILMNNNQIADITPLAN
EGD-e_ST35     71 SQTDLDQVTTLQADRLGIKSIDGVEYLNNLTQINFSNNQLTDITPLKNLTKLVDILMNNNQIADITPLAN
10403S_ST85    71 SQTDLDQVTTLQADRLGIKSIDGLEYLNNLTQINFSNNQLTDITPLKDLTKLVDILMNNNQIADITPLAN
F2365_ST1      71 SQTDLDQVTTLQADRLGIKSIDGLEYLNNLTQINFSNNQLTDITPLKDLTKLVDILMNNNQIADITPLAN
L6-13          71 SQTDLDQVTTLQADRLGIKSIDGVEYLNNLTQINFSNNQLTDITPLKNLTKLVDILMNNNQIADITPLAN
MRL-14-00459   71 SQTDLDQVTTLQADRLGIKSIDGVEYLNNLTQINFSNNQLTDITPLKNLTKLVDILMNNNQIADITPLAN
CFSAN045864    71 SQTDLDQVTTLQADRLGIKSIDGVEYLNNLTQINFSNNQLTDITPLKNLTKLVDILMNNNQIADITPLAN
CFSAN049281    71 SQTDLDQVTTLQADRLGIKSIDGVEYLNNLTQINFSNNQLTDITPLKNLTKLVDILMNNNQIADITPLAN
CFSAN049294    71 SQTDLDQVTTLQADRLGIKSIDGVEYLNNLTQINFSNNQLTDITPLKNLTKLVDILMNNNQIADITPLAN
CFSAN049301    71 SQTDLDQVTTLQADRLGIKSIDGVEYLNNLTQINFSNNQLTDITPLKNLTKLVDILMNNNQIADITPLAN

ScottA_ST2    141 LSNLTGLTLFNNQITDIDPLKNLTNLNRLELSSNTISDISALSGLTSLQQLSFGNQVTDLKPLANLTTLE
EGD-e_ST35    141 LTNLTGLTLFNNQITDIDPLKNLTNLNRLELSSNTISDISALSGLTSLQQLSFGNQVTDLKPLANLTTLE
10403S_ST85   141 LTNLTGLTLFNNQITDIDPLKNLTNLNRLELSSNTISDISALSGLTNLQQLSFGNQVTDLKPLANLTTLE
F2365_ST1     141 LSNLTGLTLFNNQITDIDPLKNLTNLNRLELSSNTISDISALSGLTSLQQLSFGNQVTDLKPLANLTTLE
L6-13         141 LTNLTGLTLFNNQITDLDPLKNLTNLNRLELSSNTISDISALSGLTSLQQLSFGNQVTDLKPLANLTTLE
MRL-14-00459  141 LTNLTGLTLFNNQITDLDPLKNLTNLNRLELSSNTISDISALSGLTSLQQLSFGNQVTDLKPLANLTTLE
CFSAN045864   141 LTNLTGLTLFNNQITDLDPLKNLTNLNRLELSSNTISDISALSGLTSLQQLSFGNQVTDLKPLANLTTLE
CFSAN049281   141 LTNLTGLTLFNNQITDLDPLKNLTNLNRLELSSNTISDISALSGLTSLQQLSFGNQVTDLKPLANLTTLE
CFSAN049294   141 LTNLTGLTLFNNQITDLDPLKNLTNLNRLELSSNTISDISALSGLTSLQQLSFGNQVTDLKPLANLTTLE
CFSAN049301   141 LTNLTGLTLFNNQITDLDPLKNLTNLNRLELSSNTISDISALSGLTSLQQLSFGNQVTDLKPLANLTTLE

ScottA_ST2    211 RLDISSNKVSDISVLAKLTNLESLIATNNQISDITPLGILTNLDELSLNGNQLKDIGTLASLTNLTDLDL
EGD-e_ST35    211 RLDISSNKVSDISVLAKLTNLESLIATNNQISDITPLGILTNLDELSLNGNQLKDIGTLASLTNLTDLDL
10403S_ST85   211 RLDISSNKVSDISVLAKLTNLESLIATNNQISDITPLGILTNLDELSLNGNQLKDIGTLASLTNLTDLDL
F2365_ST1     211 RLDISSNKVSDISVLAKLTNLESLIATNNQISDITPLGILTNLDELSLNGNQLKDIGTLASLTNLTDLDL
L6-13         211 RLDISSNKVSDISVLAKLTNLESLIATNNQISDITPLGILTNLDELSLNGNQLKDIGTLASLTNLTDLDL
MRL-14-00459  211 RLDISSNKVSDISVLAKLTNLESLIATNNQISDITPLGILTNLDELSLNGNQLKDIGTLASLTNLTDLDL
CFSAN045864   211 RLDISSNKVSDISVLAKLTNLESLIATNNQISDITPLGILTNLDELSLNGNQLKDIGTLASLTNLTDLDL
CFSAN049281   211 RLDISSNKVSDISVLAKLTNLESLIATNNQISDITPLGILTNLDELSLNGNQLKDIGTLASLTNLTDLDL
CFSAN049294   211 RLDISSNKVSDISVLAKLTNLESLIATNNQISDITPLGILTNLDELSLNGNQLKDIGTLASLTNLTDLDL
CFSAN049301   211 RLDISSNKVSDISVLAKLTNLESLIATNNQISDITPLGILTNLDELSLNGNQLKDIGTLASLTNLTDLDL

ScottA_ST2    281 ANNQISNLAPLSGLTKLTELKLGANQISNISPLAGLTALTNLELNENQLEDISPISNLKNLTYLTLYFNN
EGD-e_ST35    281 ANNQISNLAPLSGLTKLTELKLGANQISNISPLAGLTALTNLELNENQLEDISPISNLKNLTYLTLYFNN
10403S_ST85   281 ANNQISNLAPLSGLTKLTELKLGANQISNISPLAGLTALTNLELNENQLEDISPISNLKNLTYLTLYFNN
F2365_ST1     281 ANNQISNLAPLSGLTKLTELKLGANQISNISPLAGLTALTNLELNENQLEDISPISNLKNLTYLTLYFNN
L6-13         281 ANNQISNLAPLSGLTKLTELKLGANQISNISPLAGLTALTNLELNENQLEDISPISNLKNLTYLTLYFNN
MRL-14-00459  281 ANNQISNLAPLSGLTKLTELKLGANQISNISPLAGLTALTNLELNENQLEDISPISNLKNLTYLTLYFNN
CFSAN045864   281 ANNQISNLAPLSGLTKLTELKLGANQISNISPLAGLTALTNLELNENQLEDISPISNLKNLTYLTLYFNN
CFSAN049281   281 ANNQISNLAPLSGLTKLTELKLGANQISNISPLAGLTALTNLELNENQLEDISPISNLKNLTYLTLYFNN
CFSAN049294   281 ANNQISNLAPLSGLTKLTELKLGANQISNISPLAGLTALTNLELNENQLEDISPISNLKNLTYLTLYFNN
CFSAN049301   281 ANNQISNLAPLSGLTKLTELKLGANQISNISPLAGLTALTNLELNENQLEDISPISNLKNLTYLTLYFNN

ScottA_ST2    351 ISDISPVSSLTKLQRLFFYNNKVSDVSSLANLTNINWLSAGHNQISDLTPLANLTRITQLGLNDQEWTNP
EGD-e_ST35    351 ISDISPVSSLTKLQRLFFYNNKVSDVSSLANLTNINWLSAGHNQISDLTPLANLTRITQLGLNDQAWTNA
10403S_ST85   351 ISDISPVSSLTKLQRLFFYNNKVSDVSSLANLTNINWLSAGHNQISDLTPLANLTRITQLGLNDQAWTNA
F2365_ST1     351 ISDISPVSSLTKLQRLFFYNNKVSDVSSLANLTNINWLSAGHNQISDLTPLANLTRITQLGLNDQEWTNP
L6-13         351 ISDISPVSSLTKLQRLFFYNNKVSDVSSLANLTNINWLSAGHNQISDLTPLANLTRITQLGLNDQAWTNA
MRL-14-00459  351 ISDISPVSSLTKLQRLFFYNNKVSDVSSLANLTNINWLSAGHNQISDLTPLANLTRITQLGLNDQAWTNA
CFSAN045864   351 ISDISPVSSLTKLQRLFFYNNKVSDVSSLANLTNINWLSAGHNQISDLTPLANLTRITQLGLNDQAWTNA
CFSAN049281   351 ISDISPVSSLTKLQRLFFYNNKVSDVSSLANLTNINWLSAGHNQISDLTPLANLTRITQLGLNDQAWTNA
CFSAN049294   351 ISDISPVSSLTKLQRLFFYNNKVSDVSSLANLTNINWLSAGHNQISDLTPLANLTRITQLGLNDQAWTNA
CFSAN049301   351 ISDISPVSSLTKLQRLFFYNNKVSDVSSLANLTNINWLSAGHNQISDLTPLANLTRITQLGLNDQAWTNA

ScottA_ST2    421 PVNYKVNVSIPNTVKNVTGALIAPATISDGGSYAEPDITWNLPSYTNEVSYTFNQSVTIGKGTTTFSGTV
EGD-e_ST35    421 PVNYKANVSIPNTVKNVTGALIAPATISDGGSYTEPDITWNLPSYTNEVSYTFSQPVTIGKGTTTFSGTV
10403S_ST85   421 PVNYKANVSIPNTVKNVTGALIAPATISDGGSYAEPDITWNLPSYTNEVSYTFSQPVTIGKGTTTFSGTV
F2365_ST1     421 PVNYKVNVSIPNTVKNVTGALIAPATISDGGSYAEPDITWNLPSYTNEVSYTFNQSVTIGKGTTTFSGTV
L6-13         421 PVNYKANVSIPNTVKNVTGALIAPATISDGGSYTEPDITWNLPSYTNEVSYTFSQPVTIGKGTTTFSGTV
MRL-14-00459  421 PVNYKANVSIPNTVKNVTGALIAPATISDGGSYTEPDITWNLPSYTNEVSYTFSQPVTIGKGTTTFSGTV
CFSAN045864   421 PVNYKANVSIPNTVKNVTGALIAPATISDGGSYTEPDITWNLPSYTNEVSYTFSQPVTIGKGTTTFSGTV
```

```
CFSAN049281   421 PVNYKANVSIPNTVKNVTGALIAPATISDGGSYTEPDITWNLPSYTNEVSYTFSQPVTIGKGTTTFSGTV
CFSAN049294   421 PVNYKANVSIPNTVKNVTGALIAPATISDGGSYTEPDITWNLPSYTNEVSYTFSQPVTIGKGTTTFSGTV
CFSAN049301   421 PVNYKANVSIPNTVKNVTGALIAPATISDGGSYTEPDITWNLPSYTNEVSYTFSQPVTIGKGTTTFSGTV

ScottA_ST2    491 TQPLKAIFNAKFHVDGKETTKEVEAGNLLTEPAKPVKEGYTFVGWFDAQTGGTKWNFSTDKMPTNDIDLY
EGD-e_ST35    491 TQPLKAIFNVKFHVDGKETTKEVEAGNLLTEPAKPVKEGHTFVGWFDAQTGGTKWNFSTDKMPTNDINLY
10403S_ST85   491 TQPLKAIFNAKFHVDGKETTKEVEAGNLLTEPAKPVKEGHTFVGWFDAQTGGTKWNFSTDKMPTNDINLY
F2365_ST1     491 TQPLKAIFNAKFHVDGKETTKEVEAGNLLTEPAKPVKEGYTFVGWFDAQTGGTKWNFSTDKMPTNDIDLY
L6-13         491 TQPLKAIFNVKFHVDGKETTKEVEAGNLLTEPAKPVKEGHTFVGWFDAQTGGTKWDFSTDKMPTNDIDLY
MRL-14-00459  491 TQPLKAIFNVKFHVDGKETTKEVEAGNLLTEPAKPVKEGHTFVGWFDAQTGGTKWDFSTDKMPTNDIDLY
CFSAN045864   491 TQPLKAIFNVKFHVDGKETTKEVEAGNLLTEPAKPVKEGHTFVGWFDAQTGGTKWDFSTDKMPTNDIDLY
CFSAN049281   491 TQPLKAIFNVKFHVDGKETTKEVEAGNLLTEPAKPVKEGHTFVGWFDAQTGGTKWDFSTDKMPTNDIDLY
CFSAN049294   491 TQPLKAIFNVKFHVDGKETTKEVEAGNLLTEPAKPVKEGHTFVGWFDAQTGGTKWDFSTDKMPTNDIDLY
CFSAN049301   491 TQPLKAIFNVKFHVDGKETTKEVEAGNLLTEPAKPVKEGHTFVGWFDAQTGGTKWDFSTDKMPTNDIDLY

ScottA_ST2    561 AQFSINSYTATFDNDGVTTSQTVDYQGLLQEPTAPTKEGYTFKGWYDAKTGGDKWDFATSKMPAKNITLY
EGD-e_ST35    561 AQFSINSYTATFDNDGVTTSQTVDYQGLLQEPTAPTKEGYTFKGWYDAKTGGDKWDFATSKMPAKNITLY
10403S_ST85   561 AQFSINSYTATFENDGVTTSQTVDYQGLLQEPTPPTKEGYTFKGWYDAKTGGDKWDFATSKMPAKNITLY
F2365_ST1     561 AQFSINSYTATFDNDGVTTSQTVDYQGLLQEPTAPTKEGYTFKGWYDAKTGGDKWDFATSKMPAKNITLY
L6-13         561 AQFSINSYTATFDNDGVTTSQTVDYQGLLQEPTAPTKEGYTFKGWYDAKTGGDKWDFATSKMPAKNITLY
MRL-14-00459  561 AQFSINSYTATFDNDGVTTSQTVDYQGLLQEPTAPTKEGYTFKGWYDAKTGGDKWDFATSKMPAKNITLY
CFSAN045864   561 AQFSINSYTATFDNDGVTTSQTVDYQGLLQEPTA------------------------------------
CFSAN049281   561 AQFSINSYTATFDNDGVTTSQTVDYQGLLQEPTA------------------------------------
CFSAN049294   561 AQFSINSYTATFDNDGVTTSQTVDYQGLLQEPTA------------------------------------
CFSAN049301   561 AQFSINSYTATFDNDGVTTSQTVDYQGLLQEPTA------------------------------------

ScottA_ST2    631 AQYSANSYTATFDVDGKTTTQAVDYQGLLKEPKTPTKAGYTFKGWYDEKTDGKKWDFATDKMPANDITLY
EGD-e_ST35    631 AQYSANSYTATFDVDGKSTTQAVDYQGLLKEPKAPTKAGYTFKGWYDEKTDGKKWDFATDKMPANDITLY
10403S_ST85   631 AQYSANSYTATFDVDGKSTTQAVDYQGLLKEPKAPTKAGYTFKGWYDEKTDGKKWDFATDKMPANDITLY
F2365_ST1     631 AQYSANSYTATFDVDGKTTTQAVDYQGLLKEPKTPTKAGYTFKGWYDEKTDGKKWDFATDKMPANDITLY
L6-13         631 AQYSANSYTATFDIDGKTTTQTVDYQGLLKEPKAPTKAGYTFKGWYDEKTDGKKWDFATDKMPANDITLY
MRL-14-00459  631 AQYSANSYTATFDIDGKTTTQTVDYQGLLKEPKAPTKAGYTFKGWYDEKTDGKKWDFATDKMPANDITLY
CFSAN045864   595 ------------------------------------PTKEGYTFKGWYDEKTDGKKWDFATDKMPANDITLY
CFSAN049281   595 ------------------------------------PTKEGYTFKGWYDEKTDGKKWDFATDKMPANDITLY
CFSAN049294   595 ------------------------------------PTKEGYTFKGWYDEKTDGKKWDFATDKMPANDITLY
CFSAN049301   595 ------------------------------------PTKAGYTFKGWYDEKTDGKKWDFATDKMPANDITLY

ScottA_ST2    701 AQFTKNPVAPPTTGGNTPPTTNNGGNTTPPSANIPGSNTSNTSTGNSASTTSTMNAYDPYNSKEASLPTT
EGD-e_ST35    701 AQFTKNPVAPPTTGGNTPPTTNNGGNTTPPSANIPGSDTSNTSTGNSASTTSTMNAYDPYNSKEASLPTT
10403S_ST85   701 AQFTKNPVAPPTTGGNTPPTTNNGGNTTPPSANIPGSDTSNTSTGNSASTTSTMNAYDPYNSKEASLPTT
F2365_ST1     701 AQFTKNPVAPPTTGGNTPPTTNNGGNTTPPSANIPGSNTSNTSTGNSASTTSTMNAYDPYNSKEASLPTT
L6-13         701 AQFTKNPVAPPTTGGNTPPTTNNGGNTTPPSANIPGSDTSNTSTGNSASTTSTMNAYDPYNSKEASLPTT
MRL-14-00459  701 AQFTKNPVAPPTTGGNTPPTTNNGGNTTPPSANIPGSDTSNTSTGNSASTTSTMNAYDPYNSKEASLPTT
CFSAN045864   631 AQFTKNPVAPPTTGGNTPPTTNNGGNTTPPSANIPGSDTSNTSTGNSASTTSTMNAYDPYNSKEASLPTT
CFSAN049281   631 AQFTKNPVAPPTTGGNTPPTTNNGGNTTPPSANIPGSDTSNTSTGNSASTTSTMNAYDPYNSKEASLPTT
CFSAN049294   631 AQFTKNPVAPPTTGGNTPPTTNNGGNTTPPSANIPGSDTSNTSTGNSASTTSTMNAYDPYNSKEASLPTT
CFSAN049301   631 AQFTKNPVAPPTTGGNTPPTTNNGGNTTPPSANIPGSDTSNTSTGNSASTTSTMNAYDPYNSKEASLPTT

ScottA_ST2    771 GDSDNALYLLIGLLAVGTAVALTKKARASK
EGD-e_ST35    771 GDSDNALYLLLGLLAVGTAMALTKKARASK
10403S_ST85   771 GDSDNALYLLLGLLAVGTAMALTKKARASK
F2365_ST1     771 GDSDNALYLLIGLLAVGTAVALTKKARASK
L6-13         771 GDSDNALYLLLGLLAVGTAMALTKKARASK
MRL-14-00459  771 GDSDNALYLLLGLLAVGTAMALTKKARASK
CFSAN045864   701 GDSDNALYLLLGLLAVGTAMALTKKARASK
CFSAN049281   701 GDSDNALYLLLGLLAVGTAMALTKKARASK
CFSAN049294   701 GDSDNALYLLLGLLAVGTAMALTKKARASK
CFSAN049301   701 GDSDNALYLLLGLLAVGTAMALTKKARASK
```

Figure S3A. Alignment of *htrA* nucleotide sequences

```
ScottA_ST2      1 ATGGACGAGAAAGAAAAGAATTTAAATGAAAACAGCGAGAATGAAAGCACGCCAAAAAGAGAGGTCGAGG
EGD-e_ST35      1 ATGGACGAGAAAGAAAAGAATTTAAATGAAAACAGCGAGAATGAAAGCACGCCGAAAAGAGAGGTCGAGG
10403S_ST85     1 ATGGACGAGAAAGAAAAGAATTTAAATGAAAACAGCGAGAATGAAAGCACGCCGAAAAGAGAGGTCGAGG
F2365_ST1       1 ATGGACGAGAAAGAAAAGAATTTAAATGAAAACAGCGAGAATGAAAGCACGCCAAAAAGAGAGGTCGAGG
P12_10_ST155    1 ATGGACGAGAAAGAAAAGAATTTAAATGAAAACAGCGAGAATGAAAGCACGCCGAAAAGAGAGGTCGAGG

ScottA_ST2     71 ATACTTTACATACACCGGAGAGCGCGCAACCAGTCCAAGAAACTCCTATTGTAGAAGGCGTGACCCCAGA
EGD-e_ST35     71 ATACTTTACATACACCGGAGAGCGCGCAACCAGTCCAAGAAACTCCTATTGTAGAAGGCGTGACCCCAGA
10403S_ST85    71 ATACTTTACATACACCGGAGAGCGCGCAACCAGTCCAAGAAACTCCTATTGTAGAAGGCGTGACCCCAGA
F2365_ST1      71 ATACTTTACATACACCGGAGAGCGCGCAACCAGTCCAAGAAACTCCTATTGTAGAAGGCGTGACCCCAGA
P12_10_ST155   71 ATACTTTACATACACCGGAGAGCGCGCAACCAGTCCAAGAAACTCCTATTGTAGAAGGCGTGACCCCAGA

ScottA_ST2    141 AGGGGAGAAATTTGCCGGAGCGACAGAAGATGCAGCCGAGGCGAGTTCTACAAATGCATTTTTTGAAGAA
EGD-e_ST35    141 AGGGGAGAAATTTGCCGGAGCGACAGAAGATGCAGCCGAGGCGAGTTCTACAAATGCATTTTTTGAAGAA
10403S_ST85   141 AGGGGAGAAATTTGCCGGAGCGACAGAAGATGCAGCCGAGGCGAGTTCTACAAATGCATTTTTTGAAGAA
F2365_ST1     141 AGGGGAGAAATTTGCCGGAGCGACAGAAGATGCAGCCGAGGCGAGTTCTACAAATGCATTTTTTGAAGAA
P12_10_ST155  141 AGGGGAGAAATTTGCCGGAGCGACAGAAGATGCAGCCGAGGCGAGTTCTACAAATGCATTTTTTGAAGAA

ScottA_ST2    211 GCAAGTAATAAAGAACCAGAACCTGCTAGACCGGCTCCAGGGCCAAGACGCGCTGGTACAACTGGTGGCG
EGD-e_ST35    211 GCAAGTAATAAAGAATCAGAACCTGCTAGACCGGCTCCAGGGCCAAGACGCGCTGGTACAACTGGTGGCG
10403S_ST85   211 GCAAGTAATAAAGAATCAGAACCTGCTAGACCGGCTCCAGGGCCAAGACGCGCTGGTACAACTGGTGGCG
F2365_ST1     211 GCAAGTAATAAAGAACCAGAACCTGCTAGACCGGCTCCAGGGCCAAGACGCGCTGGTACAACTGGTGGCG
P12_10_ST155  211 GCAAGTAATAAAGAATCAGAACCTGCTAGACCGGCTCCAGGGCCAAGACGCGCTGGTACAACTGGTGGCG

ScottA_ST2    281 GGGCAGTTCCAC---CTAATAGAGTGAATAATGGCGGAAGTGGTAACGGGAATGGTGAACCACCGAAACG
EGD-e_ST35    281 GGGCAGTTCCAC---CTAATAGAGTGAATAATGGCGGAAGTGGTAACGGGAATGGTGAACCACCGAAACG
10403S_ST85   281 GGGCAGTCCCACCTACTAATAGAGTGAATAATGGCGGAAGTGGTAACGGGAATGGTGAACCACCGAAACG
F2365_ST1     281 GGGCAGTTCCAC---CTAATAGAGTGAATAATGGCGGAAGTGGTAACGGGAATGGTGAACCACCGAAACG
P12_10_ST155  281 GGGCAGTCCCACCTACTAATAGAGTAAATAATGGCGGAAGTGGTAACGGGAACGGTGAGCCACCGAAACG

ScottA_ST2    348 CGGCAAACACTTTATCGGTTACTTTTTAACAGCACTTATTGGTGTTATTATCGGAGGACTTATTATTTTC
EGD-e_ST35    348 CGGCAAACACTTTATTGGTTACTTTTTAACAGCACTTATTGGTGTTATTATCGGAGGACTTATTATTTTC
10403S_ST85   351 CGGCAAACACTTTATTGGTTACTTTTTAACAGCACTTATTGGTGTTATTATCGGAGGACTTATTATTTTC
F2365_ST1     348 CGGCAAACACTTTATCGGTTACTTTTTAACAGCACTTATTGGTGTTATTATCGGAGGACTTATTATTTTC
P12_10_ST155  351 CGGCAAACACTTTATTGGTTACTTTTTAACAGCACTTATTGGTGTTATTATCGGAGGACTTATTATTTTC

ScottA_ST2    418 TTTGTCGCTTGGGATAATGGTGACAATGCAGATACAACTTCAAACTCAAATAATAAAGCTACCAAAGTAG
EGD-e_ST35    418 TTTGTCGCTTGGGATAATGGCGATAACGCAGATACAACTTCAAACTCAAATAATAAAGCTACCAAAGTAG
10403S_ST85   421 TTTGTCGCTTGGGATAATGGCGATAACGCAGATACAACTTCAAACTCAAATAATAAAGCTACCAAAGTAG
F2365_ST1     418 TTTGTCGCTTGGGATAATGGTGACAATGCAGATACAACTTCAAACTCAAATAATAAAGCTACCAAAGTAG
P12_10_ST155  421 TTTGTCGCTTGGGATAATGGCGATAACGCAGATACAACTTCAAACTCAAATAATAAAGCTACCAAAGTAG

ScottA_ST2    488 AAAAAGTTTCAGTAGATACAACATCAGATGTAACAAAAGCAGTAGACAAAGTACAAGATGCGGTAGTGAG
EGD-e_ST35    488 AAAAAGTTTCGGTAGATACAACATCAGATGTAACAAAAGCAGTAGACAAAGTGCAAGATGCGGTAGTCAG
10403S_ST85   491 AAAAAGTTTCGGTAGATACAACATCAGATGTAACAAAAGCAGTAGACAAAGTGCAAGATGCGGTGGTCAG
F2365_ST1     488 AAAAAGTTTCAGTAGATACAACATCAGATGTAACAAAAGCAGTAGACAAAGTACAAGATGCGGTAGTGAG
P12_10_ST155  491 AAAAAGTTTCGGTAGATACAACATCAGATGTAACAAAAGCAGTAGACAAAGTGCAAGATGCGGTGGTCAG

ScottA_ST2    558 TGTTCTAAATTACCAATCATCTTCATCCCTTGATGGAACAACGACTTCTGAACAAGAAGCTTCCTCAGGA
EGD-e_ST35    558 TGTACTAAATTACCAATCATCTTCATCCCTTGATGGAACAACGACTTCTGAACAAGAAGCTTCCTCAGGA
10403S_ST85   561 TGTACTAAATTACCAATCATCTTCATCCCTTGATGGAACAACGACTTCTGAACAAGAAGCTTCATCAGGA
F2365_ST1     558 TGTTCTAAATTACCAATCATCTTCATCCCTTGATGGAACAACGACTTCTGAACAAGAAGCTTCCTCAGGA
P12_10_ST155  561 TGTACTAAATTACCAATCATCTTCATCCCTTGATGGAACAACGACTTCTGAACAAGAAGCTTCATCAGGA

ScottA_ST2    628 TCTGGTGTTATTTATAAAAAGGCAAATGGAAAAGCCTACATCGTAACAAATAATCACGTTGTTGCTGATG
EGD-e_ST35    628 TCTGGTGTTATTTATAAAAAGGCAAATGGAAAAGCCTACATCGTAACAAATAATCACGTTGTTGCGGATG
10403S_ST85   631 TCTGGTGTTATTTATAAAAAGGCAAATGGAAAAGCCTACATCGTAACAAATAATCACGTTGTTGCTGATG
F2365_ST1     628 TCTGGTGTTATTTATAAAAAGGCAAATGGAAAAGCCTACATCGTAACAAATAATCACGTTGTTGCTGATG
P12_10_ST155  631 TCTGGTGTTATTTATAAAAAGGCAAATGGAAAAGCCTACATCGTAACAAATAATCACGTTGTTGCTGATG

ScottA_ST2    698 CAAATAAATTAGAAGTAACTTTTACAAACGGTAAAAAATCCGAAGCAAAATTACTAGGGACAGACGAATG
EGD-e_ST35    698 CAAATAAATTAGAAGTAACTTTTACAAACGGTAAAAAATCCGAAGCAAAATTACTTGGAACAGATGAATG
10403S_ST85   701 CAAATAAATTAGAAGTAACTTTTACAAACGGTAAAAAATCCGAAGCAAAATTACTCGGAACAGACGAATG
F2365_ST1     698 CAAATAAATTAGAAGTAACTTTTACAAACGGTAAAAAATCCGAAGCAAAATTACTAGGGACAGACGAATG
P12_10_ST155  701 CAAATAAATTAGAAGTAACTTTTACAAACGGTAAAAAATCCGAAGCAAAATTACTCGGAACAGACGAATG

ScottA_ST2    768 GAACGATTTAGCTGTTCTTGAAATTGATGATAAAAATGTTACTACAGTCGCTGCATTCGGCGATTCAGAT
EGD-e_ST35    768 GAACGATTTAGCTGTTCTTGAAATTGATGATAAAAATGTTACTACAGTCGCTGCATTCGGCGATTCAGAT
10403S_ST85   771 GAACGATTTAGCTGTTCTTGAAATTGATGATAAAAATGTTACTACAGTCGCTGCATTCGGCGATTCAGAT
```

```
F2365_ST1      768 GAACGATTTAGCTGTTCTTGAAATTGATGATAAAAATGTTACTACAGTCGCTGCATTCGGCGATTCAGAT
P12_10_ST155   771 GAACGATTTAGCTGTTCTTGAAATTGATGATAAAAATGTTACTACAGTCGCTGCATTCGGCGATTCAGAT

ScottA_ST2     838 TCATTAAAACTTGGTGAACCAGCAATTGCAATTGGTAGCCCACTAGGAACAGAATTTTCCGGTTCTGTAA
EGD-e_ST35     838 TCATTAAAACTTGGTGAACCAGCAATTGCAATTGGTAGCCCACTAGGAACGGAATTTTCCGGTTCTGTAA
10403S_ST85    841 TCATTAAAACTTGGTGAACCAGCAATTGCAATTGGTAGCCCACTAGGAACGGAATTTTCCGGTTCTGTAA
F2365_ST1      838 TCATTAAAACTTGGTGAACCAGCAATTGCAATTGGTAGCCCACTTGGAACAGAATTTTCCGGTTCTGTAA
P12_10_ST155   841 TCATTAAAACTTGGTGAACCAGCAATTGCAATTGGTAGCCCACTAGGAACGGAATTTTCCGGTTCTGTAA

ScottA_ST2     908 CACAAGGTATTATTTCTGGTCTAAACCGTGCAGTACCAGTTGATACAAATGGCGACGGAACAGAAGACTG
EGD-e_ST35     908 CACAAGGTATTATTTCTGGTCTAAACCGTGCAGTACCAGTTGATACAAATGGCGACGGAACAGAAGACTG
10403S_ST85    911 CACAAGGTATTATTTCTGGTCTAAACCGTGCAGTACCAGTTGATACAAATGGCGACGGAACAGAAGACTG
F2365_ST1      908 CACAAGGTATTATTTCTGGTCTAAACCGTGCAGTACCAGTTGATACAAATGGCGACGGAACAGAAGACTG
P12_10_ST155   911 CACAAGGTATTATTTCTGGTCTAAACCGTGCAGTACCAGTTGATACAAATGGCGACGGAACAGAAGACTG

ScottA_ST2     978 GGAAGCAGATGTTATCCAAACAGATGCAGCAATTAACCCTGGTAACAGTGGTGGAGCTTTAATTAATATT
EGD-e_ST35     978 GGAAGCAGATGTTATCCAAACAGATGCAGCAATCAATCCAGGTAACAGTGGTGGAGCTTTAATTAACATT
10403S_ST85    981 GGAAGCAGATGTTATCCAAACAGATGCAGCAATCAATCCAGGTAACAGTGGTGGAGCTTTAATTAACATT
F2365_ST1      978 GGAAGCAGATGTTATCCAAACAGATGCAGCAATTAACCCTGGTAACAGTGGTGGAGCTTTAATTAATATT
P12_10_ST155   981 GGAAGCAGATGTTATCCAAACAGATGCAGCAATCAATCCAGGTAACAGTGGTGGAGCTTTAATTAACATT

ScottA_ST2    1048 GAAGGCCAAGTAATTGGTATTAACTCAATGAAAATTTCGATGGAAAATGTAGAAGGTATTAGCTTTGCAA
EGD-e_ST35    1048 GAAGGCCAAGTAATCGGTATTAACTCAATGAAAATTTCGATGGAAAATGTAGAAGGTATTAGCTTTGCAA
10403S_ST85   1051 GAAGGCCAAGTAATCGGTATCAACTCAATGAAAATTTCGATGGAAAACGTAGAAGGTATTAGCTTTGCAA
F2365_ST1     1048 GAAGGCCAAGTAATTGGTATTAACTCAATGAAAATTTCGATGGAAAATGTAGAAGGTATTAGCTTTGCAA
P12_10_ST155  1051 GAAGGCCAAGTAATCGGTATTAACTCAATGAAAATTTCGATGGAAAATGTAGAAGGTATTAGCTTTGCAA

ScottA_ST2    1118 TTCCAAGTAACACAGTAGAACCAATCATCGAACAACTAGAAACAAAAGGCGAAGTAGAACGTCCATCTCT
EGD-e_ST35    1118 TTCCAAGTAACACAGTAGAACCAATTATCGAACAACTAGAAACAAAAGGCGAAGTAGAACGTCCGTCTCT
10403S_ST85   1121 TTCCAAGTAACACAGTAGAACCAATCATCGAACAACTAGAAACAAAAGGCGAAGTAGAACGTCCATCTCT
F2365_ST1     1118 TTCCAAGTAACACAGTAGAACCAATCATCGAACAACTAGAAACAAAAGGCGAAGTAGAACGTCCATCTCT
P12_10_ST155  1121 TTCCAAGTAACACAGTAGAACCAATTATCGAACAACTAGAAACAAAAGGCGAAGTAGAACGTCCATCTCT

ScottA_ST2    1188 AGGCGTATCCTTACGTGATGTTGATACAATTCCAGAAACACAACAAAAAAATATCTTGAAATTACCTGAT
EGD-e_ST35    1188 AGGTGTATCCTTACGTGACGTTGATACAATTCCAGAAACACAACAAAAAAATATCTTGAAATTACCTGAT
10403S_ST85   1191 AGGTGTATCCTTACGTGACGTTGATACAATTCCAGAAACACAACAAAAAAATATCTTGAAATTACCTGAT
F2365_ST1     1188 AGGCGTATCCTTACGTGATGTTGATACAATTCCAGAAACACAACAAAAAAATATCTTGAAATTACCTGAT
P12_10_ST155  1191 AGGTGTATCCTTACGTGACGTTGATACAATTCCAGAAACACAACAAAAAAATATCTTGAAATTACCTGAT

ScottA_ST2    1258 AGCGTAGATTACGGCGCAATGGTACAACAAGTAGTATCCGGTTCTGCAGCAGACAAAGCAGGCTTGAAAC
EGD-e_ST35    1258 AGCGTAGATTACGGCGCAATGGTACAACAAGTAGTATCCGGTTCTGCAGCAGACAAAGCAGGCTTGAAAC
10403S_ST85   1261 AGCGTAGATTACGGCGCAATGGTACAACAAGTAGTATCCGGTTCTGCAGCAGACAAAGCAGGCTTGAAAC
F2365_ST1     1258 AGCGTAGATTACGGCGCAATGGTACAACAAGTAGTATCCGGTTCTGCAGCAGACAAAGCAGGCTTGAAAC
P12_10_ST155  1261 AGCGTAGATTACGGCGCAATGGTACAACAAGTAGTATCTGGTTCTGCAGCAGACAAAGCAGGCTTGAAAC

ScottA_ST2    1328 AATACGATGTTATTGTTGAACTAAACGGCCAAAAAGTAACAAACTCCATGACATTACGCAAAATTCTATA
EGD-e_ST35    1328 AATACGATGTTATTGTTGAACTAAACGGCCAAAAAGTAACAAACTCCATGACATTACGCAAAATTCTATA
10403S_ST85   1331 AATACGATGTTATTGTTGAACTAAACGGCCAAAAAGTAACAAACTCCATGACATTACGCAAAATTCTATA
F2365_ST1     1328 AATACGATGTTATTGTTGAACTAAACGGCCAAAAAGTAACAAACTCCATGACATTACGCAAAATTCTATA
P12_10_ST155  1331 AATACGATGTAATTGTTGAACTAAACGGCCAAAAAGTAACAAACTCTATGACATTACGCAAAATTCTATA

ScottA_ST2    1398 CGGTAACGACGTGAAAATTGGCGATAAAGTCAAAGTGAAATACTATCGTGACGGTAAAGAAAAATCCACA
EGD-e_ST35    1398 CGGTAACGACGTGAAAATTGGCGATAAAGTCAAAGTAAAATACTATCGTGACGGTAAAGAAAAATCCACA
10403S_ST85   1401 CGGTAACGACGTGAAAATTGGCGATAAAGTCAAAGTAAAATACTATCGTGACGGTAAAGAAAAATCCACA
F2365_ST1     1398 CGGTAACGACGTGAAAATTGGCGATAAAGTCAAAGTGAAATACTATCGTGACGGTAAAGAAAAATCCACA
P12_10_ST155  1401 CGGTAACGACGTGAAAATTGGCGATAAAGTCAAAGTAAAATACTATCGTGACGGTAAAGAAAAATCCACA

ScottA_ST2    1468 GATATTAAATTAGAAGCAGCAAAAACAACTACATGA
EGD-e_ST35    1468 GATATTAAATTAGAAGCAGCAAAAACAACTACATGA
10403S_ST85   1471 GATATTAAATTAGAAGCAGCAAAAACAACTACATGA
F2365_ST1     1468 GATATTAAATTAGAAGCAGCAAAAACAACCACATGA
P12_10_ST155  1471 GATATTAAATTAGAAGCAGCAAAAACAACTACATGA
```

Figure S3B. Alignment of HtrA protein sequences

```
ScottA_ST2      1 MDEKEKNLNENSENESTPKREVEDTLHTPESAQPVQETPIVEGVTPEGEKFAGATEDAAEASSTNAFFEE
EGD-e_ST35      1 MDEKEKNLNENSENESTPKREVEDTLHTPESAQPVQETPIVEGVTPEGEKFAGATEDAAEASSTNAFFEE
10403S_ST85     1 MDEKEKNLNENSENESTPKREVEDTLHTPESAQPVQETPIVEGVTPEGEKFAGATEDAAEASSTNAFFEE
F2365_ST1       1 MDEKEKNLNENSENESTPKREVEDTLHTPESAQPVQETPIVEGVTPEGEKFAGATEDAAEASSTNAFFEE
P12_10_ST155    1 MDEKEKNLNENSENESTPKREVEDTLHTPESAQPVQETPIVEGVTPEGEKFAGATEDAAEASSTNAFFEE

ScottA_ST2     71 ASNKEPEPARPAPGPRRAGTTGGGAVPP-NRVNNGGSGNGNGEPPKRGKHFIGYFLTALIGVIIGGLIIF
EGD-e_ST35     71 ASNKESEPARPAPGPRRAGTTGGGAVPP-NRVNNGGSGNGNGEPPKRGKHFIGYFLTALIGVIIGGLIIF
10403S_ST85    71 ASNKESEPARPAPGPRRAGTTGGGAVPPTNRVNNGGSGNGNGEPPKRGKHFIGYFLTALIGVIIGGLIIF
F2365_ST1      71 ASNKEPEPARPAPGPRRAGTTGGGAVPP-NRVNNGGSGNGNGEPPKRGKHFIGYFLTALIGVIIGGLIIF
P12_10_ST155   71 ASNKESEPARPAPGPRRAGTTGGGAVPPTNRVNNGGSGNGNGEPPKRGKHFIGYFLTALIGVIIGGLIIF

ScottA_ST2    140 FVAWDNGDNADTTSNSNNKATKVEKVSVDTTSDVTKAVDKVQDAVVSVLNYQSSSSLDGTTTSEQEASSG
EGD-e_ST35    140 FVAWDNGDNADTTSNSNNKATKVEKVSVDTTSDVTKAVDKVQDAVVSVLNYQSSSSLDGTTTSEQEASSG
10403S_ST85   141 FVAWDNGDNADTTSNSNNKATKVEKVSVDTTSDVTKAVDKVQDAVVSVLNYQSSSSLDGTTTSEQEASSG
F2365_ST1     140 FVAWDNGDNADTTSNSNNKATKVEKVSVDTTSDVTKAVDKVQDAVVSVLNYQSSSSLDGTTTSEQEASSG
P12_10_ST155  141 FVAWDNGDNADTTSNSNNKATKVEKVSVDTTSDVTKAVDKVQDAVVSVLNYQSSSSLDGTTTSEQEASSG

ScottA_ST2    210 SGVIYKKANGKAYIVTNNHVVADANKLEVTFTNGKKSEAKLLGTDEWNDLAVLEIDDKNVTTVAAFGDSD
EGD-e_ST35    210 SGVIYKKANGKAYIVTNNHVVADANKLEVTFTNGKKSEAKLLGTDEWNDLAVLEIDDKNVTTVAAFGDSD
10403S_ST85   211 SGVIYKKANGKAYIVTNNHVVADANKLEVTFTNGKKSEAKLLGTDEWNDLAVLEIDDKNVTTVAAFGDSD
F2365_ST1     210 SGVIYKKANGKAYIVTNNHVVADANKLEVTFTNGKKSEAKLLGTDEWNDLAVLEIDDKNVTTVAAFGDSD
P12_10_ST155  211 SGVIYKKANGKAYIVTNNHVVADANKLEVTFTNGKKSEAKLLGTDEWNDLAVLEIDDKNVTTVAAFGDSD

ScottA_ST2    280 SLKLGEPAIAIGSPLGTEFSGSVTQGIISGLNRAVPVDTNGDGTEDWEADVIQTDAAINPGNSGGALINI
EGD-e_ST35    280 SLKLGEPAIAIGSPLGTEFSGSVTQGIISGLNRAVPVDTNGDGTEDWEADVIQTDAAINPGNSGGALINI
10403S_ST85   281 SLKLGEPAIAIGSPLGTEFSGSVTQGIISGLNRAVPVDTNGDGTEDWEADVIQTDAAINPGNSGGALINI
F2365_ST1     280 SLKLGEPAIAIGSPLGTEFSGSVTQGIISGLNRAVPVDTNGDGTEDWEADVIQTDAAINPGNSGGALINI
P12_10_ST155  281 SLKLGEPAIAIGSPLGTEFSGSVTQGIISGLNRAVPVDTNGDGTEDWEADVIQTDAAINPGNSGGALINI

ScottA_ST2    350 EGQVIGINSMKISMENVEGISFAIPSNTVEPIIEQLETKGEVERPSLGVSLRDVDTIPETQQKNILKLPD
EGD-e_ST35    350 EGQVIGINSMKISMENVEGISFAIPSNTVEPIIEQLETKGEVERPSLGVSLRDVDTIPETQQKNILKLPD
10403S_ST85   351 EGQVIGINSMKISMENVEGISFAIPSNTVEPIIEQLETKGEVERPSLGVSLRDVDTIPETQQKNILKLPD
F2365_ST1     350 EGQVIGINSMKISMENVEGISFAIPSNTVEPIIEQLETKGEVERPSLGVSLRDVDTIPETQQKNILKLPD
P12_10_ST155  351 EGQVIGINSMKISMENVEGISFAIPSNTVEPIIEQLETKGEVERPSLGVSLRDVDTIPETQQKNILKLPD

ScottA_ST2    420 SVDYGAMVQQVVSGSAADKAGLKQYDVIVELNGQKVTNSMTLRKILYGNDVKIGDKVKVKYYRDGKEKST
EGD-e_ST35    420 SVDYGAMVQQVVSGSAADKAGLKQYDVIVELNGQKVTNSMTLRKILYGNDVKIGDKVKVKYYRDGKEKST
10403S_ST85   421 SVDYGAMVQQVVSGSAADKAGLKQYDVIVELNGQKVTNSMTLRKILYGNDVKIGDKVKVKYYRDGKEKST
F2365_ST1     420 SVDYGAMVQQVVSGSAADKAGLKQYDVIVELNGQKVTNSMTLRKILYGNDVKIGDKVKVKYYRDGKEKST
P12_10_ST155  421 SVDYGAMVQQVVSGSAADKAGLKQYDVIVELNGQKVTNSMTLRKILYGNDVKIGDKVKVKYYRDGKEKST

ScottA_ST2    490 DIKLEAAKTTT
EGD-e_ST35    490 DIKLEAAKTTT
10403S_ST85   491 DIKLEAAKTTT
F2365_ST1     490 DIKLEAAKTTT
P12_10_ST155  491 DIKLEAAKTTT
```

Figure S4A. Alignment of *aut* nucleotide sequences

```
ScottA_ST2           1 ATGATAAATAAAAAGTGGATGAAAATTGTAATGATTCCGATGCTAGTTGT----------
EGD-e_ST35           1 ATGAT---TAAAAAAGTTTTTCATTTTATACTTGTACTGATGTTAAGTGTAAGTATAATA
10403S_ST85          1 ATGAT---TAAAAAAGTTTTTCATTTTATACTTGTACTGATGTTAAGTGTAAGTATAATA
F2365_ST1            1 ATGATAAATAAAAAGTGGATGAAAATTGTAATGATTCCGATGCTAGTTGT----------
L6-13_ST155          1 ATGAT---TAAAAAAGTTTTTCATTTTATACTTGTACTGATGTTAAGTGTAAGTATAATA
MRL-14-00459_ST      1 ATGAT---TAAAAAAGTTTTTCATTTTATACTTGTACTGATGTTAAGTGTAAGTATAATA
LmNG3_ST155          1 ATGAT---TAAAAAAGTTTTTCATTTTATACTTGTACTGATGTTAAGTGTAAGTATAATA

ScottA_ST2          51 --TCCAATGTACGGTTTGACAACTG-----------------------------TTGGCG
EGD-e_ST35          58 CCTCTATTCCATGCTAAAGCAGCTGAAACAACTAATGGAGTAGATAGTAGTGAACAGGAA
10403S_ST85         58 CCTCTATTCCATGCTAAAGCAGCTGAAACAACTAATGGAGTAGATAGTAGTGAACAGGAA
F2365_ST1           51 --TCCAATGTACGGTTTGACAACTG-----------------------------TTGGCG
L6-13_ST155         58 CCTCTATTCCATGCTAAAGCAGCTGAAACAACTAATGGAGTAGATAGTAGAGAACAGGAA
MRL-14-00459_ST     58 CCTCTATTCCATGCTAAAGCAGCTGAAACAACTAATGGAGTAGATAGTAGAGAACAGGAA
LmNG3_ST155         58 CCTCTATTCCATGCTAAAGCAGCTGAAACAACTAATGGAGTAGATAGTAGAGAACAGGAA

ScottA_ST2          80 GACAATTACAAGATT---------------------------------------------
EGD-e_ST35         118 GATAATACCGAAGTTGCGCGTGAAGAAATGCCTCCAGAGTCTGAGGAACCAGTATTCTCG
10403S_ST85        118 GATAATACCGAAGTTGCGCGTGAAGAAATGCCTCCAGAGTCTGAGGAACCAGTATTCTCG
F2365_ST1           80 GACAATTACAAGATT---------------------------------------------
L6-13_ST155        118 GATAATACCGAAGTTGCGCGTGAAGAAATGCCTCCAGAGTCTGAGGAACCAGTATTCTCG
MRL-14-00459_ST    118 GATAATACCGAAGTTGCGCGTGAAGAAATGCCTCCAGAGTCTGAGGAACCAGTATTCTCG
LmNG3_ST155        118 GATAATACCGAAGTTGCGCGTGAAGAAATGCCTCCAGAGTCTGAGGAACCAGTATTCTCG

ScottA_ST2          95 -------------------------------------------CATTAACTGGAGAAAAT
EGD-e_ST35         178 CTTGAACAAAATAGAGATGATGCTATGGCTGCTTTAGTCGTGCCTCAAACTAGAAATAGT
10403S_ST85        178 CTTGAACAAAATAGAGATGATGCTATGGCTGCTTTAGTCGTGCCTCAAACTAGAAATAGT
F2365_ST1           95 -------------------------------------------CATTAACTGGAGAAAAT
L6-13_ST155        178 CTTGAACAAAATAGAGATGATGCTATGGCTGCTTTAGTCGTGCCTCAAACTAGAAATAGT
MRL-14-00459_ST    178 CTTGAACAAAATAGAGATGATGCTATGGCTGCTTTAGTCGTGCCTCAAACTAGAAATAGT
LmNG3_ST155        178 CTTGAACAAAATAGAGATGATGCTATGGCTGCTTTAGTCGTGCCTCAAACTAGAAATAGT

ScottA_ST2         112 TCCTTTGTTAAAGAGGTTGAAGCTGCAACGACAGCATCGCAACAAGCATTTATCGACAAA
EGD-e_ST35         238 TTTTT---------GAGAGCAGCTAGTACTCCGACATTTCAACAAACATTTATAAATTCC
10403S_ST85        238 TTTTT---------GAGAGCAGCTAGTACTCCGACATTTCAACAAACATTTATAAATTCC
F2365_ST1          112 TCCTTTGTTAAAGAGGTTGAAGCTGCAACGACAGCATCGCAACAAGCATTTATCGACAAA
L6-13_ST155        238 TTTTT---------GAGAGCAGCTAGTACTCCGACATTTCAACAAACATTTATAAATTCC
MRL-14-00459_ST    238 TTTTT---------GAGAGCAGCTAGTACTCCGACATTTCAACAAACATTTATAAATTCC
LmNG3_ST155        238 TTTTT---------GAGAGCAGCTAGTACTCCGACATTTCAACAAACATTTATAAATTCC

ScottA_ST2         172 ATAGCACCTGCTGCCCAGGCATCTCAAGAAAAATATCATCTGTTATCTAGTATAACTTTA
EGD-e_ST35         289 ATTTCCACGCAAGCAATGGATTTATGTAAAAAGTACAATTTATATCCATCTGTAATGATT
10403S_ST85        289 ATTTCCACGCAAGCAATGGATTTATGTAAAAAGTACAATTTATATCCATCTGTAATGATT
F2365_ST1          172 ATAGCACCTGCTGCCCAGGCATCTCAAGAAAAATATCATCTGTTATCTAGTATAACTTTA
L6-13_ST155        289 ATTTCCACGCAAGCAATGGATTTATGTAAAAAGTACAATTTATATCCATCTGTAATGATT
MRL-14-00459_ST    289 ATTTCCACGCAAGCAATGGATTTATGTAAAAAGTACAATTTATATCCATCTGTAATGATT
LmNG3_ST155        289 ATTTCCACGCAAGCAATGGATTTATGTAAAAAGTACAATTTATATCCATCTGTAATGATT

ScottA_ST2         232 GCTCAAGCAATTCTAGAATCTGGTTGGGGAAAAAGTGGACTTGCTA---CACAAGGATAT
EGD-e_ST35         349 GCTCAAGCGGCTCTTGAGAGTAACTGGGGAAGAAGTGAATTAGGGAAGGCCCCTAATTAC
10403S_ST85        349 GCTCAAGCGGCTCTTGAGAGTAACTGGGGAAGAAGTGAATTAGGGAAGGCCCCTAATTAC
F2365_ST1          232 GCTCAAGCAATTCTAGAATCTGGTTGGGGAAAAAGTGGACTTGCTA---CACAAGGATAT
L6-13_ST155        349 GCTCAAGCGGCTCTTGAGAGTAACTGGGGAAGAAGTGAATTAGGGAAGGCCCCTAATTAC
MRL-14-00459_ST    349 GCTCAAGCGGCTCTTGAGAGTAACTGGGGAAGAAGTGAATTAGGGAAGGCCCCTAATTAC
LmNG3_ST155        349 GCTCAAGCGGCTCTTGAGAGTAACTGGGGAAGAAGTGAATTAGGGAAGGCCCCTAATTAC

ScottA_ST2         289 AATTTATTTGGTATAAAAGGGAAATATAATGGACAATCAGTTATCATGACAACTTCTGAA
EGD-e_ST35         409 AACTTGTTTGGAATAAAAGGATCATATAATGGCAAAAGTGTAACAATGAAAACTTGGGAA
10403S_ST85        409 AACTTGTTTGGAATAAAAGGATCATATAATGGCAAAAGTGTAACAATGAAAACTTGGGAA
F2365_ST1          289 AATTTATTTGGTATAAAAGGGAAATATAATGGACAATCAGTTATCATGACAACTTCTGAA
L6-13_ST155        409 AACTTGTTTGGAATAAAAGGATCATATAATGGCAAAAGTGTAACAATGAAAACTTGGGAA
MRL-14-00459_ST    409 AACTTGTTTGGAATAAAAGGATCATATAATGGCAAAAGTGTAACAATGAAAACTTGGGAA
LmNG3_ST155        409 AACTTGTTTGGAATAAAAGGATCATATAATGGCAAAAGTGTAACAATGAAAACTTGGGAA
```

```
ScottA_ST2        349 TATGTGAA---CGGTGAGTGGATTAAAATTGATGCTGAATTCCGCAAATACCCTAGCTGG
EGD-e_ST35        469 TATAGTGATTCCAAAGGTTGGTATCAAATCAACGCAAACTTTGCCAAATACCCATCACAC
10403S_ST85       469 TATAGTGATTCCAAGGGTTGGTATCAAATCAACGCAAACTTTGCCAAATACCCATCACAC
F2365_ST1         349 TATGTGAA---CGGTGAGTGGATTAAAATTGATGCTGAATTCCGCAAATACCCTAGCTGG
L6-13_ST155       469 TATAGTGATTCCAAGGGTTGGTATCAAATCAACGCAAACTTTGCCAAATACCCATCACAC
MRL-14-00459_ST   469 TATAGTGATTCCAAGGGTTGGTATCAAATCAACGCAAACTTTGCCAAATACCCATCACAC
LmNG3_ST155       469 TATAGTGATTCCAAGGGTTGGTATCAAATCAACGCAAACTTTGCCAAATACCCATCACAC

ScottA_ST2        406 AATGAATCTGTCACTGACCATACTCTTTTATTAGTGAACGGAACTTCTTGGAATAAAGAC
EGD-e_ST35        529 AAAGAATCTTTAGAAGACAATGCGAAAAAACTTAGAAATGGCCCAAGTTGGGACTCTAGT
10403S_ST85       529 AAAGAATCTTTAGAAGACAATGCGAAAAAACTTAGAAATGGCCCAAGTTGGGACTCTAGT
F2365_ST1         406 AATGAATCTGTCACTGACCATACTCTTTTATTAGTGAACGGAACTTCTTGGAATAAAGAC
L6-13_ST155       529 AAAGAATCTTTAGAAGACAATGCGAAAAAACTTAGAAATGGCCCAAGTTGGGACTCTAGT
MRL-14-00459_ST   529 AAAGAATCTTTAGAAGACAATGCGAAAAAACTTAGAAATGGCCCAAGTTGGGACTCTAGT
LmNG3_ST155       529 AAAGAATCTTTAGAAGACAATGCGAAAAAACTTAGAAATGGCCCAAGTTGGGACTCTAGT

ScottA_ST2        466 TTATATAAGAAAGT------TGTCGACGCAACGGATTATAAAGTAGCTGCAATGGAGCTT
EGD-e_ST35        589 TATTACAAAGGTGCATGGCGCGAGAATGCAAAAACATACAAAGATGCGACTGCATGGTTA
10403S_ST85       589 TATTACAAAGGTGCATGGCGCGAGAATGCAAAAACATACAAAGATGCGACTGCATGGTTA
F2365_ST1         466 TTATATAAGAAAGT------TGTCGACGCAACGGATTATAAAGTAGCTGCAATGGAGCTT
L6-13_ST155       589 TATTACAAAGGTGCATGGCGCGAGAATGCAAAAACATACAAAGATGCGACTGCATGGTTA
MRL-14-00459_ST   589 TATTACAAAGGTGCATGGCGCGAGAATGCAAAAACATACAAAGATGCGACTGCATGGTTA
LmNG3_ST155       589 TATTACAAAGGTGCATGGCGCGAGAATGCAAAAACATACAAAGATGCGACTGCATGGTTA

ScottA_ST2        520 CAAAAAGCTGGATATGCAACCTCTCCTACATATGGTGCTAGCTTAATTCAAGTAATTGAG
EGD-e_ST35        649 CAGGGA---CGTTATGCAACGGACAACACATATGCTTCTAAGCTAAATACGCTAATTTCT
10403S_ST85       649 CAGGGA---CGTTATGCAACGGACAACACATATGCTTCTAAGCTAAATACGCTAATTTCT
F2365_ST1         520 CAAAAAGCTGGATATGCAACCTCTCCTACATATGGTGCTAGCTTAATTCAAGTAATTGAG
L6-13_ST155       649 CAGGGA---CGTTATGCAACGGACAACACATATGCTTCTAAGCTAAATACGCTAATTTCT
MRL-14-00459_ST   649 CAGGGA---CGTTATGCAACGGACAACACATATGCTTCTAAGCTAAATACGCTAATTTCT
LmNG3_ST155       649 CAGGGA---CGTTATGCAACGGACAACACATATGCTTCTAAGCTAAATACGCTAATTTCT

ScottA_ST2        580 AATTATGATTTAGCCAAATATGATGTTTTATACGACAAAATTCTTACTCAAAAATCCACT
EGD-e_ST35        706 TCATATAATTTGACTCAATATGATACTCTGTACGATACGATTAAACAACAAAAAAATGTT
10403S_ST85       706 TCATATAATTTGACTCAATATGATACTCTGTACGATACGATTAAACAACAAAAAAATGTT
F2365_ST1         580 AATTATGATTTAGCCAAATATGATGTTTTATACGACAAAATTCTTACTCAAAAATCCACT
L6-13_ST155       706 TCATATAATTTGACTCAATATGATACTCTGTACGATACGATTAAACAACAAAAAAATGTT
MRL-14-00459_ST   706 TCATATAATTTGACTCAATATGATACTCTGTACGATACGATTAAACAACAAAAAAATGTT
LmNG3_ST155       706 TCATATAATTTGACTCAA------------------------------------------

ScottA_ST2        640 TCCGGAAAAGCAACTGTTACAAGTCCGACTGGAAATGGTGTATGGACTTTACCGTATAAA
EGD-e_ST35        766 TCTGAAGATGCTAAAGTAGTTAAAGCAGATGGGCATGGTGTTTATAGTGGAATTTACAAT
10403S_ST85       766 TCTGAAGATGCTAAAGTAGTTAAAGCAGATGGGCATGGTGTTTATAGTGGAATTTACAAT
F2365_ST1         640 TCCGGAAAAGCAACTGTTACAAGTCCGACTGGAAATGGTGTATGGACTTTACCGTATAAA
L6-13_ST155       766 TCTGAAGATGCTAAAGTAGTTAAAGCAGATGGGCATGGTGTTTATAGTGGAATTTACAAT
MRL-14-00459_ST   766 TCTGAAGATGCTAAAGTAGTTAAAGCAGATGGGCATGGTGTTTATAGTGGAATTTACAAT
LmNG3_ST155       724 ------------------------------------------------------------

ScottA_ST2        700 GTAAAAGGAGTGCAATCTGTTAGTCCAGCTAGCA------CATACGCTAACAAGGATATC
EGD-e_ST35        826 ACGTCTGCAGCCAGTGCGAAAAAGTTATCTACTGGAGCGCCTTACAACAATAAAGATGTA
10403S_ST85       826 ACGTCTGCAGCCAGTGCGAAAAAGTTATCTACTGGAGCGCCTTACAACAACAAAGATGTA
F2365_ST1         700 GTAAAAGGAGTGCAATCTGTTAGTCCAGCTAGCA------CATACGCTAACAAGGATATC
L6-13_ST155       826 ACGTCTGCAGCCAGTGCGAAAAAGTTATCTACTGGAGCGCCTTACAACAATAAAGATGTA
MRL-14-00459_ST   826 ACGTCTGCAGCCAGTGCGAAAAAGTTATCTACTGGAGCGCCTTACAACAATAAAGATGTA
LmNG3_ST155       724 ------------------------------------------------------------

ScottA_ST2        754 GATTTAGTATCTGTTGCTACAACAAAGAGAGGTACGTACTATCAATTTAAATATAATGGT
EGD-e_ST35        886 AAAATTTTAAAAGAAGGCACTACAAGCAGAGGAACGTGGGTCCAATTTTCTCTCAATAAT
10403S_ST85       886 AAAATTTTAAAAGAAGGCACTACAAGCAGAGGAACGTGGGTCCAATTTTCTCTCAATAAT
F2365_ST1         754 GATTTAGTATCTGTTGCTACAACAAAGAGAGGTACGTACTATCAATTTAAATATAATGGT
L6-13_ST155       886 AAAATTTTAAAAGAAGGCACTACAAGCAGAGGAACGTGGGTCCAATTTTCTCTCAATAAT
MRL-14-00459_ST   886 AAAATTTTAAAAGAAGGCACTACAAGCAGAGGAACGTGGGTCCAATTTTCTCTCAATAAT
LmNG3_ST155       724 ------------------------------------------------------------
```

```
ScottA_ST2        814 AAAGTAGTTGGTTGGGTAGATGGCAAAGCATTAACTATTTATGATAGTGTCAATTATGAT
EGD-e_ST35        946 AAAGTAATCGGCTGGATGGATAAACGCGCATTTGTCTATTATCCAAAAGCAACAAATGTA
10403S_ST85       946 AAAGTAATCGGCTGGATGGATAAACGCGCATTTGTCTATTATCCAAAAGCAACAAATGTA
F2365_ST1         814 AAAGTAGTTGGTTGGGTAGATGGCAAAGCATTAACTATTTATGATAGTGTCAATTATGAT
L6-13_ST155       946 AAAGTAATCGGCTGGATGGATAAACGCGCATTTGTCTATTATCCAAAAGCAACAAATGTA
MRL-14-00459_ST   946 AAAGTAATCGGCTGGATGGATAAACGCGCATTTGTCTATTATCCAAAAGCAACAAATGTA
LmNG3_ST155       724 ------------------------------------------------------------

ScottA_ST2        874 AAAGTAAATGTCGGACGTGCTAAAATTACTAGCCCAGTAAGTAACGGTATCTGGTCTAAA
EGD-e_ST35       1006 AAAACGCTTAACCTAACAGGTAAAATCACTGCTGGATCTACTAATGGTTTATGGTCTGAG
10403S_ST85      1006 AAAACGCTTAACCTAACAGGTAAAATCACTGCTGGATCTACTAATGGTTTATGGTCTGAG
F2365_ST1         874 AAAGTAAATGTCGGACGTGCTAAAATTACTAGCCCAGTAAGTAACGGTATCTGGTCTAAA
L6-13_ST155      1006 AAAACGCTTAACCTAACAGGTAAAATCACTGCTGGATCTACTAATGGTTTATGGTCTGAG
MRL-14-00459_ST  1006 AAAACGCTTAACCTAACAGGTAAAATCACTGCTGGATCTACTAATGGTTTATGGTCTGAG
LmNG3_ST155       724 ------------------------------------------------------------

ScottA_ST2        934 ---CCATACAATGTTTATGGAAGAGAATTTGTTACGAATGCAACAACTTACGCACAACAA
EGD-e_ST35       1066 GTTCCAGGTACAGTGAATGCGAAAAAATTAGCTACAACAGCTGGTGCTTACCAAAATAAA
10403S_ST85      1066 GTTCCAGGTACAGTGAATGCGAAAAAATTAGCTACAACAGCTGGTGCTTACCAAAATAAA
F2365_ST1         934 ---CCATACAATGTTTATGGAAGAGAATTTGTTACGAATGCAACAACTTACGCACAACAA
L6-13_ST155      1066 GTTCCAGGTACAGTGAATGCGAAAAAATTAGCTACAACAGCTGGTGCTTACCAAAATAAA
MRL-14-00459_ST  1066 GTTCCAGGTACAGTGAATGCGAAAAAATTAGCTACAACAGCTGGTGCTTACCAAAATAAA
LmNG3_ST155       724 ------------------------------------------------------------

ScottA_ST2        991 GAAATTAAACTTTTACGCGAAGCACAAACTGCTAAAGGTACTTATTACCAATTTAGCATA
EGD-e_ST35       1126 GATGCTAAAATTATTAAGCAAGGTCAAATTAGTGGCCGAACTTACTATCAATTCCAAGTA
10403S_ST85      1126 GATGCTAAAATTATTAAGCAAGGTCAAATTAGTGGCCGAACTTACTATCAATTCCAAGTA
F2365_ST1         991 GAAATTAAACTTTTACGCGAAGCACAAACTGCTAAAGGTACTTATTACCAATTTAGCATA
L6-13_ST155      1126 GATGCTAAAATTATTAAGCAAGGTCAAATTAGTGGCCGAACTTACTATCAATTCCAAGTA
MRL-14-00459_ST  1126 GATGCTAAAATTATTAAGCAAGGTCAAATTAGTGGCCGAACTTACTATCAATTCCAAGTA
LmNG3_ST155       724 ------------------------------------------------------------

ScottA_ST2       1051 AATAATAAAACTATTGGTTGGATTGATAAACGAGCTCTCACTATCTATCCGTATGATTCC
EGD-e_ST35       1186 GGCGGTAAAACAATTGGTTGGTTGGACGCTCGTGCATTTCATGTTTATGATAAAATCCAA
10403S_ST85      1186 GGCGGTAAAACAATTGGTTGGTTGGACGCTCGTGCATTTCATGTTTATGATAAAATCCAA
F2365_ST1        1051 AATAATAAGACTATTGGTTGGATTGATAAACGAGCTCTCACTATCTATCCGTATGATTCC
L6-13_ST155      1186 GGCGGTAAAACAATTGGTTGGTTGGACGCTCGTGCATTTCATGTTTATGATAAAATCCAA
MRL-14-00459_ST  1186 GGCGGTAAAACAATTGGTTGGTTGGACGCTCGTGCATTTCATGTTTATGATAAAATCCAA
LmNG3_ST155       724 ------------------------------------------------------------

ScottA_ST2       1111 ATTATTTCAAGTAAAAATGTGAACCTTGACGGACAAATTACTAAT----CCAACCGGAAA
EGD-e_ST35       1246 AGCCAGTCAAATGTAAATTGGAATCGAACTATTTTAAATGCCGATAAGCACGGTGTGTAC
10403S_ST85      1246 AGCCAGTCAAATGTAAATTGGAATCGAACTATTTTAAATGCCGATAAACACGGTGTGTAC
F2365_ST1        1111 ATTATTTCAAGTAAAAATGTGAACCTTGACGGACAAATTACTAAT----CCAACCGGAAA
L6-13_ST155      1246 AGCCAGTCAAATGTAAATTGGAATCGAACTATTTTAAATGCCGATAAGCACGGTGTGTAC
MRL-14-00459_ST  1246 AGCCAGTCAAATGTAAATTGGAATCGAACTATTTTAAATGCCGATAAGCACGGTGTGTAC
LmNG3_ST155       724 ------------------------------------------------------------

ScottA_ST2       1167 TGGTATTTGGACTAAAGCGTACAAACTTGAAGGAACAACTTCTGTGGCGCAGGCTACGAA
EGD-e_ST35       1306 TCGGGTGTTTATAATACTTCATCAAGTAGTATGAATAAACTAAGTA----CAGGCGCGAA
10403S_ST85      1306 TCGGGTGTTTATAATACTTCATCAAGTAGTATGAATAAACTAAGTA----CAGGTGCGAA
F2365_ST1        1167 TGGTATTTGGACTAAAGCGTACAAACTTGAAGGAACAACTTCTGTGGCGCAGGCTACGAA
L6-13_ST155      1306 TCGGGTGTTTATAATACTTCATCAAGTAGTATGAATAAACTAAGTA----CAGGTGCGAA
MRL-14-00459_ST  1306 TCGGGTGTTTATAATACTTCATCAAGTAGTATGAATAAACTAAGTA----CAGGTGCGAA
LmNG3_ST155       724 ------------------------------------------------------------

ScottA_ST2       1227 ATATGCAAATAAAGATGTGAAAATCAGCCAACAAATCGAAACTCAACATGGTACTTATTA
EGD-e_ST35       1362 ATATAATAATAAAAAAGTAAAAGTTATTAAGCAAGCCAAGACCGCGCGTGGAACTTGGTA
10403S_ST85      1362 ATATAATAATAAAAAAGTAAAAGTTATTAAGCAAGCCAAGACCGCGCGTGGAACTTGGTA
F2365_ST1        1227 ATATGCAAATAAAGATGTGAAAATCAGCCAACAAATCGAAACTCAACATGGTACTTATTA
L6-13_ST155      1362 ATATAATAATAAAAAAGTAAAAGTTATTAAGCAAGCCAAGACCGCGCGTGGAACTTGGTA
MRL-14-00459_ST  1362 ATATAATAATAAAAAAGTAAAAGTTATTAAGCAAGCCAAGACCGCGCGTGGAACTTGGTA
LmNG3_ST155       724 ------------------------------------------------------------
```

```
ScottA_ST2      1287 CAATATCAGTATCGATGGGAAAGCAATTGGTTGGTTAGATAGAAACGCTATTACACTGTA
EGD-e_ST35      1422 TCAATTCCAAGTAAATGGCAAAACAGTTGGTTGGATGGATTA------TCGTGCATTCTT
10403S_ST85     1422 TCAATTCCAAGTAAATGGCAAAACAGTTGGTTGGATGGATTA------TCGTGCATTCTT
F2365_ST1       1287 CAATATCAGTATCGATGGGAAAGCAATTGGTTGGTTAGATAGAAACGCTATTACACTGTA
L6-13_ST155     1422 TCAATTCCAAGTAAATGGCAAAACAGTTGGTTGGATGGATTA------TCGTGCATTCTT
MRL-14-00459_ST 1422 TCAATTCCAAGTAAATGGCAAAACAGTTGGTTGGATGGATTA------TCGTGCATTCTT
LmNG3_ST155      724 ------------------------------------------------------------

ScottA_ST2      1347 TGATCAAGAGGAATACAATAAAACAGTTGCTATTGACGCAGTAGTAAAAAATGTGAAGGG
EGD-e_ST35      1476 TGACACAATTACCTCCCAAAAAACAATGAATAAAACAGTTACTGTGGGTAATGCCACGAA
10403S_ST85     1476 TGACACAATTACCTCCCAAAAAACAATGAATAAAACAGTTACTGTGGGTAATGCCACGAA
F2365_ST1       1347 TGATCAAGAGGAATACAATAAAACAGTTGCTATTGACGCAGTAGTAAAAAATGTGAAGGG
L6-13_ST155     1476 TGACACAATTACCTCCCAAAAAACAATGAATAAAACAGTTACTGTGGGTAATGCCACGAA
MRL-14-00459_ST 1476 TGACACAATTACCTCCCAAAAAACAATGAATAAAACAGTTACTGTGGGTAATGCCACGAA
LmNG3_ST155      724 ------------------------------------------------------------

ScottA_ST2      1407 TAATGCTGTATGGACAGAACCTTACCGTA---CAGTTGGTACAAAATTAATCGGACCAGC
EGD-e_ST35      1536 TCATGGCGTTTTTGATGGGGTTTATCGAACCTCGCCAACGGTAAAACGAATTTCTTTGGG
10403S_ST85     1536 TCATGGCGTTTTTGATGGAGTTTATCGAACCTCGCCAACGGTAAAACGAATTTCTTTGGG
F2365_ST1       1407 TAATGCTGTATGGACAGAACCTTACCGTA---CAGTTGGTACAAAATTAATCGGACCAGC
L6-13_ST155     1536 TCATGGCGTTTTTGATGGGGTTTATCGAACCTCGCCAACGGTAAAACGAATTTCTTTGGG
MRL-14-00459_ST 1536 TCATGGCGTTTTTGATGGGGTTTATCGAACCTCGCCAACGGTAAAACGAATTTCTTTGGG
LmNG3_ST155      724 ------------------------------------------------------------

ScottA_ST2      1464 GGAAACTTACTTGAATAAAGAAGTGGAAGTCGTCCGTGAAGCAAAAACGCCAAAAGGAAC
EGD-e_ST35      1596 GAAACCATACAATAATAAAAAAGTAAAAGTGCTCAAAGAAGCTGTAACAGACCATGCCAC
10403S_ST85     1596 GAAACCATACAATAATAAAAAAGTAAAAGTGCTCAAAGAAGCTGTAACAGACCATGCCAC
F2365_ST1       1464 GGAAACTTACTTGAATAAAGAAGTGGAAGTCGTCCGTGAAGCAAAAACGCCAAAAGGAAC
L6-13_ST155     1596 GAAACCATACAATAATAAAAAAGTAAAAGTGCTCAAAGAAGCTGTAACAGACCATGCCAC
MRL-14-00459_ST 1596 GAAACCATACAATAATAAAAAAGTAAAAGTGCTCAAAGAAGCTGTAACAGACCATGCCAC
LmNG3_ST155      724 ------------------------------------------------------------

ScottA_ST2      1524 TTACTACCAATTTAAATCTGGTGGCAAAGTAATCGGCTGGTTAGATAAAAAAGCTTTCGA
EGD-e_ST35      1656 ATGGGTTCAATTTAAATACGGTAACA---CAACTGCATGGATGGACAAAAAAGCATTTAA
10403S_ST85     1656 ATGGGTTCAATTTAAATACGGTAACA---CAACTGCATGGATGGACAAAAAAGCATTTAA
F2365_ST1       1524 TTACTACCAATTTAAATCTGGTGGCAAAGTAATCGGCTGGTTAGATAAAAAAGCTTTCGA
L6-13_ST155     1656 ATGGGTTCAATTTAAATACGGTAACA---CAACTGCATGGATGGACAAAAAAGCATTTAA
MRL-14-00459_ST 1656 ATGGGTTCAATTTAAATACGGTAACA---CAACTGCATGGATGGACAAAAAAGCATTTAA
LmNG3_ST155      724 ------------------------------------------------------------

ScottA_ST2      1584 TGTATATGACAATATTAATTACAACAAAGCGGTTAATTTAGATGCTGTAGTGGAAAATGT
EGD-e_ST35      1713 -----------ATATTAA------------------------------------------
10403S_ST85     1713 -----------ATATTAA------------------------------------------
F2365_ST1       1584 TGTATATGACAATATTAATTACAACAAAGCGGTTAATTTAGATGCTGTAGTGGAAAATGT
L6-13_ST155     1713 -----------ATATTAA------------------------------------------
MRL-14-00459_ST 1713 -----------ATATTAA------------------------------------------
LmNG3_ST155      724 -----------ATA----------------------------------------------

ScottA_ST2      1644 GACAGGTAATGCAGTTTGGACGGCTCCTTATAAGAGTAAAGGTGTTAAACTTGTTACTTC
EGD-e_ST35           ------------------------------------------------------------
10403S_ST85          ------------------------------------------------------------
F2365_ST1       1644 GACAGGTAATGCAGTTTGGACGGCTCCTTATAAGAGTAAAGGTGTTAAACTTGTTACTTC
L6-13_ST155          ------------------------------------------------------------
MRL-14-00459_ST      ------------------------------------------------------------
LmNG3_ST155          ------------------------------------------------------------

ScottA_ST2      1704 AGCAGCAACCTATAAAGGCAAGGCAACAAAAATAACTCGTGAAGCGCAAACAAGTAGAGG
EGD-e_ST35           ------------------------------------------------------------
10403S_ST85          ------------------------------------------------------------
F2365_ST1       1704 AGCAGCAACCTATAAAGGCAAGGCAACAAAAATAACTCGTGAAGCGCAAACAAGTAGAGG
L6-13_ST155          ------------------------------------------------------------
MRL-14-00459_ST      ------------------------------------------------------------
LmNG3_ST155          ------------------------------------------------------------
```

```
ScottA_ST2       1764 AACATATTACGAGTTTAGTGTTGATGGTAAAGTCATTGGCTGGTTAGATAAAAAAGCTTT
EGD-e_ST35            ------------------------------------------------------------
10403S_ST85           ------------------------------------------------------------
F2365_ST1        1764 AACATATTACGAGTTTAGTGTTGATGGTAAAGTCATTGGCTGGTTAGATAAAAAAGCTTT
L6-13_ST155           ------------------------------------------------------------
MRL-14-00459_ST       ------------------------------------------------------------
LmNG3_ST155           ------------------------------------------------------------


ScottA_ST2       1824 CGATGTATATGACAATATTAATTACAACAAAGCGGTTAACTTAGATGCTGTAGTGGAAAA
EGD-e_ST35            ------------------------------------------------------------
10403S_ST85           ------------------------------------------------------------
F2365_ST1        1824 CGATGTATATGACAATATTAATTACAACAAAGCGGTTAACTTAGATGCTGTAGTGGAAAA
L6-13_ST155           ------------------------------------------------------------
MRL-14-00459_ST       ------------------------------------------------------------
LmNG3_ST155           ------------------------------------------------------------


ScottA_ST2       1884 TGTGACAGGCAACGCAGTTTGGACTGCTCCATATAAGAGTAAGGGTGTTAAATTAGTTAC
EGD-e_ST35            ------------------------------------------------------------
10403S_ST85           ------------------------------------------------------------
F2365_ST1        1884 TGTGACAGGCAACGCAGTTTGGACTGCTCCATATAAGAGTAAGGGTGTTAAATTAGTTAC
L6-13_ST155           ------------------------------------------------------------
MRL-14-00459_ST       ------------------------------------------------------------
LmNG3_ST155           ------------------------------------------------------------


ScottA_ST2       1944 TTCAGCAGCCACATATAAAGATAAAGCAACTAAAATAACTCGAGAAGCGCAAACAAGTAG
EGD-e_ST35            ------------------------------------------------------------
10403S_ST85           ------------------------------------------------------------
F2365_ST1        1944 TTCAGCAGCCACATATAAAGATAAAGCAACTAAAATAACTCGAGAAGCGCAAACAAGTAG
L6-13_ST155           ------------------------------------------------------------
MRL-14-00459_ST       ------------------------------------------------------------
LmNG3_ST155           ------------------------------------------------------------


ScottA_ST2       2004 AGGAACTTACTACGAATTTAGCGTAAACGGCAAAGTAATCGGTTGGTTAGATAAAAAAGC
EGD-e_ST35            ------------------------------------------------------------
10403S_ST85           ------------------------------------------------------------
F2365_ST1        2004 AGGAACTTACTACGAATTTAGCGTAAACGGCAAAGTAATCGGTTGGTTAGATAAAAAAGC
L6-13_ST155           ------------------------------------------------------------
MRL-14-00459_ST       ------------------------------------------------------------
LmNG3_ST155           ------------------------------------------------------------


ScottA_ST2       2064 TTTTGATGTATATGATTCTATTGAGTACAATAAAGCGATTAATATGACTGGATTACTTAG
EGD-e_ST35            ------------------------------------------------------------
10403S_ST85           ------------------------------------------------------------
F2365_ST1        2064 TTTTGATGTATATGATTCTATTGAGTACAATAAAGCGATTAATATGACTGGATTACTTAG
L6-13_ST155           ------------------------------------------------------------
MRL-14-00459_ST       ------------------------------------------------------------
LmNG3_ST155           ------------------------------------------------------------


ScottA_ST2       2124 CAACGCGCCAGGTAATGGCATTTGGACAGAGCCGTATAGAGTTATTGGCACAAAAAATGT
EGD-e_ST35            ------------------------------------------------------------
10403S_ST85           ------------------------------------------------------------
F2365_ST1        2124 CAACGCGCCAGGTAATGGCATTTGGACAGAGCCGTATAGAGTTATTGGCACAAAAAATGT
L6-13_ST155           ------------------------------------------------------------
MRL-14-00459_ST       ------------------------------------------------------------
LmNG3_ST155           ------------------------------------------------------------


ScottA_ST2       2184 AGGACAAGCAACTGCTTATGCTAACAAGACAGTACAGTTGATACGCGAGGCTAAGACTAC
EGD-e_ST35            ------------------------------------------------------------
10403S_ST85           ------------------------------------------------------------
F2365_ST1        2184 AGGACAAGCAACTGCTTATGCTAACAAGACAGTACAGTTGATACGCGAGGCTAAGACTAC
L6-13_ST155           ------------------------------------------------------------
MRL-14-00459_ST       ------------------------------------------------------------
LmNG3_ST155           ------------------------------------------------------------
```

```
ScottA_ST2       2244 ACGTGCAACTTACTATCAAATGAGTGTAAATGGTAAAATAGTTGGTTGGGTAGATAAACG
EGD-e_ST35            ------------------------------------------------------------
10403S_ST85           ------------------------------------------------------------
F2365_ST1        2244 ACGTGCAACTTACTATCAAATGAGTGTAAATGGTAAAATAGTTGGTTGGGTAGATAAACG
L6-13_ST155           ------------------------------------------------------------
MRL-14-00459_ST       ------------------------------------------------------------
LmNG3_ST155           ------------------------------------------------------------

ScottA_ST2       2304 AGCTTTTACAAACGTTAAATAG
EGD-e_ST35            ----------------------
10403S_ST85           ----------------------
F2365_ST1        2304 AGCTTTTACAAACGTTAAATAG
L6-13_ST155           ----------------------
MRL-14-00459_ST       ----------------------
LmNG3_ST155           ----------------------
```

Figure S4B. Alignment of Aut protein sequences

```
ScottA_ST2            1 MI--------------------------------------NKKWMKIVMIPMLVVPMYGLTTVGGQLQDS
EGD-e_ST35            1 MIKKVFHFILVLMLSVSIIPLFHAKAAETTNGVDSSEQEDNTEVAREEMPPESEEPVFSLEQNRDDAMAA
10403S_ST85           1 MIKKVFHFILVLMLSVSIIPLFHAKAAETTNGVDSSEQEDNTEVAREEMPPESEEPVFSLEQNRDDAMAA
F2365_ST1             1 MI--------------------------------------NKKWMKIVMIPMLVVPMYGLTTVGGQLQDS
L6-13_ST155           1 MIKKVFHFILVLMLSVSIIPLFHAKAAETTNGVDSREQEDNTEVAREEMPPESEEPVFSLEQNRDDAMAA
MRL-14-00459_ST       1 MIKKVFHFILVLMLSVSIIPLFHAKAAETTNGVDSREQEDNTEVAREEMPPESEEPVFSLEQNRDDAMAA
LmNG3_ST155           1 MIKKVFHFILVLMLSVSIIPLFHAKAAETTNGVDSREQEDNTEVAREEMPPESEEPVFSLEQNRDDAMAA

ScottA_ST2           33 LT---GENSFVKEVEAATTASQQAFIDKIAPAAQASQEKYHLLSSITLAQAILESGWGKSGLA-TQGYNL
EGD-e_ST35           71 LVVPQTRNSFLR--AASTPTFQQTFINSISTQAMDLCKKYNLYPSVMIAQAALESNWGRSELGKAPNYNL
10403S_ST85          71 LVVPQTRNSFLR--AASTPTFQQTFINSISTQAMDLCKKYNLYPSVMIAQAALESNWGRSELGKAPNYNL
F2365_ST1            33 LT---GENSFVKEVEAATTASQQAFIDKIAPAAQASQEKYHLLSSITLAQAILESGWGKSGLA-TQGYNL
L6-13_ST155          71 LVVPQTRNSFLR--AASTPTFQQTFINSISTQAMDLCKKYNLYPSVMIAQAALESNWGRSELGKAPNYNL
MRL-14-00459_ST      71 LVVPQTRNSFLR--AASTPTFQQTFINSISTQAMDLCKKYNLYPSVMIAQAALESNWGRSELGKAPNYNL
LmNG3_ST155          71 LVVPQTRNSFLR--AASTPTFQQTFINSISTQAMDLCKKYNLYPSVMIAQAALESNWGRSELGKAPNYNL

ScottA_ST2           99 FGIKGKYNGQSVIMTTSEYVNGE-WIKIDAEFRKYPSWNESVTDHTLLLVNGTSWNKDLYKKV--VDATD
EGD-e_ST35          139 FGIKGSYNGKSVTMKTWEYSDSKGWYQINANFAKYPSHKESLEDNAKKLRNGPSWDSSYYKGAWRENAKT
10403S_ST85         139 FGIKGSYNGKSVTMKTWEYSDSKGWYQINANFAKYPSHKESLEDNAKKLRNGPSWDSSYYKGAWRENAKT
F2365_ST1            99 FGIKGKYNGQSVIMTTSEYVNGE-WIKIDAEFRKYPSWNESVTDHTLLLVNGTSWNKDLYKKV--VDATD
L6-13_ST155         139 FGIKGSYNGKSVTMKTWEYSDSKGWYQINANFAKYPSHKESLEDNAKKLRNGPSWDSSYYKGAWRENAKT
MRL-14-00459_ST     139 FGIKGSYNGKSVTMKTWEYSDSKGWYQINANFAKYPSHKESLEDNAKKLRNGPSWDSSYYKGAWRENAKT
LmNG3_ST155         139 FGIKGSYNGKSVTMKTWEYSDSKGWYQINANFAKYPSHKESLEDNAKKLRNGPSWDSSYYKGAWRENAKT

ScottA_ST2          166 YKVAAMELQKAGYATSPTYGASLIQVIENYDLAKYDVLYDKILTQKSTSGKATVTSPTGNGVWTLPYKVK
EGD-e_ST35          209 YKDATAWLQ-GRYATDNTYASKLNTLISSYNLTQYDTLYDTIKQQKNVSEDAKVVKADGHGVYSGIYNTS
10403S_ST85         209 YKDATAWLQ-GRYATDNTYASKLNTLISSYNLTQYDTLYDTIKQQKNVSEDAKVVKADGHGVYSGIYNTS
F2365_ST1           166 YKVAAMELQKAGYATSPTYGASLIQVIENYDLAKYDVLYDKILTQKSTSGKATVTSPTGNGVWTLPYKVK
L6-13_ST155         209 YKDATAWLQ-GRYATDNTYASKLNTLISSYNLTQYDTLYDTIKQQKNVSEDAKVVKADGHGVYSGIYNTS
MRL-14-00459_ST     209 YKDATAWLQ-GRYATDNTYASKLNTLISSYNLTQYDTLYDTIKQQKNVSEDAKVVKADGHGVYSGIYNTS
LmNG3_ST155         209 YKDATAWLQ-GRYATDNTYASKLNTLISSYNLTQI-----------------------------------

ScottA_ST2          236 G--VQSVSPASTYANKDIDLVSVATTKRGTYYQFKYNGKVVGWVDGKALTIYDSVNYDKVNVGRAKITSP
EGD-e_ST35          278 AASAKKLSTGAPYNNKDVKILKEGTTSRGTWVQFSLNNKVIGWMDKRAFVYYPKATNVKTLNLTGKITAG
10403S_ST85         278 AASAKKLSTGAPYNNKDVKILKEGTTSRGTWVQFSLNNKVIGWMDKRAFVYYPKATNVKTLNLTGKITAG
F2365_ST1           236 G--VQSVSPASTYANKDIDLVSVATTKRGTYYQFKYNGKVVGWVDGKALTIYDSVNYDKVNVGRAKITSP
L6-13_ST155         278 AASAKKLSTGAPYNNKDVKILKEGTTSRGTWVQFSLNNKVIGWMDKRAFVYYPKATNVKTLNLTGKITAG
MRL-14-00459_ST     278 AASAKKLSTGAPYNNKDVKILKEGTTSRGTWVQFSLNNKVIGWMDKRAFVYYPKATNVKTLNLTGKITAG
LmNG3_ST155             ----------------------------------------------------------------------

ScottA_ST2          304 VSNGIWSK-PYNVYGREFVTNATTYAQQEIKLLREAQTAKGTYYQFSINNKTIGWIDKRALTIYPYDSII
EGD-e_ST35          348 STNGLWSEVPGTVNAKKLATTAGAYQNKDAKIIKQGQISGRTYYQFQVGGKTIGWLDARAFHV--YDKIQ
10403S_ST85         348 STNGLWSEVPGTVNAKKLATTAGAYQNKDAKIIKQGQISGRTYYQFQVGGKTIGWLDARAFHV--YDKIQ
F2365_ST1           304 VSNGIWSK-PYNVYGREFVTNATTYAQQEIKLLREAQTAKGTYYQFSINNKTIGWIDKRALTIYPYDSII
L6-13_ST155         348 STNGLWSEVPGTVNAKKLATTAGAYQNKDAKIIKQGQISGRTYYQFQVGGKTIGWLDARAFHV--YDKIQ
MRL-14-00459_ST     348 STNGLWSEVPGTVNAKKLATTAGAYQNKDAKIIKQGQISGRTYYQFQVGGKTIGWLDARAFHV--YDKIQ
LmNG3_ST155             ----------------------------------------------------------------------

ScottA_ST2          373 SSKNVNLDGQITNPTGNGIWTKAYKLEGTT--SVAQATKYANKDVKISQQIETQHGTYYNISIDGKAIGW
EGD-e_ST35          416 SQSNVNWNRTILNADKHGVYSGVYNTSSSSMNKLSTGAKYNNKKVKVIKQAKTARGTWYQFQVNGKTVGW
10403S_ST85         416 SQSNVNWNRTILNADKHGVYSGVYNTSSSSMNKLSTGAKYNNKKVKVIKQAKTARGTWYQFQVNGKTVGW
F2365_ST1           373 SSKNVNLDGQITNPTGNGIWTKAYKLEGTT--SVAQATKYANKDVKISQQIETQHGTYYNISIDGKAIGW
L6-13_ST155         416 SQSNVNWNRTILNADKHGVYSGVYNTSSSSMNKLSTGAKYNNKKVKVIKQAKTARGTWYQFQVNGKTVGW
MRL-14-00459_ST     416 SQSNVNWNRTILNADKHGVYSGVYNTSSSSMNKLSTGAKYNNKKVKVIKQAKTARGTWYQFQVNGKTVGW
LmNG3_ST155             ----------------------------------------------------------------------

ScottA_ST2          441 LDRNAI--TLYDQEEYNKTVAIDAVVKNVKGNAVWTEPYRTVGT-KLIGPAETYLNKEVEVVREAKTPKG
EGD-e_ST35          486 MDYRAFFDTITSQKTMNKTVT----VGNATNHGVFDGVYRTSPTVKRISLGKPYNNKKVKVLKEAVTDHA
10403S_ST85         486 MDYRAFFDTITSQKTMNKTVT----VGNATNHGVFDGVYRTSPTVKRISLGKPYNNKKVKVLKEAVTDHA
F2365_ST1           441 LDRNAI--TLYDQEEYNKTVAIDAVVKNVKGNAVWTEPYRTVGT-KLIGPAETYLNKEVEVVREAKTPKG
L6-13_ST155         486 MDYRAFFDTITSQKTMNKTVT----VGNATNHGVFDGVYRTSPTVKRISLGKPYNNKKVKVLKEAVTDHA
MRL-14-00459_ST     486 MDYRAFFDTITSQKTMNKTVT----VGNATNHGVFDGVYRTSPTVKRISLGKPYNNKKVKVLKEAVTDHA
LmNG3_ST155             ----------------------------------------------------------------------

ScottA_ST2          508 TYYQFKSGGKVIGWLDKKAFDVYDNINYNKAVNLDAVVENVTGNAVWTAPYKSKGVKLVTSAATYKGKAT
EGD-e_ST35          552 TWVQFKY-GNTTAWMDKKAFKY------------------------------------------------
10403S_ST85         552 TWVQFKY-GNTTAWMDKKAFKY------------------------------------------------
F2365_ST1           508 TYYQFKSGGKVIGWLDKKAFDVYDNINYNKAVNLDAVVENVTGNAVWTAPYKSKGVKLVTSAATYKGKAT
L6-13_ST155         552 TWVQFKY-GNTTAWMDKKAFKY------------------------------------------------
MRL-14-00459_ST     552 TWVQFKY-GNTTAWMDKKAFKY------------------------------------------------
LmNG3_ST155             ----------------------------------------------------------------------
ScottA_ST2          578 KITREAQTSRGTYYEFSVDGKVIGWLDKKAFDVYDNINYNKAVNLDAVVENVTGNAVWTAPYKSKGVKLV
```

```
EGD-e_ST35             ----------------------------------------------------------------------
10403S_ST85            ----------------------------------------------------------------------
F2365_ST1          578 KITREAQTSRGTYYEFSVDGKVIGWLDKKAFDVYDNINYNKAVNLDAVVENVTGNAVWTAPYKSKGVKLV
L6-13_ST155            ----------------------------------------------------------------------
MRL-14-00459_ST        ----------------------------------------------------------------------
LmNG3_ST155            ----------------------------------------------------------------------

ScottA_ST2         648 TSAATYKDKATKITREAQTSRGTYYEFSVNGKVIGWLDKKAFDVYDSIEYNKAINMTGLLSNAPGNGIWT
EGD-e_ST35             ----------------------------------------------------------------------
10403S_ST85            ----------------------------------------------------------------------
F2365_ST1          648 TSAATYKDKATKITREAQTSRGTYYEFSVNGKVIGWLDKKAFDVYDSIEYNKAINMTGLLSNAPGNGIWT
L6-13_ST155            ----------------------------------------------------------------------
MRL-14-00459_ST        ----------------------------------------------------------------------
LmNG3_ST155            ----------------------------------------------------------------------

ScottA_ST2         718 EPYRVIGTKNVGQATAYANKTVQLIREAKTTRATYYQMSVNGKIVGWVDKRAFTNVK
EGD-e_ST35             ---------------------------------------------------------
10403S_ST85            ---------------------------------------------------------
F2365_ST1          718 EPYRVIGTKNVGQATAYANKTVQLIREAKTTRATYYQMSVNGKIVGWVDKRAFTNVK
L6-13_ST155            ---------------------------------------------------------
MRL-14-00459_ST        ---------------------------------------------------------
LmNG3_ST155            ---------------------------------------------------------
```

Figure S5A. Alignment of *prfA* nucleotide sequences .

red: stop codon, yellow: sequence downstream of *prfA*

```
ScottA_ST2      1 ATGAACGCTCAAGCAGAAGAATTCAAAAAATATTTAGAAACTAACGGGATAAAACCAAAACAATTTCATAAAAAAGAACT
EGD-e_ST35      1 ATGAACGCTCAAGCAGAAGAATTCAAAAAATATTTAGAAACTAACGGGATAAAACCAAAACAATTTCATAAAAAAGAACT
10403S_ST85     1 ATGAACGCTCAAGCAGAAGAATTCAAAAAATATTTAGAAACTAACGGGATAAAACCAAAACAATTTCATAAAAAAGAACT
F2365_ST1       1 ATGAACGCTCAAGCAGAAGAATTCAAAAAATATTTAGAAACTAACGGGATAAAACCAAAACAATTTCATAAAAAAGAACT
CDL65_ST155     1 ATGAACGCTCAAGCAGAAGAATTCAAAAAATATTTAGAAACTAACGGGATAAAACCAAAACAATTTCATAAAAAAGAACT
P06_14_ST155    1 ATGAACGCTCAAGCAGAAGAATTCAAAAAATATTTAGAAACTAACGGGATAAAACCAAAACAATTTCATAAAAAAGAACT
Ro05_ST155      1 ATGAACGCTCAAGCAGAAGAATTCAAAAAATATTTAGAAACTAACGGGATAAAACCAAAACAATTTCATAAAAAAGAACT
Ro09_ST155      1 ATGAACGCTCAAGCAGAAGAATTCAAAAAATATTTAGAAACTAACGGGATAAAACCAAAACAATTTCATAAAAAAGAACT
Ro15_ST155      1 ATGAACGCTCAAGCAGAAGAATTCAAAAAATATTTAGAAACTAACGGGATAAAACCAAAACAATTTCATAAAAAAGAACT

ScottA_ST2     81 TATTTTTAACCAATGGGATCCACAAGAATATTGTATTTTCCTATATGATGGTATTACAAAGCTTACAAGTATTAGTGAGA
EGD-e_ST35     81 TATTTTTAACCAATGGGATCCACAAGAATATTGTATTTTTCTATATGATGGTATCACAAAGCTCACGAGTATTAGCGAGA
10403S_ST85    81 TATTTTTAACCAATGGGATCCACAAGAATATTGTATTTTTCTATATGATGGTATCACAAAGCTCACGAGTATTAGCGAGA
F2365_ST1      81 TATTTTTAACCAATGGGATCCACAAGAATATTGTATTTTCCTATATGATGGTATTACAAAGCTTACAAGTATTAGCGAGA
CDL65_ST155    81 TATTTTTAACCAATGGGATCCACAAGAATATTGTATTTTTCTATATGATGGTATCACAAAGCTCACGAGTATTAGCGAGA
P06_14_ST155   81 TATTTTTAACCAATGGGATCCACAAGAATATTGTATTTTTCTATATGATGGTATCACAAAGCTCACGAGTATTAGCGAGA
Ro05_ST155     81 TATTTTTAACCAATGGGATCCACAAGAATATTGTATTTTTCTATATGATGGTATCACAAAGCTCACGAGTATTAGCGAGA
Ro09_ST155     81 TATTTTTAACCAATGGGATCCACAAGAATATTGTATTTTTCTATATGATGGTATCACAAAGCTCACGAGTATTAGCGAGA
Ro15_ST155     81 TATTTTTAACCAATGGGATCCACAAGAATATTGTATTTTTCTATATGATGGTATCACAAAGCTCACGAGTATTAGCGAGA

ScottA_ST2    161 ACGGGACCATCATGAATTTACAATACTATAAAGGTGCTTTCGTTATAATGTCTGGCTTTATCGATACAGAAACATCGGTT
EGD-e_ST35    161 ACGGGACCATCATGAATTTACAATACTACAAAGGGGCTTTCGTTATAATGTCTGGCTTTATTGATACAGAAACATCGGTT
10403S_ST85   161 ACGGGACCATCATGAATTTACAATACTACAAAGGGGCTTTCGTTATAATGTCTGGCTTTATTGATACAGAAACATCGGTT
F2365_ST1     161 ACGGGACCATCATGAATTTACAATACTATAAAGGGGCTTTCGTTATAATGTCTGGCTTTATCGATACAGAAACATCGGTT
CDL65_ST155   161 ACGGGACCATCATGAATTTACAATACTACAAAGGGGCTTTCGTTATAATGTCTGGCTTTATTGATACAGAAACATCGGTT
P06_14_ST155  161 ACGGGACCATCATGAATTTACAATACTACAAAGGGGCTTTCGTTATAATGTCTGGCTTTATTGATACAGAAACATCGGTT
Ro05_ST155    161 ACGGGACCATCATGAATTTACAATACTACAAAGGGGCTTTCGTTATAATGTCTGGCTTTATTGATACAGAAACATCGGTT
Ro09_ST155    161 ACGGGACCATCATGAATTTACAATACTACAAAGGGGCTTTCGTTATAATGTCTGGCTTTATTGATACAGAAACATCGGTT
Ro15_ST155    161 ACGGGACCATCATGAATTTACAATACTACAAAGGGGCTTTCGTTATAATGTCTGGCTTTATTGATACAGAAACATCGGTT

ScottA_ST2    241 GGCTATTATAATTTAGAAGTCATTAGCGAACAGGCTACCGCATACGTTATCAAAATAAACGAACTAAAAGAACTACTGAG
EGD-e_ST35    241 GGCTATTATAATTTAGAAGTCATTAGCGAGCAGGCTACCGCATACGTTATCAAAATAAACGAACTAAAAGAACTACTGAG
10403S_ST85   241 GGCTATTATAATTTAGAAGTCATTAGCGAGCAGGCTACCGCATACGTTATCAAAATAAACGAACTAAAAGAACTACTGAG
F2365_ST1     241 GGCTATTATAATTTAGAAGTCATTAGCGAACAGGCTACCGCATACGTTATCAAAATAAACGAACTAAAAGAACTACTGAG
CDL65_ST155   241 GGCTATTATAATTTAGAAGTCATTAGCGAGCAGGCTACCGCATACGTTATCAAAATAAACGAACTAAAAGAACTACTGAG
P06_14_ST155  241 GGCTATTATAATTTAGAAGTCATTAGCGAGCAGGCTACCGCATACGTTATCAAAATAAACGAACTAAAAGAACTACTGAG
Ro05_ST155    241 GGCTATTATAATTTAGAAGTCATTAGCGAGCAGGCTACCGCATACGTTATCAAAATAAACGAACTAAAAGAACTACTGAG
Ro09_ST155    241 GGCTATTATAATTTAGAAGTCATTAGCGAGCAGGCTACCGCATACGTTATCAAAATAAACGAACTAAAAGAACTACTGAG
Ro15_ST155    241 GGCTATTATAATTTAGAAGTCATTAGCGAGCAGGCTACCGCATACGTTATCAAAATAAACGAACTAAAAGAACTACTGAG

ScottA_ST2    321 CAAGAATCTTACGCACTTTTTCTATGTTTTCCAAACCCTACAAAAACAAGTTTCATACAGCCTAGCCAAATTTAATGATT
EGD-e_ST35    321 CAAAAATCTTACGCACTTTTTCTATGTTTTCCAAACCCTACAAAAACAAGTTTCATACAGTCTAGCTAAATTTAATGATT
10403S_ST85   321 CAAAAATCTTACGCACTTTTTCTATGTTTTCCAAACCCTACAAAAACAAGTTTCATACAGCCTAGCTAAATTTAATGATT
F2365_ST1     321 CAAGAATCTTACGCACTTTTTCTATGTTTTCCAAACCCTACAAAAACAAGTTTCATACAGCCTAGCCAAATTTAATGATT
CDL65_ST155   321 CAAAAATCTTACGCACTTTTTCTATGTTTTCCAAACCCTACAAAAACAAGTTTCATACAGTCTAGCTAAATTTAATGATT
P06_14_ST155  321 CAAAAATCTTACGCACTTTTTCTATGTTTTCCAAACCCTACAAAAACAAGTTTCATACAGTCTAGCTAAATTTAATGATT
Ro05_ST155    321 CAAAAATCTTACGCACTTTTTCTATGTTTTCCAAACCCTACAAAAACAAGTTTCATACAGTCTAGCTAAATTTAATGATT
Ro09_ST155    321 CAAAAATCTTACGCACTTTTTCTATGTTTTCCAAACCCTACAAAAACAAGTTTCATACAGTCTAGCTAAATTTAATGATT
Ro15_ST155    321 CAAAAATCTTACGCACTTTTTCTATGTTTTCCAAACCCTACAAAAACAAGTTTCATACAGTCTAGCTAAATTTAATGATT

ScottA_ST2    401 TTTCGATTAACGGGAAGCTCGGCTCTATTTGCGGTCAACTTTTAATCCTGACCTATGTGTATGGTAAAGAAACTCCTGAT
EGD-e_ST35    401 TTTCGATTAACGGGAAGCTTGGCTCTATTTGCGGTCAACTTTTAATCCTGACCTATGTGTATGGTAAAGAAACTCCTGAT
10403S_ST85   401 TTTCGATTAACGGGAAGCTTGGCTCTATTTGCGGTCAACTTTTAATCCTGACCTATGTGTATGGTAAAGAAACTCCTGAT
F2365_ST1     401 TTTCGATTAACGGGAAGCTCGGCTCTATTTGCGGTCAACTTTTAATCCTGACCTATGTGTATGGTAAAGAAACTCCTGAT
CDL65_ST155   401 TTTCGATTAACGGGAAGCTTGGCTCTATTTGCGGTCAACTTTTAATCCTGACCTATGTGTATGGTAAAGAAACTCCTGAT
P06_14_ST155  401 TTTCGATTAACGGGAAGCTTGGCTCTATTTGCGGTCAACTTTTAATCCTGACCTATGTGTATGGTAAAGAAACTCCTGAT
Ro05_ST155    401 TTTCGATTAACGGGAAGCTTGGCTCTATTTGCGGTCAACTTTTAATCCTGACCTATGTGTATGGTAAAGAAACTCCTGAT
Ro09_ST155    401 TTTCGATTAACGGGAAGCTTGGCTCTATTTGCGGTCAACTTTTAATCCTGACCTATGTGTATGGTAAAGAAACTCCTGAT
Ro15_ST155    401 TTTCGATTAACGGGAAGCTTGGCTCTATTTGCGGTCAACTTTTAATCCTGACCTATGTGTATGGTAAAGAAACTCCTGAT

ScottA_ST2    481 GGCATCAAGATTACACTGGATAATTTAACAATGCAGGAGTTAGGCTATTCAAGCGGTATCGCACATAGCTCAGCTGTTAG
EGD-e_ST35    481 GGCATCAAGATTACACTGGATAATTTAACAATGCAGGAGTTAGGATATTCAAGTGGCATCGCACATAGCTCAGCTGTTAG
10403S_ST85   481 GGCATCAAGATTACACTGGATAATTTAACAATGCAGGAGTTAGGATATTCAAGTGGCATCGCACATAGCTCAGCTGTTAG
F2365_ST1     481 GGCATCAAGATTACACTGGATAATTTAACAATGCAGGAGTTAGGCTATTCAAGCGGTATCGCACATAGCTCAGCTGTTAG
CDL65_ST155   481 GGCATCAAGATTACACTGGATAATTTAACAATGCAGGAGTTAGGATATTCAAGTGGCATCGCACATAGCTCAGCTGTTAG
P06_14_ST155  481 GGCATCAAGATTACACTGGATAATTTAACAATGCAGGAGTTAGGATATTCAAGTGGCATCGCACATAGCTCAGCTGTTAG
Ro05_ST155    481 GGCATCAAGATTACACTGGATAATTTAACAATGCAGGAGTTAGGATATTCAAGTGGCATCGCACATAGCTCAGCTGTTAG
Ro09_ST155    481 GGCATCAAGATTACACTGGATAATTTAACAATGCAGGAGTTAGGATATTCAAGTGGCATCGCACATAGCTCAGCTGTTAG
Ro15_ST155    481 GGCATCAAGATTACACTGGATAATTTAACAATGCAGGAGTTAGGATATTCAAGTGGCATCGCACATAGCTCAGCTGTTAG
```

```
ScottA_ST2     561 CAGAATTATTTCTAAATTAAAGCAAGAGAAAGTTATCGTGTATAAAAATTCATGCTTTTATGTACAAAATCTTGATTATC
EGD-e_ST35     561 CAGAATTATTTCCAAATTAAAGCAAGAGAAAGTTATCGTGTATAAAAATTCATGCTTTTATGTACAAAATCTTGATTATC
10403S_ST85    561 CAGAATTATTTCCAAATTAAAGCAAGAGAAAGTTATCGTGTATAAAAATTCATGCTTTTATGTACAAAATCTTGATTATC
F2365_ST1      561 CAGAATTATTTCTAAATTAAAGCAAGAGAAAGTTATCGTGTATAAAAATTCATGCTTTTATGTACAAAATCTTGATTATC
CDL65_ST155    561 CAGAATTATTTCCAAATTAAAGCAAGAGAAAGTTATCGTGTATAAAAATTCATGCTTTTATGTACAAAATCTTGATTATC
P06_14_ST155   561 CAGAATTATTTCCAAATTAAAGCAAGAGAAAGTTATCGTGTATAAAAATTCATGCTTTTATGTACAAAATCTTGATTATC
Ro05_ST155     561 CAGAATTATTTCCAAATTAAAGCAAGAGAAAGTTATCGTGTATAAAAATTCATGCTTTTATGTACAAAATCTTGATTATC
Ro09_ST155     561 CAGAATTATTTCCAAATTAAAGCAAGAGAAAGTTATCGTGTATAAAAATTCATGCTTTTATGTACAAAATCTTGATTATC
Ro15_ST155     561 CAGAATTATTTCCAAATTAAAGCAAGAGAAAGTTATCGTGTATAAAAATTCATGCTTTTATGTACAAAATCTTGATTATC

ScottA_ST2     641 TCAAAAGATATGCTCCTAAATTAGATGAATGGTTTTATTTAGCATGTCCTGCTACTTGGGGAAAATTAAATTTAAATCAAA
EGD-e_ST35     641 TCAAAAGATATGCCCCTAAATTAGATGAATGGTTTTATTTAGCATGTCCTGCTACTTGGGGAAAATTAAATTTAAATC-AA
10403S_ST85    641 TCAAAAGATATGCCCCTAAATTAGATGAATGGTTTTATTTAGCATGTCCTGCTACTTGGGGAAAATTAAATTTAAATC-AA
F2365_ST1      641 TCAAAAGATATGCTCCTAAATTAGATGAATGGTTTTATTTAGCATGTCCTGCTACTTGGGGAAAATTAAATTTAAATCAAA
CDL65_ST155    641 TCAAAAGATATGCCCCTAAATTAGATGAATGGTTTTATTTAGCATGTCCTGCTACTTGGGGAAAATT-----AAATC-AA
P06_14_ST155   641 TCAAAAGATATGCCCCTAAATTAGATGAATGGTTTTATTTAGCATGTCCTGCTACTTGGGGAAAATT-----AAATC-AA
Ro05_ST155     641 TCAAAAGATATGCCCCTAAATTAGATGAATGGTTTTATTTAGCATGTCCTGCTACTTGGGGAAAATT-----AAATC-AA
Ro09_ST155     641 TCAAAAGATATGCCCCTAAATTAGATGAATGGTTTTATTTAGCATGTCCTGCTACTTGGGGAAAATT-----AAATC-AA
Ro15_ST155     641 TCAAAAGATATGCCCCTAAATTAGATGAATGGTTTTATTTAGCATGTCCTGCTACTTGGGGAAAATT-----AAATC-AA

ScottA_ST2     721 AAACAGGATGTCTCAATGAGGCATCCTGTTTTATATTTTATTCG------------------------------------
EGD-e_ST35     720 AAACAGTATTCCTCAATGAGGAATACTGTTTTATATTTTATTCG------------------------------------
10403S_ST85    720 AAACAGTATTCCTCAATGAGGAATACTGTTTTATATTTTATTCG------------------------------------
F2365_ST1      721 AAACAGGATGTCTCAATGAGGCATCCTGTTTTATATTTTATTCG------------------------------------
CDL65_ST155    715 AAACAGTATTCCTCAATGAGGAATACTGTTTTATATTTTATTCGAATAAAGAACTTACAGAAGCATTTTCATGAACGCGT
P06_14_ST155   715 AAACAGTATTCCTCAATGAGGAATACTGTTTTATATTTTATTCGAATAAAGAACTTACAGAAGCATTTTCATGAACGCGT
Ro05_ST155     715 AAACAGTATTCCTCAATGAGGAATACTGTTTTATATTTTATTCGAATAAAGAACTTACAGAAGCATTTTCATGAACGCGT
Ro09_ST155     715 AAACAGTATTCCTCAATGAGGAATACTGTTTTATATTTTATTCGAATAAAGAACTTACAGAAGCATTTTCATGAACGCGT
Ro15_ST155     715 AAACAGTATTCCTCAATGAGGAATACTGTTTTATATTTTATTCGAATAAAGAACTTACAGAAGCATTTTCATGAACGCGT

ScottA_ST2         --------------------------------------------------------------------------------
EGD-e_ST35         --------------------------------------------------------------------------------
10403S_ST85        --------------------------------------------------------------------------------
F2365_ST1      801 --------------------------------------------------------------------------------
CDL65_ST155    795 ACGATTGCTTCACCAAGAAGAGCTGCAACAGATAGTTGTTCCATTTTGTCAATCCATTTTTCTTCTGGAAGAGCGATGGA
P06_14_ST155   795 ACGATTGCTTCACCAAGAAGAGCTGCAACAGATAGTTGTTCCATTTTGTCAATCCATTTTTCTTCTGGAAGAGCGATGGA
Ro05_ST155     795 ACGATTGCTTCACCAAGAAGAGCTGCAACAGATAGTTGTTCCATTTTGTCAATCCATTTTTCTTCTGGAAGAGCGATGGA
Ro09_ST155     795 ACGATTGCTTCACCAAGAAGAGCTGCAACAGATAGTTGTTCCATTTTGTCAATCCATTTTTCTTCTGGAAGAGCGATGGA
Ro15_ST155     795 ACGATTGCTTCACCAAGAAGAGCTGCAACAGATAGTTGTTCCATTTTGTCAATCCATTTTTCTTCTGGAAGAGCGATGGA

ScottA_ST2         --------
EGD-e_ST35         --------
10403S_ST85        --------
F2365_ST1          --------
CDL65_ST155    875 GTTTGTAA
P06_14_ST155   875 GTTTGTAA
Ro05_ST155     875 GTTTGTAA
Ro09_ST155     875 GTTTGTAA
Ro15_ST155     875 GTTTGTAA
```

Figure S5B. Alignment of *prfA* amino acid sequences.

```
ScottA_ST2      1 MNAQAEEFKKYLETNGIKPKQFHKKELIFNQWDPQEYCIFLYDGITKLTSISENGTIMNLQYYKGAFVIM
EGD-e_ST35      1 MNAQAEEFKKYLETNGIKPKQFHKKELIFNQWDPQEYCIFLYDGITKLTSISENGTIMNLQYYKGAFVIM
10403S_ST85     1 MNAQAEEFKKYLETNGIKPKQFHKKELIFNQWDPQEYCIFLYDGITKLTSISENGTIMNLQYYKGAFVIM
F2365_ST1       1 MNAQAEEFKKYLETNGIKPKQFHKKELIFNQWDPQEYCIFLYDGITKLTSISENGTIMNLQYYKGAFVIM
CDL65_ST155     1 MNAQAEEFKKYLETNGIKPKQFHKKELIFNQWDPQEYCIFLYDGITKLTSISENGTIMNLQYYKGAFVIM
P06_14_ST155    1 MNAQAEEFKKYLETNGIKPKQFHKKELIFNQWDPQEYCIFLYDGITKLTSISENGTIMNLQYYKGAFVIM
Ro05_ST155      1 MNAQAEEFKKYLETNGIKPKQFHKKELIFNQWDPQEYCIFLYDGITKLTSISENGTIMNLQYYKGAFVIM
Ro09_ST155      1 MNAQAEEFKKYLETNGIKPKQFHKKELIFNQWDPQEYCIFLYDGITKLTSISENGTIMNLQYYKGAFVIM
Ro15_ST155      1 MNAQAEEFKKYLETNGIKPKQFHKKELIFNQWDPQEYCIFLYDGITKLTSISENGTIMNLQYYKGAFVIM

ScottA_ST2     71 SGFIDTETSVGYYNLEVISEQATAYVIKINELKELLSKNLTHFFYVFQTLQKQVSYSLAKFNDFSINGKL
EGD-e_ST35     71 SGFIDTETSVGYYNLEVISEQATAYVIKINELKELLSKNLTHFFYVFQTLQKQVSYSLAKFNDFSINGKL
10403S_ST85    71 SGFIDTETSVGYYNLEVISEQATAYVIKINELKELLSKNLTHFFYVFQTLQKQVSYSLAKFNDFSINGKL
F2365_ST1      71 SGFIDTETSVGYYNLEVISEQATAYVIKINELKELLSKNLTHFFYVFQTLQKQVSYSLAKFNDFSINGKL
CDL65_ST155    71 SGFIDTETSVGYYNLEVISEQATAYVIKINELKELLSKNLTHFFYVFQTLQKQVSYSLAKFNDFSINGKL
P06_14_ST155   71 SGFIDTETSVGYYNLEVISEQATAYVIKINELKELLSKNLTHFFYVFQTLQKQVSYSLAKFNDFSINGKL
Ro05_ST155     71 SGFIDTETSVGYYNLEVISEQATAYVIKINELKELLSKNLTHFFYVFQTLQKQVSYSLAKFNDFSINGKL
Ro09_ST155     71 SGFIDTETSVGYYNLEVISEQATAYVIKINELKELLSKNLTHFFYVFQTLQKQVSYSLAKFNDFSINGKL
Ro15_ST155     71 SGFIDTETSVGYYNLEVISEQATAYVIKINELKELLSKNLTHFFYVFQTLQKQVSYSLAKFNDFSINGKL

ScottA_ST2    141 GSICGQLLILTYVYGKETPDGIKITLDNLTMQELGYSSGIAHSSAVSRIISKLKQEKVIVYKNSCFYVQN
EGD-e_ST35    141 GSICGQLLILTYVYGKETPDGIKITLDNLTMQELGYSSGIAHSSAVSRIISKLKQEKVIVYKNSCFYVQN
10403S_ST85   141 GSICGQLLILTYVYGKETPDGIKITLDNLTMQELGYSSGIAHSSAVSRIISKLKQEKVIVYKNSCFYVQN
F2365_ST1     141 GSICGQLLILTYVYGKETPDGIKITLDNLTMQELGYSSGIAHSSAVSRIISKLKQEKVIVYKNSCFYVQN
CDL65_ST155   141 GSICGQLLILTYVYGKETPDGIKITLDNLTMQELGYSSGIAHSSAVSRIISKLKQEKVIVYKNSCFYVQN
P06_14_ST155  141 GSICGQLLILTYVYGKETPDGIKITLDNLTMQELGYSSGIAHSSAVSRIISKLKQEKVIVYKNSCFYVQN
Ro05_ST155    141 GSICGQLLILTYVYGKETPDGIKITLDNLTMQELGYSSGIAHSSAVSRIISKLKQEKVIVYKNSCFYVQN
Ro09_ST155    141 GSICGQLLILTYVYGKETPDGIKITLDNLTMQELGYSSGIAHSSAVSRIISKLKQEKVIVYKNSCFYVQN
Ro15_ST155    141 GSICGQLLILTYVYGKETPDGIKITLDNLTMQELGYSSGIAHSSAVSRIISKLKQEKVIVYKNSCFYVQN

ScottA_ST2    211 LDYLKRYAPKLDEWFYLACPATWGKLN-------------------------------------------
EGD-e_ST35    211 LDYLKRYAPKLDEWFYLACPATWGKLN-------------------------------------------
10403S_ST85   211 LDYLKRYAPKLDEWFYLACPATWGKLN-------------------------------------------
F2365_ST1     211 LDYLKRYAPKLDEWFYLACPATWGKLN-------------------------------------------
CDL65_ST155   211 LDYLKRYAPKLDEWFYLACPATWGKLNQKQYSSMRNTVLYFIRIKNLQKHFHERVRLLHQEELQQIVVPF
P06_14_ST155  211 LDYLKRYAPKLDEWFYLACPATWGKLNQKQYSSMRNTVLYFIRIKNLQKHFHERVRLLHQEELQQIVVPF
Ro05_ST155    211 LDYLKRYAPKLDEWFYLACPATWGKLNQKQYSSMRNTVLYFIRIKNLQKHFHERVRLLHQEELQQIVVPF
Ro09_ST155    211 LDYLKRYAPKLDEWFYLACPATWGKLNQKQYSSMRNTVLYFIRIKNLQKHFHERVRLLHQEELQQIVVPF
Ro15_ST155    211 LDYLKRYAPKLDEWFYLACPATWGKLNQKQYSSMRNTVLYFIRIKNLQKHFHERVRLLHQEELQQIVVPF

ScottA_ST2        -------------
EGD-e_ST35        -------------
10403S_ST85       -------------
F2365_ST1         -------------
CDL65_ST155   281 CQSIFLLEERWSL
P06_14_ST155  281 CQSIFLLEERWSL
Ro05_ST155    281 CQSIFLLEERWSL
Ro09_ST155    281 CQSIFLLEERWSL
Ro15_ST155    281 CQSIFLLEERWSL
```

Figure S5: Predicted 3D protein structure of PrfA using I-TASSER

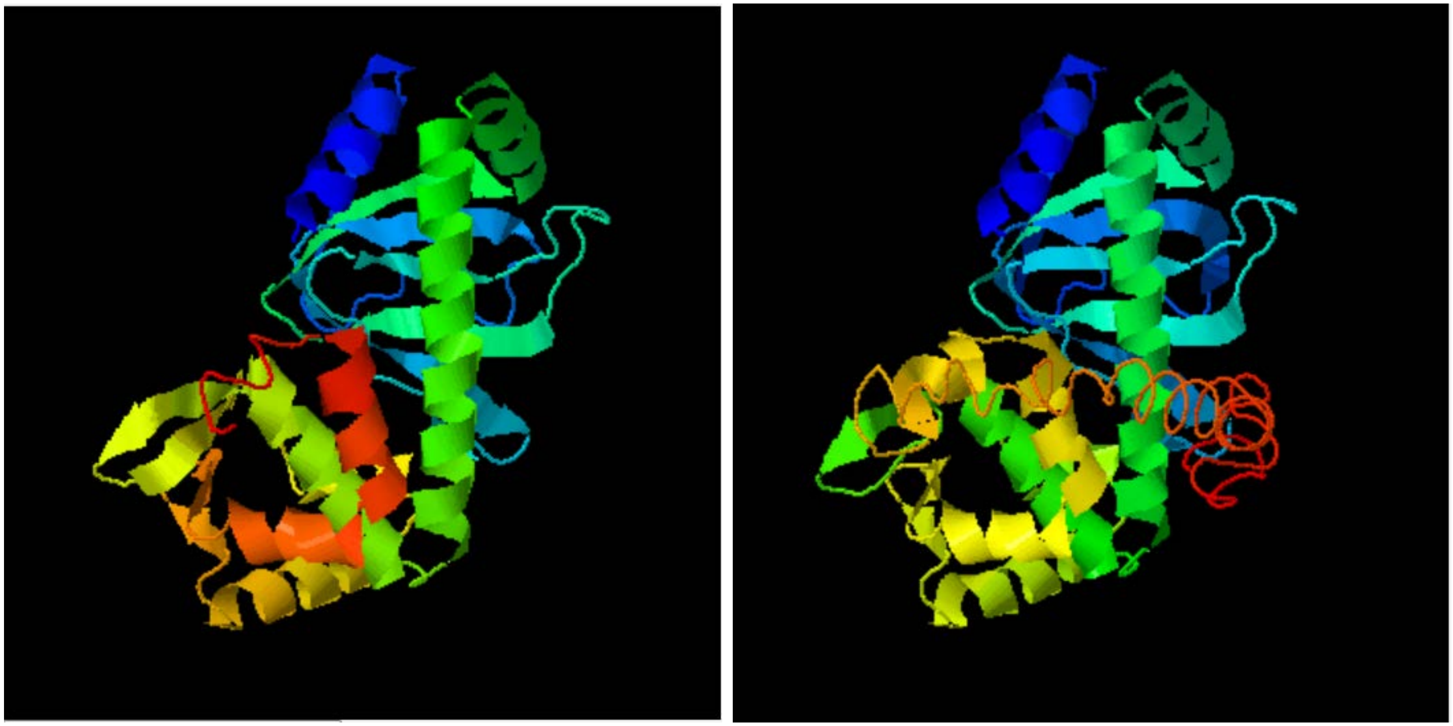

PrfA_EGDe

C-score= 1.07

PrfA_CDL65 (ST155)

C-score= -1.62

C-score is calculated based on the significance of threading template alignments and the convergence parameters of the structure assembly simulations. C-score is typically in the range of [-5, 2], where a C-score of a higher value signifies a model with a higher confidence and vice-versa.

Figure S7. Distribution and variability of stress-associated factors among 130 *L. monocytogenes* ST155 strains.

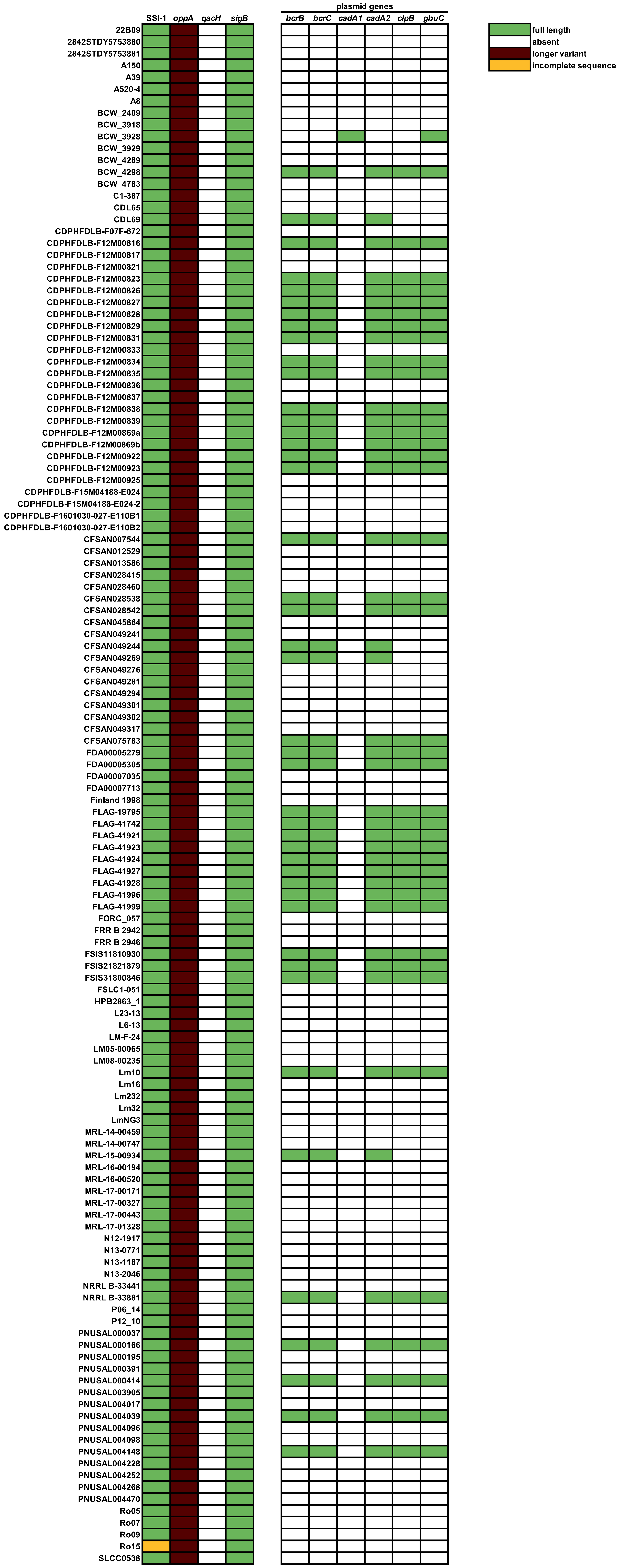

Supplement: Supplementary file 4 — Additional file 4. Figure S1-7. [file 12864_2020_7263_MOESM4_ESM.pdf]
