# Supplementary material for: Virulence characterization and comparative genomics of Listeria monocytogenes sequence type 155 strains
Source: BMC Genomics. 2020 Nov 30;21:847. doi: 10.1186/s12864-020-07263-w (PMC7708227; doi:10.1186/s12864-020-07263-w)
Supplement: Supplementary file 5 — Additional file 5. Table S4. [file 12864_2020_7263_MOESM5_ESM.pdf]

Figure S5: Predicted 3D protein structure of PrfA using I-TASSER

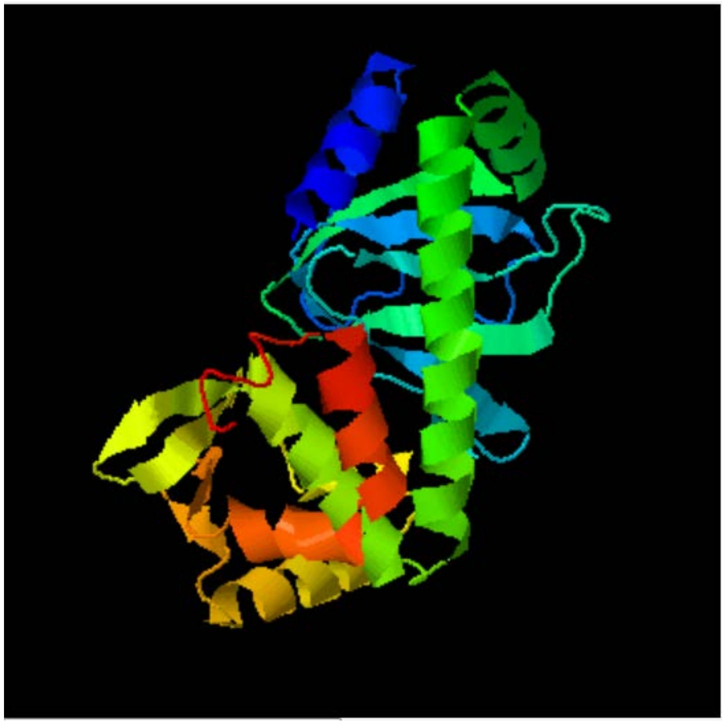

PrfA_EGDe

C-score= 1.07

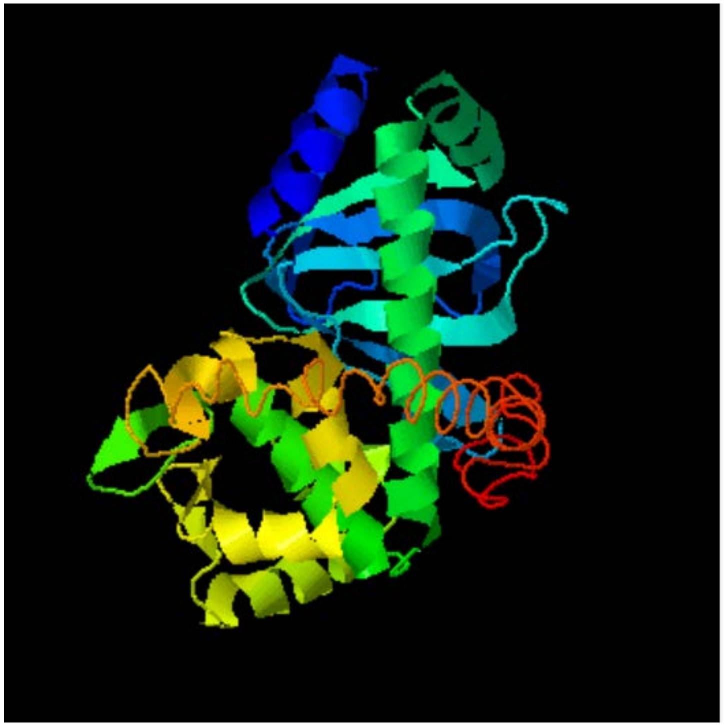

PrfA_CDL65 (ST155)

C-score= -1.62

C-score is calculated based on the significance of threading template alignments and the convergence parameters of the structure assembly simulations. C-score is typically in the range of [-5, 2], where a C-score of a higher value signifies a model with a higher confidence and vice-versa.
