# Supplementary material for: Virulence characterization and comparative genomics of Listeria monocytogenes sequence type 155 strains
Source: BMC Genomics. 2020 Nov 30;21:847. doi: 10.1186/s12864-020-07263-w (PMC7708227; doi:10.1186/s12864-020-07263-w)
Supplement: Supplementary file 7 — Additional file 7. Table S6. [file 12864_2020_7263_MOESM7_ESM.pdf]

Table S5: Presence of selected plasmid genes on *L. monocytogenes* ST155 plasmids.

Strain	Assembly size [bp]	***Npx*** (NADH peroxidase)	**gbuC** (glycine betaine transport binding protein)	***tmr***	***clpB***	***bcrB***	***bcrC***	***cadA1***	***cadA2***	***clpL***
BCW_3928	57,381	yes	yes	no	no	no	no	yes	no	yes
BCW_4298	90,341	yes	yes	yes	yes	yes	yes	no	yes	no
CDPHFDLB-F12M00816	90,160	yes	yes	yes	yes	yes	yes	no	yes	no
CDPHFDLB-F12M00823	90,214	yes	yes	yes	yes	yes	yes	no	yes	no
CDPHFDLB-F12M00826	89,260	yes	yes	yes	yes	yes	yes	no	yes	no
CDPHFDLB-F12M00827	89,260	yes	yes	yes	yes	yes	yes	no	yes	no
CDPHFDLB-F12M00828	90,394	yes	yes	yes	yes	yes	yes	no	yes	no
CDPHFDLB-F12M00829	89,260	yes	yes	yes	yes	yes	yes	no	yes	no
CDPHFDLB-F12M00831	89,260	yes	yes	yes	yes	yes	yes	no	yes	no
CDPHFDLB-F12M00834	89,260	yes	yes	yes	yes	yes	yes	no	yes	no
CDPHFDLB-F12M00835	89,260	yes	yes	yes	yes	yes	yes	no	yes	no
CDPHFDLB-F12M00838	90,376	yes	yes	yes	yes	yes	yes	no	yes	no
CDPHFDLB-F12M00839	89,260	yes	yes	yes	yes	yes	yes	no	yes	no
CDPHFDLB-F12M00869a	90,334	yes	yes	yes	yes	yes	yes	no	yes	no
CDPHFDLB-F12M00869b	87,445	yes	yes	yes	yes	yes	yes	no	yes	no
CDPHFDLB-F12M00922	90,352	yes	yes	yes	yes	yes	yes	no	yes	no
CDPHFDLB-F12M00923	90,381	yes	yes	yes	yes	yes	yes	no	yes	no
CFSAN007544	87,109	yes	yes	yes	yes	yes	yes	no	yes	no
CFSAN028542	92,933	yes	yes	yes	yes	yes	yes	no	yes	no
CFSAN049244	81,589	no	no	yes	no	yes	yes	no	yes	no
CFSAN049269	81,589	no	no	yes	no	yes	yes	no	yes	no
CFSAN075783	89,426	yes	yes	yes	yes	yes	yes	no	yes	no
FDA00005279	88,793	yes	yes	yes	yes	yes	yes	no	yes	no
FDA00005305	88,801	yes	yes	yes	yes	yes	yes	no	yes	no
FLAG-19795	88,391	yes	yes	yes	yes	yes	yes	no	yes	no

FLAG-41742	88,628	yes	yes	yes	yes	yes	yes	no	yes	no
FLAG-41921	89,173	yes	yes	yes	yes	yes	yes	no	yes	no
FLAG-41923	89,257	yes	yes	yes	yes	yes	yes	no	yes	no
FLAG-41924	89,170	yes	yes	yes	yes	yes	yes	no	yes	no
FLAG-41927	89,195	yes	yes	yes	yes	yes	yes	no	yes	no
FLAG-41928	89,272	yes	yes	yes	yes	yes	yes	no	yes	no
FLAG-41996	89,139	yes	yes	yes	yes	yes	yes	no	yes	no
FLAG-41999	89,190	yes	yes	yes	yes	yes	yes	no	yes	no
FSIS11810930	88,100	yes	yes	yes	yes	yes	yes	no	yes	no
FSIS21821879	88,741	yes	yes	yes	yes	yes	yes	no	yes	no
FSIS31800846	89,537	yes	yes	yes	yes	yes	yes	no	yes	no
Lm10	89,025	yes	yes	yes	yes	yes	yes	no	yes	no
NRRL B-33881	88,892	yes	yes	yes	yes	yes	yes	no	yes	no
PNUSAL000166	89,025	yes	yes	yes	yes	yes	yes	no	yes	no
PNUSAL000414	89,025	yes	yes	yes	yes	yes	yes	no	yes	no
PNUSAL004039	81,460	yes	yes	yes	yes	yes	yes	no	yes	no
PNUSAL004148	88,709	yes	yes	yes	yes	yes	yes	no	yes	no
CDL69	80,222	no	no	yes	no	yes	yes	no	yes	no
CFSAN028538	88,901	yes	yes	yes	yes	yes	yes	no	yes	no
MRL-15-00934	81,590	no	no	yes	no	yes	yes	no	yes	no
**Percentage of ST155 plasmids with feature**		**91.10%**	**91.10%**	**97.70%**	**88.80%**	**97.70%**	**97.70%**	**2.20%**	**97.70%**	**2.20%**
