# Supplementary material for: Virulence characterization and comparative genomics of Listeria monocytogenes sequence type 155 strains
Source: BMC Genomics. 2020 Nov 30;21:847. doi: 10.1186/s12864-020-07263-w (PMC7708227; doi:10.1186/s12864-020-07263-w)
Supplement: Supplementary file 8 — Additional file 8. Table S7. [file 12864_2020_7263_MOESM8_ESM.pdf]

Table S6 Primers used in this study.

gene target	method	primer	sequence (5’-3’)
16S rRNA	qRT-PCR	forward	TTAGCTAGTTGGTAGGGTAATGGC
		reverse	CAGTACTTTACGATCCGAAAACCT
inlA	qRT-PCR	forward	TGTGACTGGCGCTTTAATTG
		reverse	TGCCGTCCACATGAAACTTA
inlB	qRT-PCR	forward	TCATGGGAGAGTAACCCAAC
		reverse	TCGGAGGTTTAGGTGCAGTT
actA	qRT-PCR	forward	GATTTATGCGTGCGATGATG
		reverse	TTACCTCGCTTGGTTGCTCT
hly	qRT-PCR	forward	ACGCGGATGAAATCGATAAG
		reverse	TCGCTTTTACGAGAGCACCT
prfA	qRT-PCR	forward	AACCAATGGGATCCACAAGA
		reverse	GATAACGTATGCGGTAGCCT
